# Methods for Addressing Missingness in Electronic Health Record Data for Clinical Prediction Models: Comparative Evaluation

**DOI:** 10.2196/79307

**Published:** 2025-11-14

**Authors:** Jean Digitale, Deborah Franzon, Mark J Pletcher, Charles E McCulloch, Efstathios D Gennatas

**Affiliations:** 1 National Clinician Scholars Program, University of California, San Francisco, San Francisco, CA, United States; 2 Department of Epidemiology and Biostatistics, University of California, San Francisco, 550 16th St, 2nd Floor, San Francisco, CA, 94158, United States, 1 (415) 476-2300; 3 Department of Pediatrics, Benioff Children's Hospital, University of California, San Francisco, San Francisco, CA, United States

**Keywords:** clinical prediction models, imputation, machine learning, missing data, electronic health record

## Abstract

**Background:**

Missing data are a common challenge in electronic health record (EHR)–based prediction modeling. Traditional imputation methods may not suit prediction or machine learning models, and real-world use requires workflows that are implementable for both model development and real-time prediction.

**Objective:**

We evaluated methods for handling missing data when using EHR data to build clinical prediction models for patients admitted to the pediatric intensive care unit (PICU).

**Methods:**

Using EHR data containing missing values from an academic medical center PICU, we generated a synthetic complete dataset. From this, we created 300 datasets with missing data under varying mechanisms and proportions of missingness for the outcomes of (1) successful extubation (binary) and (2) blood pressure (continuous). We assessed strategies to address missing data including simple methods (eg, last observation carried forward [LOCF]), complex methods (eg, random forest multiple imputation), and native support for missing values in outcome prediction models.

**Results:**

Across 886 patients and 1220 intubation events, 18.2% of original data were missing. LOCF had the lowest imputation error, followed by random forest imputation (average mean squared error [MSE] improvement over mean imputation: 0.41 [range: 0.30, 0.50] and 0.33 [0.21, 0.43], respectively). LOCF generally outperformed other imputation methods across outcome metrics and models (mean improvement: 1.28% [range: −0.07%, 7.2%]). Imputation methods showed more performance variability for the binary outcome (balanced accuracy coefficient of variation: 0.042) than the continuous outcome (mean squared error coefficient of variation: 0.001).

**Conclusions:**

Traditional imputation methods for inferential statistics, such as multiple imputation, may not be optimal for prediction models. The amount of missingness influenced performance more than the missingness mechanism. In datasets with frequent measurements, LOCF and native support for missing values in machine learning models offer reasonable performance for handling missingness at minimal computational cost in predictive analyses.

## Introduction

### Background and Significance

Addressing missing data is necessary for developing a clinical prediction model. Electronic health record (EHR) data are a rich data source but present particular challenges. Missing data may result from lack of documentation or measurement [1]. EHR data are generated via clinical care, with values measured at irregular intervals.

Raw EHR data are often transformed into an analytic dataset by binning variables by time. Missing data arise if a variable is not measured within a time window [2]. Measurement frequency (and resultant missingness) is often linked to how abnormal the value is or is expected to be, such that missingness itself may be informative. Given many algorithms require complete data, a principled approach to address missingness is required.

Techniques for handling missing data for inferential models that seek to describe or causally explain are well established. Missingness is traditionally categorized into three mechanisms [3,4]:

1. Missing completely at random (MCAR)—probability of missingness does not depend on variables in the dataset or depends only on observed values of covariates included in the model; for example, a laboratory technician forgets to record results for a patient, unrelated to any characteristics of that patient or their health [5].
2. Missing at random (MAR)—probability of missingness depends on observed values in the data, including the outcome; for example, height is not recorded for a patient but is related to weight and sex of the patient, which are present in the EHR.
3. Missing not at random (MNAR)—probability of missingness depends on unobserved values; for example, no lactate is measured on a patient because the clinician expects it to be normal.

Bias from MNAR can be intractable for inferential models [4]. Given EHR data are likely MNAR, this could be problematic if also true for clinical prediction models. For inferential models, simple strategies, such as complete case analysis, mean imputation, and last observation carried forward, are known to produce biased results [6,7]. Preferred strategies, such as multiple imputation, incorporate uncertainty into imputed values, thereby accurately characterizing uncertainty in parameter estimates.

Literature on handling missing data in prediction modeling is less developed. Unlike inferential models, which focus on bias and precision in parameter estimates, prediction models prioritize improving predictive accuracy and interpretability [4]. Classic statistical imputation methods may be complex to implement for prediction models [8] or less relevant, particularly as medicine advances toward ever more complex machine learning algorithms [4,9,10]. There is little guidance on best practices to address missing data for clinical prediction models [11]. Methods for handling missing data are rarely reported, and complete case analysis is the most common approach [11,12]. This may not only result in bias but also risk significant loss of data in high-dimensional EHR datasets [9]. Machine learning is increasingly being used to address missing data, both as imputation models (eg, random forests) [13] and by natively handling missing data in prediction models themselves, bypassing the need for imputation altogether. Tree-based methods [14] are particularly suited for this task [9]. Yet, few studies have compared classic imputation methods with such built-in strategies in EHR data [9,12].

Real-world application of clinical prediction models presents additional challenges. Many risk models currently in practice require complete data or use imputation methods that may be overly simplistic, limiting their usefulness [15,16]. Implementing models prospectively requires data workflows that can be applied in the same way to both retrospective data to build the model and new data for real-time prediction for individuals [4,10,11,15]. There are no established techniques for managing missing data post-model development. Studies assessing methods for handling missing data in prospective applications on individual patients often used datasets containing only a few predictors [16,17]. These findings may not translate to datasets with more variables because some imputation methods may struggle to handle large numbers of correlated features and binary variables are more likely to be perfectly predicted, leading to overfitting. Furthermore, outcome prediction models after addressing missing data were often standard statistical methods such as logistic regression [15,17] or Cox proportional hazard models [16], when different methods for addressing missingness may be preferable for predictive machine learning models.

### Objective

We used EHR data from a live use case (predicting extubation readiness of children in the pediatric intensive care unit [PICU]) to generate a synthetic complete dataset to evaluate multiple methods for imputation and their effects on predictive performance. We included methods that learn from training data and apply to new data. As the relative performance of methods varies by type of missingness (MAR, MCAR, and MNAR) and proportion of missing values [18], we varied both in our assessment.

## Methods

### Study Population

The study population was patients aged >30 days and <18 years old from the PICU at the University of California, San Francisco (UCSF) Benioff Children’s Hospital intubated between January 1, 2013, and March 31, 2023. Patient encounters were eligible for the sample if the child was intubated for more than 24 hours. We excluded patients intubated for less than 24 hours as they were likely intubated for surgeries, procedures, or other indications and extubated quickly without complications. Children with repeated intubations were eligible for inclusion for each intubation event.

### Data

**Figure 1.**
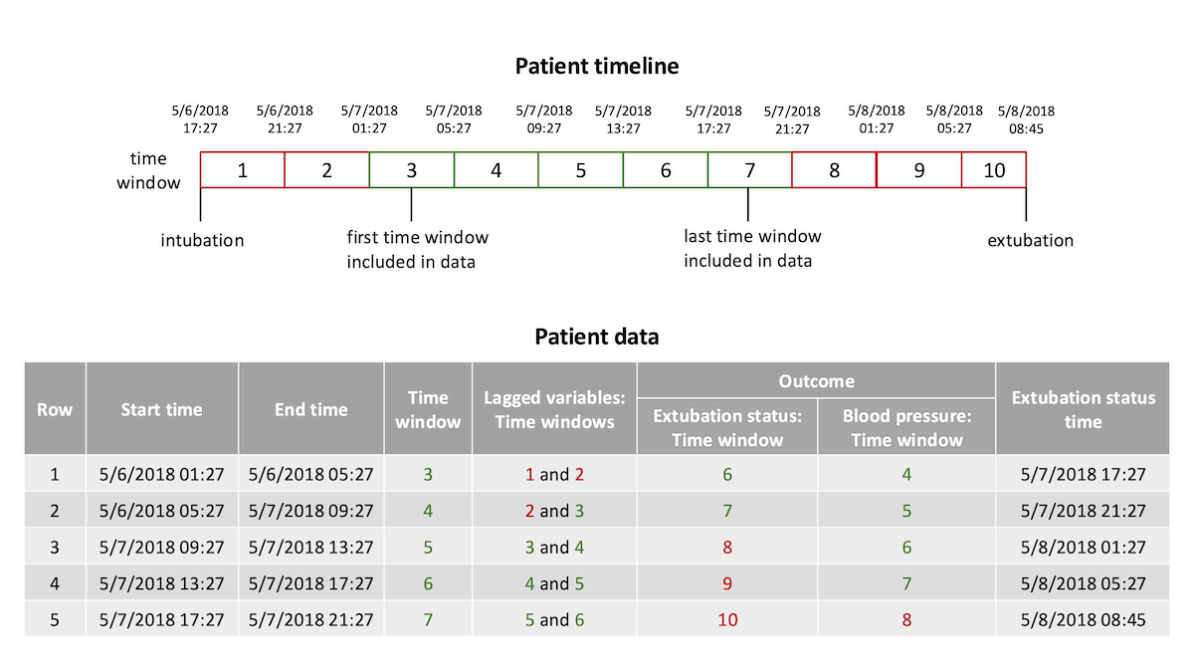
Example patient timeline and resultant data. The example patient timeline depicts 4-hour time windows beginning at intubation and ending at extubation. The table in the figure demonstrates the data resulting from this patient that would be included in the model. This patient was intubated at 17:27 on 5/6/2018 and extubated at 08:45 on 5/8/2018. The first time window included as an observation in the model ended 12 hours after intubation at 05:27 on 5/7/2018. Time-varying data from the prior 2 time windows were included for each observation as lagged variables to capture the trajectory of the patient. The final time window included in the model for this patient is from 17:27 to 21:27 on 5/7/2018. The extubation outcome for each time window was the status 12 hours (or 3 time windows) later. The blood pressure outcome for each time window was the value 4 hours (or 1 time window) later.

We used EHR data extracted from the UCSF Clinical Data Warehouse (updated daily from the real-time EHR). Based on expert opinion, peer-reviewed literature, and group consensus of the UCSF Pediatric Critical Care Research Group, we selected a broad range of clinical, physiologic, and laboratory variables in the EHR that could be relevant to extubation readiness including: vital signs, ventilator settings, laboratory values, medications, neurological status, fluid balance, and other patient characteristics (Table S1 in Multimedia Appendix 1). Raw data were collapsed into 4-hour time windows [1] containing the mean of each numeric or binary variable and mode of each multi-level categorical variable, resulting in 99 variables. Collapsing variables into time windows made imputation more tractable as it increased the probability of at least 1 nonmissing value per time window and more computationally feasible by decreasing the number of observations on which we needed to impute. In practice, nursing assessments are completed at least every 4 hours (with some assessments in the ICU performed hourly); thus, the 4-hour interval is clinically meaningful. The first time window included in the model ended 12 hours after intubation (Figure 1).

The binary outcome of successful extubation was defined as extubation that did not result in reintubation within 48 hours. The extubation outcome assigned to each time window indicated status 12 hours after the end of the time window (Figure 1; Multimedia Appendix 1), as we aimed to predict successful extubation prior to clinician actions indicating they already decided to extubate a patient. A secondary outcome of age-adjusted systolic blood pressure percentile [19] was added to ascertain whether findings were similar for a continuous outcome. The blood pressure outcome assigned to each time window was the value 4 hours after the end of the time window.

**Figure 2.**
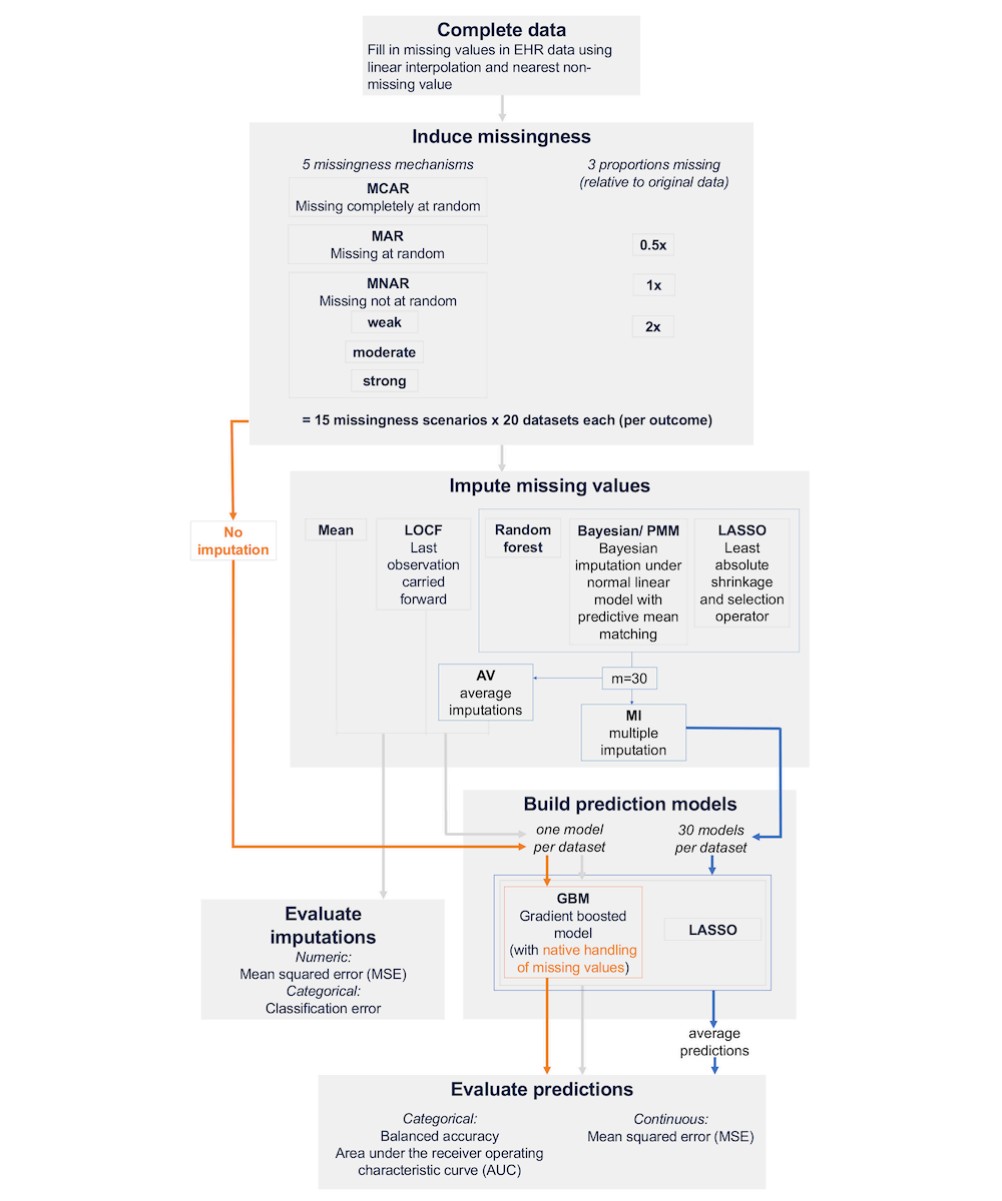
Experimental methods flowchart. EHR: electronic health record.

To generate a synthetic dataset with no missing values (Figure 2), we filled in missing numeric values with linear interpolation between last value observed and next value observed [20]. We filled in all remaining missing values with the nearest nonmissing value. For never observed variables (1.4% of cells), we made reasonable assumptions (eg, we used the standard endotracheal tube [ETT] size formula [age in years/4+4] for pediatrics to fill in missing ETT sizes [21]) and then filled in remaining missingness using the *missRanger* package [22], which implements random forest imputation with predictive mean matching and is optimized for speed and memory efficiency. Analyses were conducted in R version 4.3.2 [23].

### Missingness Mechanisms

We induced missingness in the complete, synthetic dataset to simulate 5 missing data mechanisms:

1. MCAR
2. MAR
3. weak MNAR (twice as much weight given to observed compared with missing variables to determine missingness)
4. moderate MNAR (equal weight given to observed compared with missing variables to determine missingness)
5. strong MNAR (missingness solely based on missing variables)

For each, we created 3 scenarios varying the amount of missingness: approximately 0.5x, 1x, and 2x the percentage of missing cells in the original data, generating 15 total missingness scenarios. We divided variables into 5 groups (Table S1 in Multimedia Appendix 1) to create patterns of missing data. The outcome was allowed to influence missingness in MAR and MNAR scenarios (Multimedia Appendix 1). Using the ampute command in the *mice* package [24], we induced missingness in 20 unique datasets (enough to have a distribution of results, but not exceed our computing power) for each of the 15 scenarios, resulting in 300 datasets per outcome. All datasets for both outcomes contained the same predictor variables.

### Imputation Methods

To incorporate temporal patterns in the predictors, we added values of time-varying variables from the prior 2 lagged time windows to each row for a total of 265 features. These data were structured in wide format, such that each row included the current value and its 2 lags as separate variables. This allowed the imputation models to use all 3 time points to inform each other’s missing values. For simplicity, we treated these repeated measurements as distinct variables in imputation models (rather than using a multilevel model). While some studies suggest this approach yields comparable performance [25], others have reported advantages of multilevel models [26]. Data were split into training (75% of intubations) and test (25% of intubations) sets ordered by date, with earlier intubations included in training and later ones in test. We ensured each patient was only in either the training or test set.

To simulate a real-time prediction workflow, imputation models were built using only training data and excluded the outcome [15]. In total, 6 methods to handle missingness were applied to each amputed dataset (Table 1, Multimedia Appendix 1):

1. Mean: Mean imputation is frequently used in practice [27]. We imputed test set values using the unconditional mean (numeric and binary) or mode (factor) in the training set.
2. Last observation carried forward (LOCF): This simple approach is recommended for imputation in time series data where data are available only before the missing value [20] (true in prospective implementation of clinical decision support). We allowed values to be carried forward indefinitely across time windows.
3. Random forest: Traditional statistical imputation relies on parametric assumptions. However, nonparametric methods, such as this, have been shown to outperform established methods (especially in settings with complex interactions and nonlinear relationships) [28]. *Mice* [29] imputes missing values by building a random forest for each variable, identifying observations in the same terminal node, and sampling a donor value from one of these observations.
4. Bayesian imputation under the normal linear model with predictive mean matching (Bayesian/PMM): PMM is a hot deck method where missing values are imputed from cases with observed values matched according to predictions of the imputation model (here, Bayesian imputation under the normal linear model in *mice* [29]). PMM is robust against model misspecification and ensures imputed values are constrained to the range of observed data [7].
5. Least absolute shrinkage and selection operator (LASSO): Regularized models, such as LASSO, are beneficial to handle multicollinearity and prevent overfitting in high-dimensional data. We used *mice* [29] to fit LASSO-penalized regression models on bootstrap samples of observed data and drew imputed values from the resulting distributions.
6. Native support for missing data in prediction model (no imputation required): Some machine learning algorithms can handle missing values directly, without dropping cases or requiring separate imputation. We used gradient boosted trees for our primary prediction model [30]. The LightGBM package [31] allocates missing values to the bins that optimally minimize loss.

**Table 1.** Methods for handling missing data.

Method	Assumptions	Computational complexity	Limitations	Benefits
Mean imputation	Assumes missing values are similar to the mean	Negligible	Artificially reduces variance Disturbs relationships between variables	Simple to implement Constant imputation creates patterns that machine learning can exploit
Last observation carried forward	Assumes stability over time	Negligible	Data remain missing if no prior measurement exists May not reflect true patient progression Can introduce bias if trends are not stable over time	Simple to implement Reflects how clinicians practice for many variables (assume no changes or re-measure if important)
Random forest	MCAR^a^ or MAR^b^	High computational cost	Requires significant computational resources May not perform well in small datasets Dearth of packages that allow models to impute on new data	Nonparametric method handles complex interactions and nonlinear relationships well Works with mixed data types (categorical and continuous)
Bayesian imputation under the normal linear model with predictive mean matching (Bayesian/PMM)	MCAR or MAR Assumes normality for underlying distribution	High computational cost	Works best with large samples Dearth of packages that allow models to impute on new data	Robust against model misspecification Ensures imputed values are within the range of observed data
Least absolute shrinkage and selection operator (LASSO)	MCAR or MAR	High computational cost	May not capture nonlinear relationships well Dearth of packages that allow models to impute on new data	Handles multicollinearity well Prevents overfitting in high-dimensional data
Native support for missing data in prediction models	Varies by model Methods [12] include surrogate splits and allocating missing values to bins that optimally minimize loss	None for imputation	This capability is not available in all machine learning algorithms Performance depends on algorithm’s internal handling of missing values	No need for explicit imputation

a MCAR: missing completely at random.

b MAR: missing at random.

We used available software for imputation to avoid the need to develop custom software and enable our findings to be more readily applicable to practitioners. We did not include deep-learning methods in our experiment as they were impractical with our relatively small sample size. We generated 30 imputations per dataset using the *mice* package [29] for methods 3‐5. Models built with the training data were then used to impute on the test set. We tested the imputations 2 ways. *Mice* purposefully incorporates uncertainty into imputations because it is advantageous for inferential analysis. First, we averaged the imputations to get a more stable estimate of each missing value to use in a single outcome model. Second, we implemented multiple imputation by estimating 30 outcome models and averaging the predicted probabilities for a final prediction.

### Prediction Model

For the outcome prediction model, we used gradient boosted trees [30]. It is one of the best-performing algorithms in structured data in general and within biomedical datasets [32] and uses all cases in training data, even if they are incomplete. To assess whether imputation method performance was consistent across outcome models, we also compared a LASSO outcome model. However, linear models like LASSO cannot accommodate missing values, so this was only performed on imputed datasets. Outcome models were implemented using the rtemis package [33] with LightGBM [34] and glmnet [35]. We used 5-fold cross-validation in the training set to tune hyperparameters and inverse frequency weighting to upweight the minority class given the data were unbalanced.

### Analysis of Imputation Accuracy

It is established that in imputation for statistical inference, focusing on improving accuracy of the imputations at the cost of correctly incorporating true uncertainty leads to biased and invalid results [7]. However, the relationship between imputation accuracy and prediction model performance is less well-studied. We compared the accuracy of imputations by calculating mean squared error (MSE) for numeric variables and classification error for categorical variables in each dataset and creating box plots. Before calculating MSE, we standardized the variables by dividing by their standard deviations in the complete dataset. To calculate MSE for random forest, Bayesian/PMM, and LASSO, we compared the average of the 30 imputations to the true value. We also assessed whether temporal autocorrelation of each variable was associated with imputation performance across all methods descriptively using scatterplots and quantified using correlation coefficients (Multimedia Appendix 1). In addition, we conducted a sensitivity analysis stratifying imputation error by whether a variable’s values had been missing in the original data (and filled in to create the synthetic complete dataset) versus not originally missing, to evaluate whether this initial filling step influenced the apparent performance of the imputation methods.

To assess the accuracy of imputation for different categories of variables, we built linear models for MSE and classification error. Each observation’s outcome was the error value for a given variable in a given dataset. Each model included imputation type, missingness type, proportion missing data (0.5x, 1x, and 2x original), and variable group (Multimedia Appendix 1). We included all 3-way and 2-way interactions and completed a backward stepwise elimination procedure based on *P* values (included *P*<.05) to determine the final models.

### Analysis of Outcome Model Performance

We assessed the performance of outcome prediction models for extubation with 2 primary metrics: balanced accuracy [36] and area under the receiver operating characteristic curve (AUC) [37]. We present secondary results for sensitivity, specificity, positive predictive value, negative predictive value, and F1 [38]. We assessed the performance of the outcome prediction models for blood pressure with the primary metric of MSE. Secondary results are presented for mean absolute error, root MSE, and *R*^2^ [39]. We compared these graphically to the performance of a model built with the complete data and calculated the coefficient of variation (CV) to assess variability.

### Ethical Considerations

We received ethical approval from the University of California, San Francisco Institutional Review Board (study #17‐23751), which granted a waiver of informed consent. No financial incentives were provided to patients. Patient privacy and confidentiality were protected through secure data storage, restricted access to authorized study personnel, and compliance with institutional and regulatory requirements.

## Results

### Data

The data contained 886 patients and 1220 intubations, 929 (76.1%) of which ended in successful extubation (Table 2). The median duration of intubation in the PICU was 4.4 (IQR 2.2-8.5) days, leading to 50,187 four-hour time windows in the analytic dataset.

**Table 2.** Description of sample^a^.

Variable	Value
Patients	
Total, n	886
Female patients, n (%)	405 (45.7)
Race or ethnicity, n (%)	
Asian	117 (13.9)
Black	67 (8)
Latinx	340 (40.4)
Other	88 (10.5)
White	229 (27.2)
Intubations	
Total, n	1220
Age at intubation (years), median (IQR)	4.3 (1-12.1)
Outcome, n (%)	
Extubation success	929 (76.1)
Extubation failure	100 (8.2)
Death	96 (7.9)
Tracheostomy	36 (3)
Transfer to another unit	25 (2)
ETT^b^ change	34 (2.8)
Duration of intubation (days), median (IQR)	4.4 (2.2-8.5)

a Patients intubated multiple times during the study period may have multiple intubation events included in the sample. The binary outcome of successful extubation for the model collapsed all other outcome categories. Here, extubation failure is defined as reintubation within 48 hours, death is death before or within 48 hours of extubation, and ETT change is an extubation that was immediately and purposefully replaced by another ETT (eg, to change the size).

b ETT: endotracheal tube.

Missingness for each variable in the original data (collapsed into 4 h time windows) varied from 0% (0/56,287; eg, sex, age) to 77% (43,077/56,287; white blood cells) (Table 3; Table S2 in Multimedia Appendix 1). Overall, 18.2% of cells (1,012,668/5,561,767) were missing (Table S3 in Multimedia Appendix 1). After simulating missingness, datasets approximating 0.5x, 1x, and 2x missingness of the original data averaged 9.6%, 18.1%, and 35.9% missing cells, respectively.

**Table 3.** Missingness in original data^a^.

Variable	Number of 4-hour time windows missing variable	Percent of 4-hour time windows missing variable
Age at time of intubation (in days)	0	0.0
Medication: total oral morphine equivalents (mg kg^–1^)	0	0.0
Sex	0	0.0
Intake or output total milliliter over the prior 12 hours kg^–1^	167	0.3
Pulse	420	0.7
Respiratory rate (recorded in vital signs)	1179	2.1
ETT^b^ size	6179	11.0
Respiratory pattern: tachypneic	7290	13.0
PEEP^d^	8975	15.9
Exhaled tidal volume kg^–1^	10,274	18.3
State behavioral scale	15,888	28.2
Secretion amount (categories: none, scant, small, moderate, large, copious)	16,325	29.0
Glasgow coma scale score	25,751	45.7
pH	25,804	45.8
Upper extremity motor response	28,839	51.2
Cough: present	35,254	62.6
White blood cell count	43,077	76.5

a Example variables display the range of proportion of missingness in the original data (N=56,287 4-h time windows; these data include more time windows than the final analytic dataset because they were compiled before excluding time windows that were constructed solely for creating lagged variables). Detailed data for all variables are available in Table S2 in Multimedia Appendix 1.

b EET: endotracheal tube.

c PEEP: positive end-expiratory pressure.

### Imputation Performance

**Figure 3.**
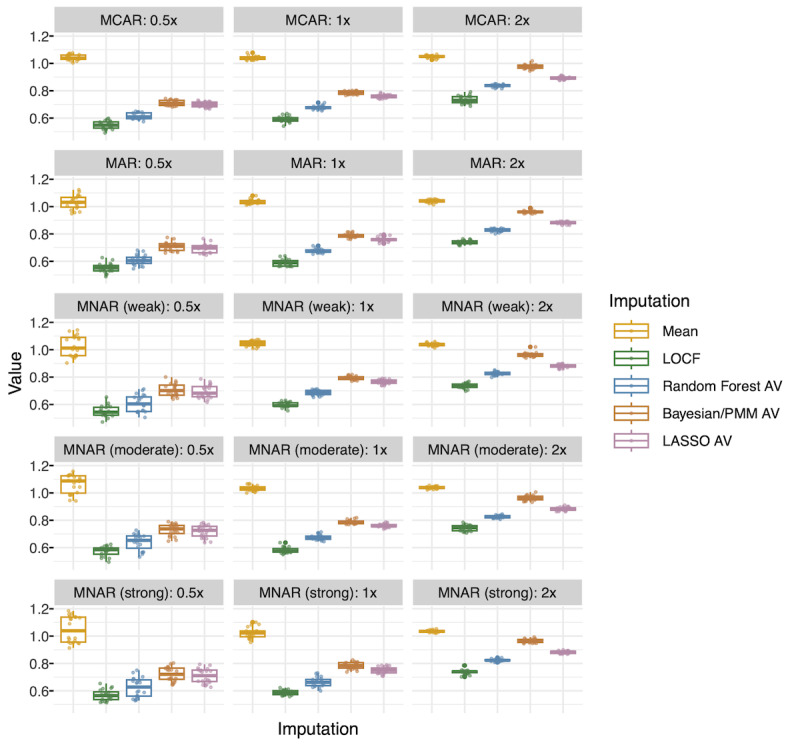
Imputation test performance metrics (extubation): mean squared error. Each point represents mean squared error calculated for 176 numeric variables for 1 of 300 datasets created for the outcome of extubation. There are 20 datasets per missingness scenario and imputation type represented in each box plot. AV: average (average of 30 imputations); Bayesian/PMM: Bayesian imputation under the normal linear model with predictive mean matching; LASSO: least absolute shrinkage and selection operator; LOCF: last observation carried forward; MAR: missing at random; MCAR: missing completely at random; MNAR: missing not at random.

Across 300 datasets with induced missingness per outcome, the same 176 numeric and 6 categorical variables were imputed. Figure 3 presents MSE for datasets for the outcome of extubation.

The results for the outcome of blood pressure (which imputed the same variables) were virtually identical (Multimedia Appendix 2). Performance in the test sets showed that LOCF had the lowest MSE on average in all missingness scenarios for numeric variables (average improvement of MSE compared with mean imputation was 0.41 for the outcome of extubation [range: 0.30, 0.50]). Random forest imputation was consistently second best (0.33 [0.21, 0.43]), followed by LASSO (0.26 [0.15, 0.35]), Bayesian/PMM (0.22 [0.07, 0.34]), and finally, mean imputation (Reference). Performance overall degraded as the proportion of missing data increased, with proportion missing having a greater effect than missingness mechanism. Classification error displayed similar patterns overall for categorical variables (Multimedia Appendix 3). While LOCF and mean imputation did not generally overfit in the training data compared with the test set, all model-based imputation methods overfit the training data, with random forest doing so the least (MultimediaAppendices 4  5). Temporal autocorrelation was negatively associated with imputation error for all methods except mean imputation, which showed little association (*r*=–0.13; Multimedia Appendix 6). The association was strongest for LOCF (*r*=–0.92), followed by random forest (*r*=–0.74). For these, error decreased almost monotonically with increasing autocorrelation, indicating substantially better accuracy for more temporally stable variables.

In a sensitivity analysis, we examined whether the initial filling of originally missing values influenced subsequent comparisons of imputation performance. Overall, MSE was lower for values that were missing in the original data and filled in to create the synthetic complete dataset, especially for LOCF and random forest imputation (2 methods used to fill in the original missingness). Still, both LOCF and random forest continued to achieve the best performance when imputing values not missing in the original data (Multimedia Appendix 7).

In models for mean squared error, a significant 3-way interaction existed between imputation method, proportion missing, and variable group (Tables S4 and S5 in Multimedia Appendix 1) due to the fact that (1) all methods except mean imputation degraded with increased missingness and (2) variables with a response of “select all that apply” were more poorly predicted (Multimedia Appendix 8). There was no interaction between missingness mechanism and (1) imputation method or (2) variable group.

### Prediction Model Performance

#### Gradient Boosted Models

##### Extubation: Balanced accuracy

**Figure 4.**
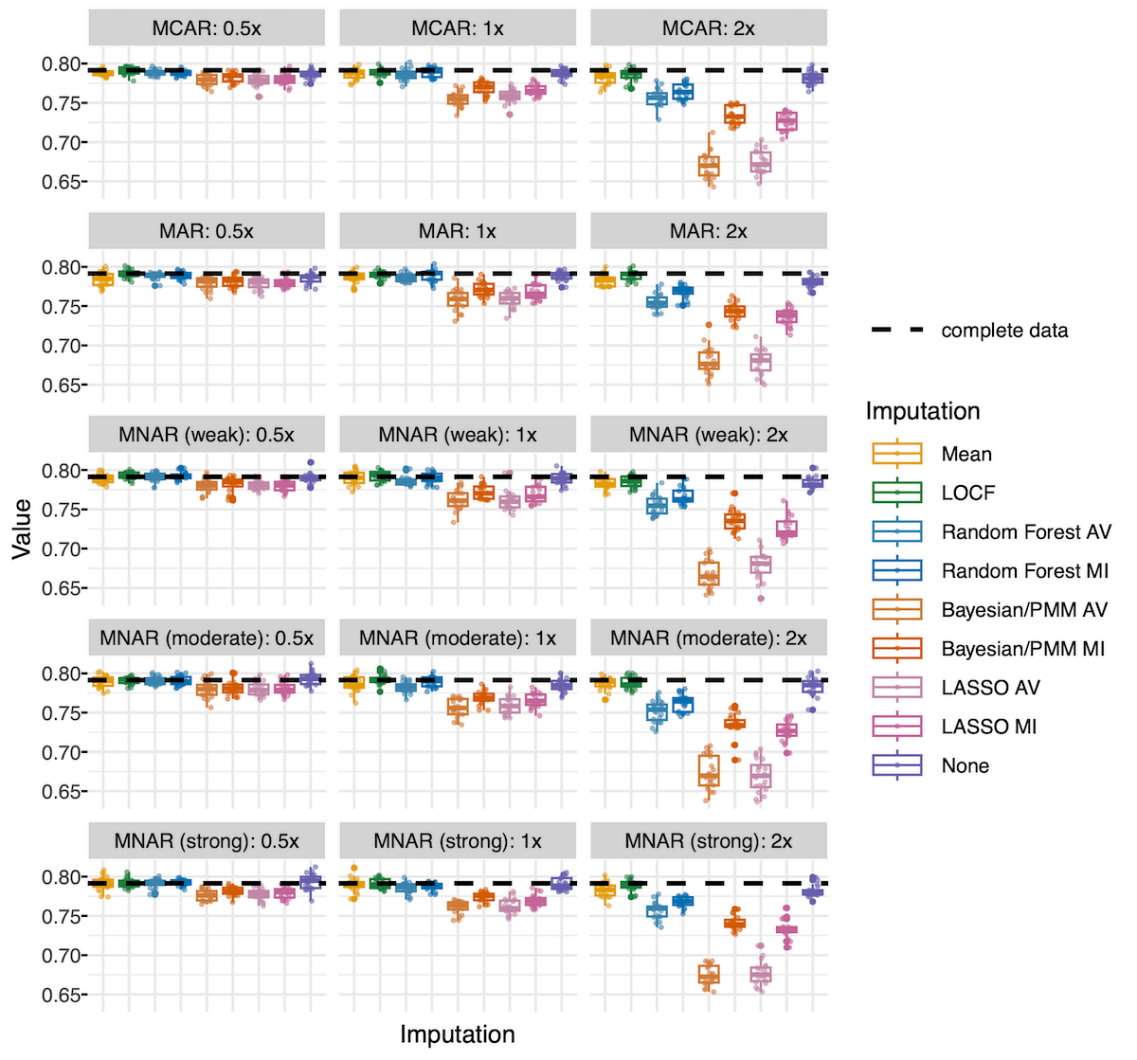
Gradient boosted model test performance (extubation): balanced accuracy. Each point represents balanced accuracy for 1 dataset. There are 20 datasets per missingness scenario and imputation type represented in each box plot. Balanced accuracy in the complete dataset is represented by a dashed line. AV, average (average of 30 imputations); Bayesian/PMM: Bayesian imputation under the normal linear model with predictive mean matching; LASSO: least absolute shrinkage and selection operator; LOCF: last observation carried forward; MAR, missing at random; MCAR: missing completely at random; MI: multiple imputation; MNAR: missing not at random.

For the outcome of extubation using gradient boosted models, balanced accuracy was the highest for LOCF (Figure 4). Mean imputation and no imputation (native support for missing values) performed almost as well. Random forest (both averaged and multiple imputation) also performed well at 0.5x and 1x missingness, but its performance degraded at 2x missingness. The amount of missingness was more influential than the missingness mechanism (Table S6 in Multimedia Appendix 1).

##### Extubation: AUC

**Figure 5.**
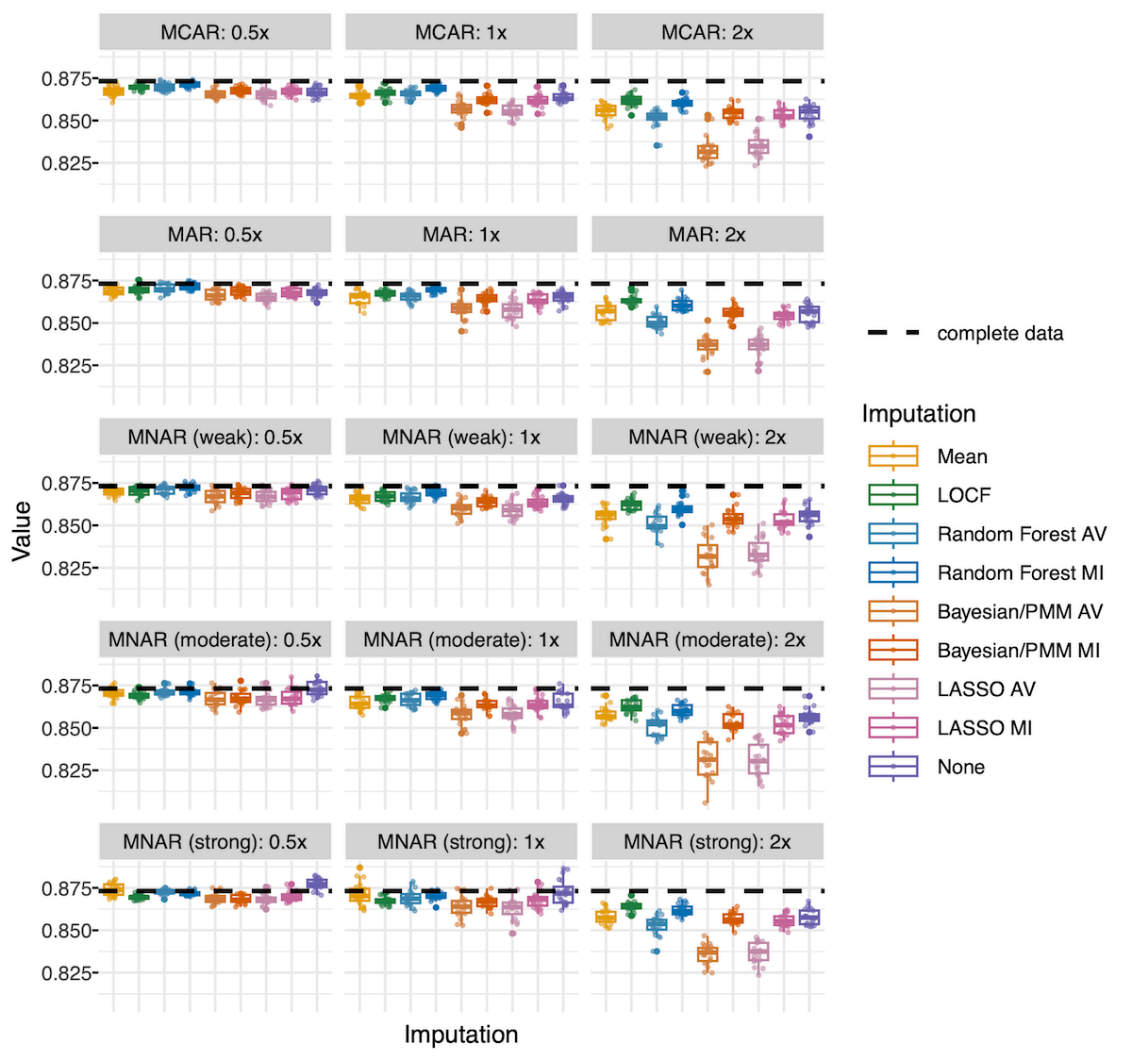
Gradient boosted model test performance (extubation): AUC. Each point represents AUC for 1 dataset. There are 20 datasets per missingness scenario and imputation type represented in each box plot. AUC in the complete dataset is represented by a dashed line. Other performance outcome metrics (sensitivity, specificity, positive predictive value [PPV], negative predictive value [NPV], and F1) are presented in Multimedia Appendix 9. AV, average (average of 30 imputations); Bayesian/PMM: Bayesian imputation under the normal linear model with predictive mean matching; LASSO: least absolute shrinkage and selection operator; LOCF: last observation carried forward; MAR, missing at random; MCAR: missing completely at random; MI: multiple imputation; MNAR: missing not at random.

Random forest multiple imputation had the highest AUC for gradient boosted models for 0.5x and 1x missingness, while LOCF did for 2x missingness (Figure 5). Random forest averaged, mean imputation, and no imputation had reasonable performance but degraded at 2x missingness. The amount of missingness continued to have a greater effect on performance than the missingness mechanism.

##### Blood Pressure: MSE

**Figure 6.**
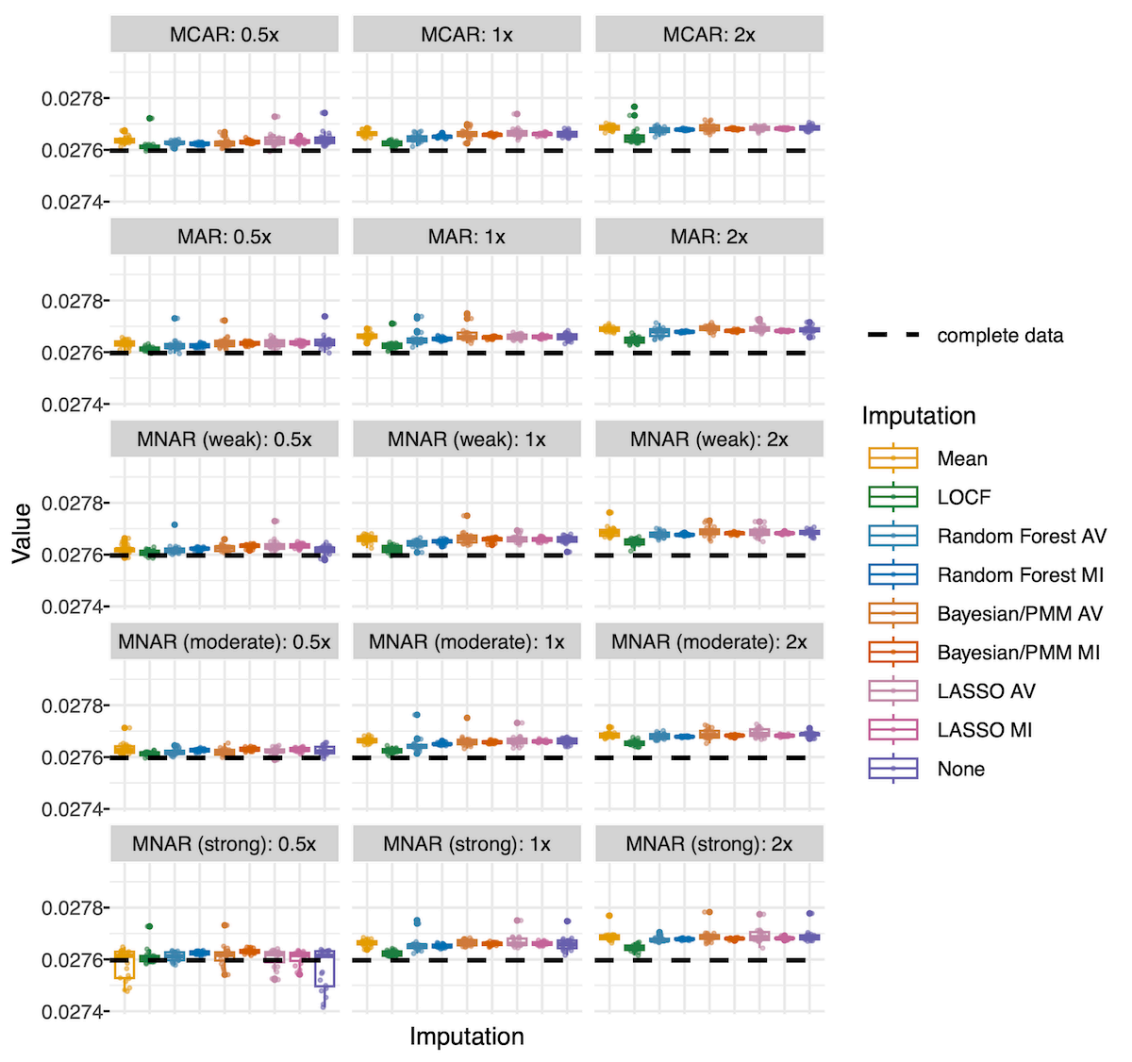
Gradient boosted model test performance (blood pressure): mean squared error (MSE). Each point represents MSE for 1 dataset. There are 20 datasets per missingness scenario and imputation type represented in each box plot. MSE in the complete dataset is represented by a dashed line. Other performance outcome metrics (mean absolute error [MAE], root MSE, and *R*^2^) are presented in Multimedia Appendix 10. AV, average (average of 30 imputations); Bayesian/PMM: Bayesian imputation under the normal linear model with predictive mean matching; LASSO: least absolute shrinkage and selection operator; LOCF: last observation carried forward; MAR, missing at random; MCAR: missing completely at random; MI: multiple imputation; MNAR: missing not at random.

LOCF had the lowest overall MSE (Figure 6). Random forest (both averaged and multiple imputation) generally had the next lowest MSE. Unlike in extubation models, mean imputation and no imputation did not perform substantially better than other methods. Performance was again more sensitive to amount of missingness than to missingness mechanism; overall, MSE increased in a stepwise fashion as missingness increased. However, there was less overall variability (CV=0.001) between models than for balanced accuracy (CV=0.042) and AUC (CV=0.012).

### LASSO Models

#### Extubation: Balanced Accuracy

For the LASSO outcome model, performance and patterns were similar to the gradient boosted model for balanced accuracy, with LOCF demonstrating top performance (Table S7 in Multimedia Appendix 1; Multimedia Appendix 11). Mean imputation did not perform as well in LASSO models. Interestingly, imputation methods that performed worst—Bayesian/PMM and LASSO—yielded better results in LASSO models than in gradient boosted models, with less variation in performance across methods (CV=0.029).

#### Extubation: AUC

AUC followed similar patterns to balanced accuracy for LASSO. LOCF had the best performance. Mean imputation again performed worse comparatively, and there was a smaller performance gap between the best and worst imputation methods relative to gradient boosted models (CV=0.011; Table S7 in Multimedia Appendix 1; Multimedia Appendix 11).

#### Blood Pressure: MSE

LOCF had the lowest overall MSE (Multimedia Appendix 12). MSE again increased as missingness increased. MSE of LASSO models for all imputation methods was higher than gradient boosted models, although LASSO MSE was consistently closer to the MSE for complete data. The range between the best and worst imputation methods was narrower than for gradient boosted models (CV=0.0008).

### Overfitting to training data: Both models

#### Extubation: Balanced Accuracy and AUC

Overfitting to the training data (MultimediaAppendices 13  14) increased as proportion of missingness increased. It was greatest for the worst-performing imputation methods (Bayesian/PMM and LASSO) in both gradient-boosted and LASSO outcome models. It was higher overall for gradient boosted models than for LASSO outcome models.

#### Blood Pressure: MSE

Gradient boosted models exhibited more overfitting overall and higher variability in overfitting than LASSO models (MultimediaAppendices 15  16).

## Discussion

### Overview

EHR data present challenges in how to handle missing data when developing clinical prediction models. It is critical to use methods that are transferable to new data when generating real-time predictions for use cases like clinical decision support tools. In a realistic EHR dataset, we compared imputation methods based on (1) imputation accuracy and (2) outcome prediction.

### Imputation Performance

LOCF and random forest multiple imputation consistently had the lowest MSE and classification error. LOCF has performed well in health survey and cohort datasets [18,20]. In our data, per-variable LOCF imputation error decreased monotonically with first-order autocorrelation, indicating better performance for more temporally stable variables. Random forest, while not explicitly designed for longitudinal data, was informed by lagged variables in our implementation and therefore also showed some gains for more temporally stable variables, though the association was weaker than for LOCF. By contrast, mean imputation does not account for correlation across repeated measures, and accordingly showed no relationship between autocorrelation and error. In the PICU, where many measurements are recorded frequently and less frequent measurements often indicate presumed stability over time, LOCF may be particularly suitable, while random forest may provide added value when temporal patterns are weaker.

As expected, imputation performance degraded as the proportion of missingness increased. Jäger et al [13] also found imputation performance generally worsened when difficulty (eg, higher missingness fraction and MNAR) increased. We found that missingness proportion affected imputation quality more than missingness mechanism. Although the imputation methods we employed are only theoretically valid in MCAR and MAR settings, performance in MNAR data was fairly similar, possibly due to the availability of repeated measurements over time, which may have captured underlying patterns. This is fortunate, given that EHR data are often likely MNAR [9].

### Prediction Model Performance

Many studies do not report imputation performance [9,15,16], focusing instead on prediction performance. In our experiment, LOCF generally yielded the highest prediction performance across outcome types and models. We evaluated whether LOCF’s strong performance was influenced by how we constructed the synthetic, complete dataset. Although it may have been somewhat inflated, sensitivity analyses confirmed that LOCF remained a top performer. Its accuracy was greatest for variables with higher first-order autocorrelation, which may help explain its strong performance in this setting. While criticized in inferential statistics for causing bias and low standard errors [6,40], here it outperformed many multiple imputation methods.

Random forest multiple imputation was the best performing multiple imputation model. Jaeger et al [27] also found it led to the best predictive performance across 12 imputation strategies with 2 different outcome models in a registry dataset. Perez-Lebel et al [9] attributed multiple imputation’s improved performance to ensembling (averaging multiple predictors) rather than accurately capturing the distribution of the missing values (the theoretical basis for its use in inferential statistics).

Native support for missing values (no imputation) yielded high balanced accuracy for gradient boosted models and reasonable performance for other metrics. Perez-Lebel et al [9] concluded that it had the best predictive performance in real-world EHR data, with the lowest computational cost. LOCF (which they did not test) also has very little computational cost but may be less broadly applicable.

As with imputation performance, missingness mechanism had less impact on prediction performance than proportion of missingness. Performance degraded substantially as missingness fraction increased, consistent with prior findings [13]. Interestingly, performance was similar across MAR, MCAR, and MNAR scenarios. Some studies suggest explicitly adding indicator variables for missingness to outcome models improves prediction in MNAR settings [9,10,41]. However, others argue against them because they are fragile to operational and practice changes and may not generalize well to other settings [11,12,42]. We therefore did not include such indicators, and performance in MNAR scenarios was comparable to MAR and MCAR scenarios. Machine learning models with native support for missing data may exploit missingness patterns for prediction. If no imputation is employed, missingness patterns should be closely monitored for drift and its effect on predictive performance [42].

Variability between imputation methods differed by model type and performance metric. LASSO outcome models showed less variability than gradient boosted models. In our experiment, imputation method appeared to have a greater impact on the binary outcome than the continuous outcome. Balanced accuracy had the highest variability between methods, followed by AUC; MSE exhibited substantially less variability than either. All imputation methods had lower MSE for gradient boosted models than LASSO models, indicating choice of outcome model mattered more than choice of imputation method.

### Relationship Between Imputation and Prediction Performance

The best performing methods for imputation—LOCF and random forest multiple imputation—also performed well in prediction. Thus, our main results for imputation and prediction performance were largely concordant. Mean imputation had the worst MSE but performed relatively well in gradient boosted models, possibly because constant imputation creates patterns that machine learning can exploit [9,43]. Its performance dropped in LASSO models. Others have noted that more accurate imputation methods do not always yield better predictions [9,27], especially when features are weakly correlated [9].

Overfitting in imputation models reduced imputation accuracy, which subsequently impacted the accuracy of outcome models. Outcome metrics that overfit more (eg, balanced accuracy in gradient boosted models) also showed greater variability between imputation methods in patterns that reflected differences in imputation accuracy.

### Imputation and Interpretability

For clinical decision support, it is important to assess how missing data handling affects interpretability. The full promise of artificial intelligence will not be realized if it is not deemed trustworthy and transparent by humans [44,45]. Mean imputation, the most common method of imputation in machine learning clinical prediction models [12], may reduce interpretability for clinicians trying to understand predictions for individual patients, as the mean is not meaningful at the individual level. Native support by algorithms may improve interpretability by relying only on recorded values. LOCF is simple to implement and aligns with clinical reasoning—if a measurement is expected to change and that change is important, it will be remeasured if possible. Finally, if complex methods like random forests (while less interpretable themselves) generate accurate imputations that reflect a biological or clinical relationship between the predictor and outcome, this could lead to more interpretable outcome models that are also more robust over time and across populations [9].

### Limitations

The primary limitation of this experiment is its reliance on both a single dataset and a single method for generating the complete dataset on which the analysis is based. However, a key strength is the high fidelity of the data, with detailed, frequent measurements typical of the intensive care unit. This contrasts with EHR data from settings like primary care, where visits may be months or years apart. Although we predicted both a binary and continuous outcome, our dataset was limited to a single PICU at one academic medical center. Raw data were transformed into a structured format with time windows and summary variables, a common approach for EHR data [2,46]. Thus, our findings may not generalize to other settings or data structures.

We restricted imputation methods in our experiment to those with readily available packages in R that allowed model training on 1 dataset and subsequent imputation in new data. Deep learning methods were not included, nor were novel approaches [47-49] that may outperform tested methods but are more complex to implement for applied practitioners. Performance could also theoretically be improved by combining imputation approaches (eg, a SuperLearner [50]).

Most existing imputation packages do not allow users to save model parameters to apply on new data, limiting the methods available [15]. Some have proposed workarounds, such as stacking data from a new patient with all training data and rerunning multiple imputation models [15,17]. However, this was computationally infeasible in a dataset of our size. Privacy concerns may also prevent access to training data in deployment. Even packages like *mice* that allow imputation on single new cases have limitations—each call refits an iteration of the model, making real-time imputation for new patients infeasible due to speed. The lack of scalable imputation tools remains a barrier to progress to deploying real-time clinical prediction models.

### Conclusion

When using EHR data with frequent measurements to build a prediction model, LOCF offers reasonable performance with simple implementation. Native support for missing data in machine learning models, such as gradient boosted trees, is the least computationally intensive approach, with decent performance and potentially broader applicability than LOCF. While multiple imputation is the gold standard for inferential models, it is extremely computationally intensive, may not be optimal for prediction models, and may not be feasible in real time. As clinical prediction models continue to integrate into real-time patient care, addressing missing data appropriately remains essential.

## Supplementary material

10.2196/79307 Multimedia Appendix 1 Supplementary methods and tables.

10.2196/79307 Multimedia Appendix 2 Imputation test performance metrics (blood pressure): mean squared error. Each point represents mean squared error calculated for 176 numeric variables for 1 of 300 datasets created for the outcome of blood pressure. There are 20 datasets per missingness scenario and imputation type represented in each box plot. AV: average (average of 30 imputations); Bayesian/PMM: Bayesian imputation under the normal linear model with predictive mean matching; LASSO: least absolute shrinkage and selection operator; LOCF: last observation carried forward.

10.2196/79307 Multimedia Appendix 3 Imputation test performance metrics (extubation and blood pressure): classification error. Each point represents classification error calculated for 6 categorical variables for 1 dataset (of 300 created for the outcome of extubation and 300 created for the outcome of blood pressure). There are 20 datasets per missingness scenario and imputation type represented in each box plot. The mice implementation of LASSO cannot accommodate multiclass categorical outcomes; thus, we used a simple classification tree. AV: average (average of 30 imputations); Bayesian/PMM: Bayesian imputation under the normal linear model with predictive mean matching; LASSO: least absolute shrinkage and selection operator; LOCF: last observation carried forward.

10.2196/79307 Multimedia Appendix 4 Imputation performance difference between train and test (extubation and blood pressure): mean squared error. Each point represents the difference in mean squared error between training and test sets calculated for 176 numeric variables for 1 dataset (of 300 created for the outcome of extubation and 300 created for the outcome of blood pressure). There are 20 datasets per missingness scenario and imputation type represented in each box plot. AV: average (average of 30 imputations); Bayesian/PMM: Bayesian imputation under the normal linear model with predictive mean matching; LASSO: least absolute shrinkage and selection operator; LOCF: last observation carried forward.

10.2196/79307 Multimedia Appendix 5 Imputation performance difference between train and test (extubation and blood pressure): classification error. Each point represents the difference in mean squared error between training and test sets calculated for 6 categorical variables for 1 dataset (of 300 created for the outcome of extubation and 300 created for the outcome of blood pressure). There are 20 datasets per missingness scenario and imputation type represented in each box plot. The mice implementation of LASSO cannot accommodate multiclass categorical outcomes; thus, we used a simple classification tree. AV: average (average of 30 imputations); Bayesian/PMM: Bayesian imputation under the normal linear model with predictive mean matching; LASSO: least absolute shrinkage and selection operator; LOCF: last observation carried forward.

10.2196/79307 Multimedia Appendix 6 Autocorrelation versus imputation error by method (extubation). Scatterplot of per-variable temporal autocorrelation (AR(1) coefficient, *x*-axis) versus imputation error (*y*-axis; mean squared error (MSE) for numeric variables, classification error for categorical) using 4-hour windows. Each point represents 1 predictor; higher persistence corresponds to lower error for last observation carried forward (LOCF).

10.2196/79307 Multimedia Appendix 7 Imputation test performance metrics by missingness in original data (extubation and blood pressure): mean squared error. Each point represents mean squared error calculated for 176 numeric variables for 1 dataset stratified by whether the value was missing in the original data (of 300 created for the outcome of extubation and 300 created for the outcome of blood pressure). There are 20 datasets per missingness scenario and imputation type represented in each box plot. AV: average (average of 30 imputations); Bayesian/PMM: Bayesian imputation under the normal linear model with predictive mean matching; LASSO: least absolute shrinkage and selection operator; LOCF: last observation carried forward.

10.2196/79307 Multimedia Appendix 8 Marginal means for interaction between imputation method, proportion missing, and variable group (extubation and blood pressure). We calculated marginal means for interaction from linear models of mean squared error comparing imputed values to complete dataset (for each outcome: 1 observation [n=264,000] per 176 variables per 300 datasets per 5 imputation methods). We calculated marginal means for interaction from linear models of classification error comparing imputed values to complete dataset (for each outcome: 1 observation [n=9000] per 6 variables per 300 datasets per 5 imputation methods) with random intercepts for each of the 1500 datasets. We included all 3-way and 2-way interactions and completed a backward stepwise elimination procedure (included *P*<.05) to determine the final model. Variable group 4, unlike other variable groups, included mostly indicator variables constructed from “select all that apply” responses in the electronic health record (EHR) (see Table S1). Bayesian/PMM: Bayesian imputation under the normal linear model with predictive mean matching; LASSO: least absolute shrinkage and selection operator; LOCF: last observation carried forward.

10.2196/79307 Multimedia Appendix 9 Gradient-boosted model test performance (extubation): other metrics. Each point represents performance for 1 dataset. There are 20 datasets per missingness scenario and imputation type represented in each box plot. Performance in the complete dataset is represented by a dashed line. AV: average (average of 30 imputations); Bayesian/PMM: Bayesian imputation under the normal linear model with predictive mean matching; LASSO: least absolute shrinkage and selection operator; LOCF: last observation carried forward; MI: multiple imputation; NPV: negative predictive value; PPV: positive predictive value.

10.2196/79307 Multimedia Appendix 10 Gradient boosted model test performance (blood pressure): other metrics. Each point represents performance for 1 dataset. There are 20 datasets per missingness scenario and imputation type represented in each box plot. Performance in the complete dataset is represented by a dashed line. AV: average (average of 30 imputations); Bayesian/PMM: Bayesian imputation under the normal linear model with predictive mean matching; LASSO: least absolute shrinkage and selection operator; LOCF: last observation carried forward; MAE: mean absolute error; MI: multiple imputation; NPV: negative predictive value; PPV: positive predictive value; RMSE: root mean squared error.

10.2196/79307 Multimedia Appendix 11 LASSO test performance (extubation): all metrics. Each point represents performance for 1 dataset. There are 20 datasets per missingness scenario and imputation type represented in each box plot. Performance in the complete dataset is represented by a dashed line. AV: average (average of 30 imputations); Bayesian/PMM: Bayesian imputation under the normal linear model with predictive mean matching; LASSO: least absolute shrinkage and selection operator; LOCF: last observation carried forward; MAE: mean absolute error; MI: multiple imputation; NPV: negative predictive value; PPV: positive predictive value; RMSE: root mean squared error.

10.2196/79307 Multimedia Appendix 12 LASSO test performance (blood pressure): all metrics. Each point represents performance for 1 dataset. There are 20 datasets per missingness scenario and imputation type represented in each box plot. Performance in the complete dataset is represented by a dashed line. AV: average (average of 30 imputations); Bayesian/PMM: Bayesian imputation under the normal linear model with predictive mean matching; LASSO: least absolute shrinkage and selection operator; LOCF: last observation carried forward; MAE: mean absolute error; MI: multiple imputation; MSE: mean squared error; NPV: negative predictive value; PPV: positive predictive value; RMSE: root mean squared error.

10.2196/79307 Multimedia Appendix 13 Gradient-boosted model difference between train and test (extubation): All performance metrics. Each point represents a difference in performance between train and test sets for 1 dataset. There are 20 datasets per missingness scenario and imputation type represented in each box plot. The difference in the complete dataset is represented by a dashed line. AV: average (average of 30 imputations); Bayesian/PMM: Bayesian imputation under the normal linear model with predictive mean matching; LASSO: least absolute shrinkage and selection operator; LOCF: last observation carried forward; MAE: mean absolute error; MI: multiple imputation; MSE: mean squared error; NPV: negative predictive value; PPV: positive predictive value; RMSE: root mean squared error.

10.2196/79307 Multimedia Appendix 14 LASSO model difference between train and test (extubation): all performance metrics. Each point represents a difference in performance between train and test sets for 1 dataset. There are 20 datasets per missingness scenario and imputation type represented in each box plot. The difference in the complete dataset is represented by a dashed line. AUC: area under the receiver operating characteristic curve; AV: average (average of 30 imputations); Bayesian/PMM: Bayesian imputation under the normal linear model with predictive mean matching; LASSO: least absolute shrinkage and selection operator; LOCF: last observation carried forward; MAE: mean absolute error; MI: multiple imputation; MSE: mean squared error; NPV: negative predictive value; PPV: positive predictive value; RMSE: root mean squared error.

10.2196/79307 Multimedia Appendix 15 Gradient boosted model difference between train and test (blood pressure): all performance metrics. Each point represents a difference in performance between train and test sets for 1 dataset. There are 20 datasets per missingness scenario and imputation type represented in each box plot. The difference in the complete dataset is represented by a dashed line. AV: average (average of 30 imputations); Bayesian/PMM: Bayesian imputation under the normal linear model with predictive mean matching; LASSO: least absolute shrinkage and selection operator; LOCF: last observation carried forward; MAE: mean absolute error; MI: multiple imputation; MSE: mean squared error; NPV: negative predictive value; PPV: positive predictive value; RMSE: root mean squared error.

10.2196/79307 Multimedia Appendix 16 LASSO model difference between train and test (blood pressure): all performance metrics. Each point represents a difference in performance between train and test sets for 1 dataset. There are 20 datasets per missingness scenario and imputation type represented in each box plot. The difference in the complete dataset is represented by a dashed line. AV: average (average of 30 imputations); Bayesian/PMM: Bayesian imputation under the normal linear model with predictive mean matching; LASSO: least absolute shrinkage and selection operator; LOCF: last observation carried forward; MAE: mean absolute error; MI: multiple imputation; MSE: mean squared error; NPV: negative predictive value; PPV: positive predictive value; RMSE: root mean squared error.

## Abbreviations

AUC: area under the receiver operating characteristic curve
CV: coefficient of variation
EHR: electronic health record
ETT: endotracheal tube
LASSO: least absolute shrinkage and selection operator
LOCF: last observation carried forward
MAR: missing at random
MCAR: missing completely at random
MI: multiple imputation
MNAR: missing not at random
MSE: mean squared error
PICU: pediatric intensive care unit
PMM: predictive mean matching
