# Supplementary material for: Methods for Addressing Missingness in Electronic Health Record Data for Clinical Prediction Models: Comparative Evaluation
Source: JMIR Med Inform. 2025 Nov 14;13:e79307. doi: 10.2196/79307 (PMC12617989; doi:10.2196/79307)
Supplement: Multimedia Appendix 1 [file medinform-v13-e79307-s001.pdf]

Supplementary Methods

Study population: Patients with tracheostomies at time of admission were excluded. Data from patients who died while intubated, died within 48 hours of extubation, or were extubated with the intent of withdrawal of life-sustaining support were included with the exception of 24 hours prior to extubation or death (whichever came first).

Outcome definition: Although most patients are ultimately successfully extubated, due to our data structure, successful extubation was a rare outcome (<2% of time windows). Most patients contributed multiple time windows where they remained intubated (were not successfully extubated according to our binary outcome) and one final time window where they were successfully extubated. Thus, our dataset had a large class imbalance.

Extubation could have been planned or unplanned. If a patient was reintubated within 48 hours solely for a procedure and then extubated again (intubated <12 hours), they were counted as successfully extubated.

The outcome for each time window reflects the patient's status 12 hours after the end of that window, calculated as the status after three subsequent 4-hour windows. However, because the start of each 4-hour window is defined by counting forward from the time of intubation, the final window may contain less than four hours if extubation does not coincide with multiples of four hours post-intubation (e.g., 24, 28, 32 hours). For instance, consider a patient intubated at 17:27 on 5/6/2018 and extubated at 08:45 on 5/8/2018 (Figure 1). The last time window included in the model for this patient is from 17:27 to 21:27 on 5/7/2018. The subsequent windows, not included

in the model, run from 5/7/2018 21:27 to 5/8/2018 01:27, from 01:27 to 05:27 on 5/8/2018, and from 05:27 to 08:45 on 5/8/2018. Since extubation occurred at the end of the third window, which was less than 12 hours after the end of the last included window, a more precise definition of the outcome is that it conveys the status 8.01 to 12 hours after the end of the last included time window.

Missingness in original data: Note that some variables had no missingness based on the way in which they were constructed. For example, if a medication was not recorded in the medication administration record during a given time window, a patient was assumed not to have received it.

Train/test split: We ensured that all time windows from each endotracheal tube (from intubation to extubation) were included only in the training data or the test data. When resampling the training data for tuning of outcome prediction model hyperparameters, we ensured that all observations from each endotracheal tube encounter were kept within the same fold.

Inducing missingness: Using the ampute command in the mice package (1), we induced missingness in 20 unique datasets for each of the 15 scenarios as follows. We divided variables into five groups (Supplementary Table 1) based on theoretical grounds and then modified groupings slightly based on the results of an empirical clustering analysis: 1) always observed variables (variables charted hourly such as vital signs, medications, fluid intake and output, as well as the outcome); 2) ventilator parameters and measures (charted a few times per shift); 3) blood gases (recorded with varying frequency); 4) clinical respiratory assessment (charted a few times per shift); 5) other clinical assessment parameters (tended to be least frequently charted variables). Patterns of missing data were created using these groups, such that each group of variables at a given time point was either completely missing or completely observed. This enabled creation of a manageable

number of missing data patterns, while also being realistic in that EHR variables are often charted at the same timepoint by a single person (e.g. nurse, respiratory therapist, or laboratory technician) and may or may not be observed together in a given time bin.

The missing data patterns were as follows (with the specified variable groups missing and all other variable groups observed):

1. Missing group 2
2. Missing group 3
3. Missing group 4
4. Missing group 5
5. Missing groups 2 and 3
6. Missing groups 2 and 4
7. Missing groups 2 and 5
8. Missing groups 3 and 4
9. Missing groups 3 and 5
10. Missing groups 4 and 5
11. Missing groups 2, 3, and 4
12. Missing groups 2, 3, and 5
13. Missing groups 2, 4, and 5
14. Missing groups 3, 4 and 5
15. Missing groups 2, 3, 4, and 5

The ampute command randomly divided each dataset into 15 subsets (one per missingness pattern), with frequencies of each pattern varying based on whether the dataset had 0.5x, 1x, or 2x the

percentage of cells missing compared with the original data. Then, within each subset, a specified proportion of rows (again, varying based on whether the dataset had 0.5x, 1x, or 2x missingness compared with the original data) are made incomplete according to the missingness mechanism specified. For the MCAR mechanism, missingness of groups 2-5 did not depend on any other variables and missingness within each subset was randomly induced. Weights dictated which variables influenced missingness in a given missingness pattern for the MAR and MNAR mechanisms. The outcome was allowed to influence missingness in all MAR and MNAR scenarios. (Note: we compared the results when the outcome was unrelated to missingness in MNAR scenarios and the conclusions did not change; results not included here.) Where n_obs = number of observed variables for a given missingness pattern, n_miss = number of missing variables, and y = the number of variables derived from a raw variable (e.g. some multiple select variables were encoded as multiple dummy variables), weights were constructed as follows:

- MAR: observed variables = 1/n_obs/y (including the outcome); missing variables = 0
- weak MNAR: observed variables = 1/n_obs/y; missing variables = 0.5/n_miss/y (including the outcome)
- moderate MNAR: observed variables = 1/n_obs/y; missing variables = 1/n_miss/y (including the outcome)
- strong MNAR: observed variables = 0; missing variables = 1/n_miss/y (including the outcome)

These weights were used to create weighted sum scores where the coefficients for a linear regression equation were the values of the weights matrix. Each candidate is assigned a probability of being missing for a given variable based on their weighted sum score. This probability is determined using one of four logistic distribution functions such that those with high, low, average, or extreme

weighted sum scores have a higher probability of being missing.(1) Each dataset was randomly assigned to one of this distributions.

We added lagged variables to each observation after inducing missingness so that variables missing in one time window were also missing as lagged variables in other time windows (as they would be in real data).

Methods to handle missingness:

1) Mean
2) Last observation carried forward (LOCF): While many have argued against its use(2), it largely reflects how clinicians view many measures in practice. For example, if labs are not measured, it is generally because clinicians assume levels are not meaningfully different than the prior value. If clinicians suspect important changes have taken place, lab values are measured again. Here, we carried values forward indefinitely. Approximately 1% of cells remained missing because no prior value was observed; we filled these with mean (numeric and binary) or mode (factor) values from the training set.
3) Random forest: For each variable with missing values, the mice package(3) (mice function, method = "rf") builds a decision tree, predicts the terminal node for each observation, and randomly samples an observed donor value that shares a terminal node with a missing value. A forest of these trees is built and one tree's donor value is randomly selected to be the final imputed value for each missing value.
4) Bayesian imputation under the normal linear model with predictive mean matching (Bayesian/PMM): The mice package (mice function, method = "pmm") uses Bayesian imputation under the normal linear model to determine the matches. A pool of five donors

is defined by a distance calculated between predicted values for the observed data and values calculated from a random draw from the posterior distribution of coefficients for the missing data.

5) Least absolute shrinkage and selection operator (LASSO): Mice implements LASSO (mice function, methods = "lasso.norm" and "lasso.logreg") for each variable by drawing a bootstrap sample with replacement from observed cases and fitting a regularized linear regression. Mice then draws imputed values from the distribution defined by the original (non-bootstrap) data, the estimated regression coefficients, and estimated error variance. The mice implementation of LASSO cannot accommodate multi-class categorical outcomes; thus, for categorical variables (n=6) we used a simple classification tree (mice function, method = "cart").
6) Native support for missing data in prediction model (no imputation required): The LightGBM package(4) allocates missing values to the bins that optimally minimize loss.

For methods 3-5, the imputation models included all variables in the dataset, excluding the outcome. Models were built with the mice package, which uses fully conditional specification to generate multiple imputations for incomplete multivariate data by Gibbs sampling.

Hyperparameter tuning for outcome prediction models:

We employed 5-fold cross-validation on the training set to tune hyperparameters, ensuring that all time windows associated with each intubation were grouped together within the same fold.

To tune the gradient boosted trees models, we did a grid search of the following hyperparameters:

- Number of leaves: 2, 4, 8

- Learning rate: 0.001, 0.01, 0.1

Early stopping was implemented if there was no improvement in performance after 10 rounds.

To tune the LASSO models, the function cv.glmnet was used to automatically tune lambda and determine the largest value of lambda such that the cross-validated error is within one standard error of the minimum error (lambda.1se).

Analysis of Imputation Accuracy

We initially constructed multilevel models with random intercepts for each dataset but encountered issues with singularity. Given that the results from models without random intercepts were virtually identical, we proceeded with the simpler approach.

Association between Temporal Autocorrelation and Imputation Performance

To evaluate whether temporal autocorrelation was associated with imputation, we estimated first-order autocorrelation for each variable in the synthetic complete dataset. For numeric and ordinal variables, we fit generalized least squares models with an AR(1) correlation structure within patient intubation encounters, yielding per-variable lag-1 autocorrelation coefficients. For the single unordered categorical variable, we calculated lag-1 Cramér's V by cross-tabulating consecutive values within intubation encounters. Imputation error, expressed as mean squared error for numeric and ordinal variables or classification error for the unordered categorical variable, was computed for each variable as the mean across all 300 datasets. Lagged variables were excluded from this analysis but were included as predictors in the imputation models. We then examined the association between autocorrelation and imputation error descriptively using scatterplots and numerically by calculating Pearson correlation coefficients between the two metrics for each imputation method.

Analysis of Outcome Model Performance

We built a linear model for the outcomes of balanced accuracy and AUC for each outcome model type (GBM and LASSO) including as covariates: imputation type, missingness type, and proportion missing data (0.5x, 1x, 2x original). We included all three-way and two-way interactions and again used backward stepwise elimination. See Supplementary Table 6 for results.

Code

Code is available at: https://github.com/jeandigitale/MissingData.

References

1. Schouten RM, Lugtig P, Vink G. Generating missing values for simulation purposes: a multivariate amputation procedure. J Stat Comput Simul. 2018 Oct 13;88(15):2909–30.

2. Lachin JM. Fallacies of Last Observation Carried Forward. Clin Trials Lond Engl. 2016 Apr;13(2):161–8.

3. Buuren SV, Groothuis-Oudshoorn K. **mice** : Multivariate Imputation by Chained Equations in *R*. J Stat Softw [Internet]. 2011 [cited 2023 Oct 16];45(3). Available from: http://www.jstatsoft.org/v45/i03/

4. Shi Y, Ke G, Soukhavong D, Lamb J, Meng Q, Finley T, et al. lightgbm: Light Gradient Boosting Machine [Internet]. 2023. Available from: https://CRAN.R-project.org/package=lightgbm

Supplementary Table 1. Variable groups

Variable	Initial theoretical group	Empirical cluster	Final group	Final group name
Age at time of intubation (in days)	1	5	1	always observed
Alternative gases	2	5	1	always observed
Any arterial blood pressures in time window	1	5	1	always observed
ETT has a cuff	2	5	1	always observed
Diastolic blood pressure	1	5	1	always observed
Diastolic blood pressure percentile (age-adjusted)	1	5	1	always observed
Diagnosis of airway obstruction	1	5	1	always observed
Diagnosis of cardiomyopathy	1	5	1	always observed
Diagnosis of critical airway	1	5	1	always observed
Diagnosis of neuromuscular weakness	1	5	1	always observed
End tidal CO2 (mmHg)	2	5	1	always observed
ETT cuff leak pressure (not measured, <30, >=30)	1	5	1	always observed
ETT placed day of surgery	2	5	1	always observed
ETT size	2	5	1	always observed
Extubation success lagged 12 hours (outcome variable)	1	5	1	always observed
FiO2	1	5	1	always observed
Hours intubated	1	5	1	always observed
Intake/output total mL over the prior 12 hours/kg	1	5	1	always observed
Intake/output total mL over the prior 72 hours/kg	1	5	1	always observed
Mean arterial pressure (mmHg)	1	5	1	always observed
Medication: received airway dosing of dexamethasone	1	5	1	always observed
Medication: received any diuretic	1	5	1	always observed
Medication: received any paralytic infusion	1	5	1	always observed
Medication: number of vasopressors received	1	5	1	always observed
Medication: total midazolam equivalents (mg/kg)	1	5	1	always observed
Medication: total oral morphine equivalents (mg/kg)	1	5	1	always observed

Variable	Initial theoretical group	Empirical cluster	Final group	Final group name
Medication: received any other sedation	1	5	1	always observed
ETT present on hospital admission	2	5	1	always observed
Pulse	1	5	1	always observed
Respiratory rate (recorded in vital signs)	1	5	1	always observed
Spontaneous breathing test [Categories: passed, failed, eligible but inconclusive, ineligible, not assessed]	2	5	1	always observed
Sex	1	5	1	always observed
SpO2	1	5	1	always observed
Systolic blood pressure	1	5	1	always observed
Systolic blood pressure percentile (age-adjusted)	1	5	1	always observed
Temperature	1	5	1	always observed
Weight change since intubation	4	5	1	always observed
Weight-for-age z-score at intubation	1	5	1	always observed
Exhaled tidal volume/kg	2	3	2	ventilator parameters
Mean airway pressure (measured)	2	3	2	ventilator parameters
PEEP	2	3	2	ventilator parameters
Peak inspiratory pressure (measured)	2	3	2	ventilator parameters
Pressure support	2	3	2	ventilator parameters
Respiratory rate (set on ventilator)	2	3	2	ventilator parameters
Total respiratory rate (measured on ventilator)	2	3	2	ventilator parameters
Ventilator mode is APRV or HFOV	2	3	2	ventilator parameters
Base excess	3	1	3	blood gases
Hemoglobin	3	1	3	blood gases
Lactate	3	1	3	blood gases

Variable	Initial theoretical group	Empirical cluster	Final group	Final group name
Oxygenation index/oxygen saturation index [Categories: normal per OI, mild PARDS per OI, moderate PARDS per OI, severe PARDS per OI, normal per OSI, mild PARDS per OSI, moderate PARDS per OSI, severe PARDS per OSI]	2	1	3	blood gases
pCO2	3	1	3	blood gases
pH	3	1	3	blood gases
Sample type of blood gas is arterial	3	1	3	blood gases
Breath sounds: absent	5	2	4	respiratory assessment
Breath sounds: clear	5	2	4	respiratory assessment
Breath sounds: coarse	5	2	4	respiratory assessment
Breath sounds: crackles	5	2	4	respiratory assessment
Breath sounds: diminished	5	2	4	respiratory assessment
Breath sounds: expiratory wheezes	5	2	4	respiratory assessment
Breath sounds: inspiratory wheezes	5	2	4	respiratory assessment
Breath sounds: other	5	2	4	respiratory assessment
Breath sounds: rhonchi/rales	5	2	4	respiratory assessment
Breath sounds: unable to assess	5	2	4	respiratory assessment
Cough: none	4	4	4	respiratory assessment
Cough: present	4	4	4	respiratory assessment
Cough: unable to assess	4	4	4	respiratory assessment
Cough: weak	4	4	4	respiratory assessment
Respiratory pattern: accessory muscle use (mild)	5	2	4	respiratory assessment
Respiratory pattern: accessory muscle use (moderate/severe)	5	2	4	respiratory assessment
Respiratory pattern: irregular	5	2	4	respiratory assessment
Respiratory pattern: labored	5	2	4	respiratory assessment
Respiratory pattern: nasal flaring	5	2	4	respiratory assessment
Respiratory pattern: other	5	2	4	respiratory assessment

Variable	Initial theoretical group	Empirical cluster	Final group	Final group name
Respiratory pattern: regular	5	2	4	respiratory assessment
Respiratory pattern: retractions (mild)	5	2	4	respiratory assessment
Respiratory pattern: retractions (moderate/severe)	5	2	4	respiratory assessment
Respiratory pattern: shallow	5	2	4	respiratory assessment
Respiratory pattern: tachypneic	5	2	4	respiratory assessment
Respiratory pattern: unlabored	5	2	4	respiratory assessment
Secretion amount [Categories: none, scant, small, moderate, large, copious]	5	4	4	respiratory assessment
Secretion color: bloody	5	4	4	respiratory assessment
Secretion color: clear	5	4	4	respiratory assessment
Secretion color: other	5	4	4	respiratory assessment
Secretion color: pink-tinged	5	4	4	respiratory assessment
Secretion color: tan	5	4	4	respiratory assessment
Secretion color: white	5	4	4	respiratory assessment
Secretion color: yellow	5	4	4	respiratory assessment
Secretion consistency: frothy	5	4	4	respiratory assessment
Secretion consistency: mucous plugs	5	4	4	respiratory assessment
Secretion consistency: tenacious	5	4	4	respiratory assessment
Secretion consistency: thick	5	4	4	respiratory assessment
Secretion consistency: thin	5	4	4	respiratory assessment
Central venous pressure (mmHg)	4	1	5	other clinical parameters
Glasgow coma scale score	4	4	5	other clinical parameters
Height-for-age z-score at intubation	4	5	5	other clinical parameters
Lower extremity motor response	4	4	5	other clinical parameters
State behavioral scale	4	5	5	other clinical parameters
Upper extremity motor response	4	4	5	other clinical parameters
White blood cell count	4	1	5	other clinical parameters

Variable	Initial theoretical group	Empirical cluster	Final group	Final group name
Weight-for-length z-score/BMI z-score at intubation	4	5	5	other clinical parameters

Supplementary Table 2. Missingness in original data

Variable	Number of 4-hr time windows missing variable	Percent of 4-hr time windows missing variable	Group Number	Group Name
Age at time of intubation (in days)	0	0.0	1	always observed
Alternative gases	0	0.0	1	always observed
Diagnosis of airway obstruction	0	0.0	1	always observed
Diagnosis of cardiomyopathy	0	0.0	1	always observed
Diagnosis of critical airway	0	0.0	1	always observed
Diagnosis of neuromuscular weakness	0	0.0	1	always observed
ETT placed day of surgery	0	0.0	1	always observed
Hours intubated	0	0.0	1	always observed
Medication: received airway dosing of dexamethasone	0	0.0	1	always observed
Medication: received any diuretic	0	0.0	1	always observed
Medication: received any paralytic infusion	0	0.0	1	always observed
Medication: number of vasopressors received	0	0.0	1	always observed
Medication: total midazolam equivalents (mg/kg)	0	0.0	1	always observed
Medication: total oral morphine equivalents (mg/kg)	0	0.0	1	always observed
Medication: received any other sedation	0	0.0	1	always observed
Spontaneous breathing test [Categories: passed, failed, eligible but inconclusive, ineligible, not assessed]	0	0.0	1	always observed
Sex	0	0.0	1	always observed
Weight change since intubation	0	0.0	1	always observed
Weight-for-age z-score at intubation	0	0.0	1	always observed
Intake/output total mL over the prior 72 hours/kg	95	0.2	1	always observed

Variable	Number of 4-hr time windows missing variable	Percent of 4-hr time windows missing variable	Group Number	Group Name
Intake/output total mL over the prior 12 hours/kg	167	0.3	1	always observed
SpO2	404	0.7	1	always observed
Pulse	420	0.7	1	always observed
ETT cuff leak pressure (not measured, <30, >=30)	968	1.7	1	always observed
FiO2	1115	2.0	1	always observed
Respiratory rate (recorded in vital signs)	1179	2.1	1	always observed
Any arterial blood pressures in time window	1216	2.2	1	always observed
Diastolic blood pressure	1216	2.2	1	always observed
Systolic blood pressure	1216	2.2	1	always observed
Mean arterial pressure (mmHg)	1379	2.4	1	always observed
ETT present on hospital admission	2587	4.6	1	always observed
Temperature	2631	4.7	1	always observed
ETT has a cuff	4057	7.2	1	always observed
End tidal CO2 (mmHg)	4517	8.0	1	always observed
ETT size	6179	11.0	1	always observed
Diastolic blood pressure percentile (age-adjusted)	9827	17.5	1	always observed
Systolic blood pressure percentile (age-adjusted)	9827	17.5	1	always observed
Ventilator mode is APRV or HFOV	8973	15.9	2	ventilator parameters
PEEP	8975	15.9	2	ventilator parameters
Peak inspiratory pressure (measured)	9757	17.3	2	ventilator parameters
Mean airway pressure (measured)	10040	17.8	2	ventilator parameters
Exhaled tidal volume/kg	10274	18.3	2	ventilator parameters
Total respiratory rate (measured on ventilator)	10321	18.3	2	ventilator parameters

Variable	Number of 4-hr time windows missing variable	Percent of 4-hr time windows missing variable	Group Number	Group Name
Pressure support	11240	20.0	2	ventilator parameters
Respiratory rate (set on ventilator)	13165	23.4	2	ventilator parameters
Sample type of blood gas is arterial	25800	45.8	3	blood gas
pH	25804	45.8	3	blood gas
pCO2	25810	45.9	3	blood gas
Base excess	25889	46.0	3	blood gas
Hemoglobin	26401	46.9	3	blood gas
Lactate	27039	48.0	3	blood gas
Oxygenation index/oxygen saturation index [Categories: normal per OI, mild PARDS per OI, moderate PARDS per OI, severe PARDS per OI, normal per OSI, mild PARDS per OSI, moderate PARDS per OSI, severe PARDS per OSI]	30948	55.0	3	blood gas
Respiratory pattern: other	7194	12.8	4	respiratory assessment
Respiratory pattern: irregular	7290	13.0	4	respiratory assessment
Respiratory pattern: regular	7290	13.0	4	respiratory assessment
Respiratory pattern: shallow	7290	13.0	4	respiratory assessment
Respiratory pattern: tachypneic	7290	13.0	4	respiratory assessment
Breath sounds: diminished	7415	13.2	4	respiratory assessment
Breath sounds: clear	7419	13.2	4	respiratory assessment
Breath sounds: coarse	7420	13.2	4	respiratory assessment
Breath sounds: other	7420	13.2	4	respiratory assessment
Breath sounds: absent	7421	13.2	4	respiratory assessment
Breath sounds: crackles	7421	13.2	4	respiratory assessment
Breath sounds: expiratory wheezes	7421	13.2	4	respiratory assessment

Variable	Number of 4-hr time windows missing variable	Percent of 4-hr time windows missing variable	Group Number	Group Name
Breath sounds: inspiratory wheezes	7421	13.2	4	respiratory assessment
Breath sounds: rhonchi/rales	7421	13.2	4	respiratory assessment
Breath sounds: unable to assess	7421	13.2	4	respiratory assessment
Respiratory pattern: labored	7601	13.5	4	respiratory assessment
Respiratory pattern: nasal flaring	7601	13.5	4	respiratory assessment
Respiratory pattern: unlabored	7601	13.5	4	respiratory assessment
Respiratory pattern: retractions (mild)	9400	16.7	4	respiratory assessment
Respiratory pattern: retractions (moderate/severe)	9719	17.3	4	respiratory assessment
Respiratory pattern: accessory muscle use (mild)	9782	17.4	4	respiratory assessment
Respiratory pattern: accessory muscle use (moderate/severe)	9782	17.4	4	respiratory assessment
Secretion amount [Categories: none, scant, small, moderate, large, copious]	16325	29.0	4	respiratory assessment
Secretion color: bloody	17058	30.3	4	respiratory assessment
Secretion color: clear	17058	30.3	4	respiratory assessment
Secretion color: other	17058	30.3	4	respiratory assessment
Secretion color: pink-tinged	17058	30.3	4	respiratory assessment
Secretion color: tan	17058	30.3	4	respiratory assessment
Secretion color: white	17058	30.3	4	respiratory assessment
Secretion color: yellow	17058	30.3	4	respiratory assessment
Secretion consistency: frothy	17463	31.0	4	respiratory assessment
Secretion consistency: mucous plugs	17463	31.0	4	respiratory assessment
Secretion consistency: tenacious	17463	31.0	4	respiratory assessment
Secretion consistency: thick	17463	31.0	4	respiratory assessment

Variable	Number of 4-hr time windows missing variable	Percent of 4-hr time windows missing variable	Group Number	Group Name
Secretion consistency: thin	17463	31.0	4	respiratory assessment
Cough: none	35254	62.6	4	respiratory assessment
Cough: present	35254	62.6	4	respiratory assessment
Cough: unable to assess	35254	62.6	4	respiratory assessment
Cough: weak	35254	62.6	4	respiratory assessment
Height-for-age z-score at intubation	6980	12.4	5	other clinical parameters
Weight-for-length z-score/BMI z-score at intubation	7079	12.6	5	other clinical parameters
State behavioral scale	15888	28.2	5	other clinical parameters
Glasgow coma scale score	25751	45.7	5	other clinical parameters
Lower extremity motor response	28641	50.9	5	other clinical parameters
Upper extremity motor response	28839	51.2	5	other clinical parameters
Central venous pressure (mmHg)	30677	54.5	5	other clinical parameters
White blood cell count	43077	76.5	5	other clinical parameters

Note: N=56,287 4-hour time windows; these data include more time windows than the final analytic dataset because they were compiled before excluding time windows that were constructed solely for creating lagged variables.

Supplementary Table 3. Proportion of cells missing in simulated data compared with original data

Missingness mechanism	Missingness proportion in relation to original data (0.5x, 1x, 2x missingness)	Percent cells missing					
		Overall	Group 1	Group 2	Group 3	Group 4	Group 5
Original data	NA	18.2	2.7	17.3	47.0	24.0	41.1
**Outcome: Extubation**							
MCAR	0.5	9.6	0.0	10.0	24.0	14.0	22.0
MAR	0.5	9.5	0.0	9.9	23.9	13.9	21.9
MNAR (weak)	0.5	9.6	0.0	10.0	23.9	14.0	22.0
MNAR (moderate)	0.5	9.6	0.0	10.0	24.0	14.0	22.0
MNAR (strong)	0.5	9.6	0.0	9.9	24.0	14.0	22.0
MCAR	1	18.1	0.0	18.3	49.8	25.8	41.5
MAR	1	18.1	0.0	18.2	49.8	25.7	41.5
MNAR (weak)	1	18.1	0.0	18.2	49.8	25.7	41.5
MNAR (moderate)	1	18.1	0.0	18.3	49.8	25.7	41.6
MNAR (strong)	1	18.1	0.0	18.2	49.9	25.7	41.6
MCAR	2	35.9	0.0	39.8	81.4	53.4	81.5
MAR	2	35.9	0.0	39.8	81.5	53.4	81.5
MNAR (weak)	2	35.9	0.0	39.7	81.5	53.4	81.5
MNAR (moderate)	2	35.9	0.0	39.8	81.4	53.4	81.5
MNAR (strong)	2	35.9	0.0	39.8	81.5	53.4	81.4
**Outcome: Blood Pressure**							
MCAR	0.5	9.6	0.0	10.0	24.0	14.0	22.0
MAR	0.5	9.6	0.0	9.9	24.0	14.0	22.1
MNAR (weak)	0.5	9.6	0.0	10.0	23.9	13.9	22.1
MNAR (moderate)	0.5	9.6	0.0	10.0	24.0	14.0	21.9

MNAR (strong)	0.5	9.6	0.0	10.0	24.0	14.0	22.0
MCAR	1	18.1	0.0	18.3	49.8	25.7	41.5
MAR	1	18.1	0.0	18.3	49.7	25.7	41.5
MNAR (weak)	1	18.2	0.0	18.2	49.8	25.8	41.5
MNAR (moderate)	1	18.1	0.0	18.3	49.7	25.7	41.6
MNAR (strong)	1	18.1	0.0	18.3	49.8	25.7	41.5
MCAR	2	35.8	0.0	39.8	81.4	53.3	81.4
MAR	2	35.9	0.0	39.9	81.6	53.4	81.6
MNAR (weak)	2	35.9	0.0	39.8	81.5	53.4	81.5
MNAR (moderate)	2	35.9	0.0	39.8	81.5	53.4	81.5
MNAR (strong)	2	35.9	0.0	39.8	81.5	53.4	81.5

Note: For datasets with induced missingness, each row represents the average of 20 unique datasets.

Supplementary Table 4. Imputation performance: Mean squared error

	Extubation			Blood Pressure		
**Variable**	**Coefficient**	**Standard error**	**p-value**	**Coefficient**	**Standard error**	**p-value**
(Intercept)	0.161	0.008	<0.01	0.166	0.008	<0.01
**Imputation method**						
LOCF	Ref			Ref		
Mean	0.862	0.011	<0.01	0.845	0.011	<0.01
Random forest	0.225	0.011	<0.01	0.218	0.011	<0.01
Bayesian/PMM	0.110	0.011	<0.01	0.103	0.011	<0.01
LASSO	0.091	0.011	<0.01	0.084	0.011	<0.01
**Missingness mechanism**						
MCAR	Ref			Ref		
MAR	-0.006	0.004	0.15	0.009	0.004	0.03
MNAR (weak)	-0.008	0.004	0.05	-0.016	0.004	<0.01
MNAR (moderate)	0.023	0.004	<0.01	0.009	0.004	0.04
MNAR (strong)	0.009	0.004	0.02	-0.016	0.004	<0.01
**Proportion missing**						
0.5x	Ref			Ref		
1x	0.013	0.012	0.25	0.010	0.012	0.40
2x	0.043	0.012	<0.01	0.035	0.012	<0.01
**Variable group**						
2	Ref			Ref		
3	0.007	0.012	0.56	0.005	0.012	0.65
4	0.658	0.009	<0.01	0.657	0.009	<0.01
5	0.030	0.012	<0.01	0.028	0.012	0.02
**Imputation method * proportion missing**						
LOCF * 0.5x	Ref			Ref		
Mean * 1x	-0.026	0.016	0.10	-0.010	0.016	0.54
Random forest * 1x	0.022	0.016	0.15	0.027	0.016	0.08
Bayesian/PMM * 1x	0.030	0.016	0.06	0.036	0.016	0.02

	Extubation			Blood Pressure		
**Variable**	**Coefficient**	**Standard error**	**p-value**	**Coefficient**	**Standard error**	**p-value**
LASSO * 1x	0.025	0.016	0.11	0.032	0.016	0.04
Mean * 2x	-0.044	0.016	<0.01	-0.026	0.016	0.10
Random forest * 2x	0.114	0.016	<0.01	0.121	0.016	<0.01
Bayesian/PMM * 2x	0.164	0.016	<0.01	0.170	0.016	<0.01
LASSO * 2x	0.166	0.016	<0.01	0.174	0.016	<0.01
**Imputation method * variable group**						
LOCF * 2	Ref			Ref		
Mean * 3	-0.021	0.017	0.22	-0.011	0.017	0.53
Random forest * 3	-0.039	0.017	0.02	-0.035	0.017	0.04
Bayesian/PMM * 3	0.361	0.017	<0.01	0.363	0.017	<0.01
LASSO * 3	0.386	0.017	<0.01	0.387	0.017	<0.01
Mean * 4	-0.635	0.012	<0.01	-0.623	0.012	<0.01
Random forest * 4	-0.262	0.012	<0.01	-0.259	0.012	<0.01
Bayesian/PMM * 4	-0.090	0.012	<0.01	-0.085	0.012	<0.01
LASSO * 4	-0.091	0.012	<0.01	-0.087	0.012	<0.01
Mean * 5	0.003	0.017	0.84	0.012	0.017	0.47
Random forest * 5	-0.015	0.017	0.35	-0.013	0.017	0.45
Bayesian/PMM * 5	0.259	0.017	<0.01	0.262	0.017	<0.01
LASSO * 5	0.286	0.017	<0.01	0.285	0.017	<0.01
**Missingness mechanism * proportion missing**						
MCAR * 0.5x	Ref			Ref		
MAR * 1x	0.004	0.006	0.44	-0.015	0.006	0.01
MNAR (weak) * 1x	0.017	0.006	<0.01	0.016	0.006	<0.01
MNAR (moderate) * 1x	-0.026	0.006	<0.01	-0.011	0.006	0.06
MNAR (strong) * 1x	-0.020	0.006	<0.01	0.004	0.006	0.45
MAR * 2x	-0.003	0.006	0.62	-0.016	0.006	<0.01
MNAR (weak) * 2x	-0.003	0.006	0.66	0.015	0.006	0.01
MNAR (moderate) * 2x	-0.031	0.006	<0.01	-0.015	0.006	<0.01
MNAR (strong) * 2x	-0.020	0.006	<0.01	0.014	0.006	0.02

	Extubation			Blood Pressure		
**Variable**	**Coefficient**	**Standard error**	**p-value**	**Coefficient**	**Standard error**	**p-value**
**Proportion missing * variable group**						
0.5x * 2	Ref			Ref		
1x * 3	0.046	0.017	<0.01	0.045	0.017	<0.01
2x * 3	0.189	0.017	<0.01	0.188	0.017	<0.01
1x * 4	0.037	0.012	<0.01	0.038	0.012	<0.01
2x * 4	0.150	0.012	<0.01	0.150	0.012	<0.01
1x * 5	0.018	0.017	0.27	0.020	0.017	0.22
2x * 5	0.149	0.017	<0.01	0.148	0.017	<0.01
**Imputation method * proportion missing * variable group**						
LOCF * 0.5x * 2	Ref			Ref		
Mean * 1x * 3	-0.038	0.024	0.12	-0.045	0.024	0.06
Random forest * 1x * 3	0.077	0.024	<0.01	0.077	0.024	<0.01
Bayesian/PMM * 1x * 3	-0.001	0.024	0.97	0.000	0.024	1.00
LASSO * 1x * 3	0.021	0.024	0.39	0.022	0.024	0.37
Mean * 2x * 3	-0.183	0.024	<0.01	-0.193	0.024	<0.01
Random forest * 2x * 3	0.039	0.024	0.10	0.036	0.024	0.14
Bayesian/PMM * 2x * 3	-0.166	0.024	<0.01	-0.168	0.024	<0.01
LASSO * 2x * 3	-0.168	0.024	<0.01	-0.171	0.024	<0.01
Mean * 1x * 4	-0.023	0.017	0.18	-0.029	0.018	0.10
Random forest * 1x * 4	-0.038	0.017	0.03	-0.034	0.018	0.06
Bayesian/PMM * 1x * 4	-0.001	0.017	0.95	0.001	0.018	0.94
LASSO * 1x * 4	-0.035	0.017	0.05	-0.034	0.018	0.05
Mean * 2x * 4	-0.137	0.017	<0.01	-0.148	0.018	<0.01
Random forest * 2x * 4	-0.172	0.017	<0.01	-0.174	0.018	<0.01
Bayesian/PMM * 2x * 4	-0.090	0.017	<0.01	-0.095	0.018	<0.01
LASSO * 2x * 4	-0.207	0.017	<0.01	-0.210	0.018	<0.01
Mean * 1x * 5	-0.013	0.023	0.59	-0.022	0.024	0.35
Random forest * 1x * 5	0.047	0.023	0.04	0.044	0.024	0.06
Bayesian/PMM * 1x * 5	0.031	0.023	0.19	0.029	0.024	0.22

	Extubation			Blood Pressure		
**Variable**	**Coefficient**	**Standard error**	**p-value**	**Coefficient**	**Standard error**	**p-value**
LASSO * 1x * 5	0.049	0.023	0.04	0.049	0.024	0.04
Mean * 2x * 5	-0.146	0.023	<0.01	-0.154	0.024	<0.01
Random forest * 2x * 5	0.091	0.023	<0.01	0.089	0.024	<0.01
Bayesian/PMM * 2x * 5	-0.050	0.023	0.03	-0.050	0.024	0.03
LASSO * 2x * 5	-0.060	0.023	<0.01	-0.060	0.024	0.01

Note: Linear models of mean squared error comparing imputed values to complete dataset (each model had one observation [n=264,000] per 176 variables per 300 datasets per 5 imputation methods). We included all three-way and two-way interactions and completed a backward stepwise elimination procedure (included $p<0.05$) to determine the final model.

Abbreviations: LOCF = last observation carried forward; Bayesian/PMM = Bayesian imputation under the normal linear model with predictive mean matching; LASSO = Least Absolute Shrinkage and Selection Operator; MCAR = missing completely at random; MAR = missing at random; MNAR = missing not at random

Supplementary Table 5. Imputation performance: Classification error

	Extubation			Blood Pressure		
**Variable**	**Coefficient**	**Standard error**	**p-value**	**Coefficient**	**Standard error**	**p-value**
(Intercept)	0.167	0.001	<0.01	0.171	0.001	<0.01
**Imputation method**						
LOCF	Ref			Ref		
Mean	0.520	0.002	<0.01	0.519	0.002	<0.01
Random forest	0.010	0.002	<0.01	0.010	0.002	<0.01
Bayesian/PMM	0.196	0.002	<0.01	0.196	0.002	<0.01
LASSO	0.051	0.002	<0.01	0.054	0.002	<0.01
**Missingness mechanism**						
MCAR	Ref			Ref		
MAR	0.005	0.001	<0.01	0.002	0.001	<0.01
MNAR (weak)	0.007	0.001	<0.01	0.001	0.001	0.02
MNAR (moderate)	0.003	0.001	0.02	0.002	0.001	<0.01
MNAR (strong)	0.006	0.001	<0.01	0.002	0.001	<0.01
**Proportion missing**						
0.5x	Ref			Ref		
1x	0.040	0.002	<0.01	0.037	0.002	<0.01
2x	0.140	0.002	<0.01	0.138	0.002	<0.01
**Variable group**						
3	Ref			Ref		
4	0.228	0.002	<0.01	0.225	0.002	<0.01
**Imputation method * proportion missing**						
LOCF * 0.5x	Ref			Ref		
Mean * 1x	-0.039	0.002	<0.01	-0.038	0.002	<0.01
Random forest * 1x	0.032	0.002	<0.01	0.032	0.002	<0.01
Bayesian/PMM * 1x	0.086	0.002	<0.01	0.087	0.002	<0.01
LASSO * 1x	0.094	0.002	<0.01	0.091	0.002	<0.01
Mean * 2x	-0.137	0.002	<0.01	-0.137	0.002	<0.01
Random forest * 2x	0.073	0.002	<0.01	0.072	0.002	<0.01
Bayesian/PMM * 2x	0.143	0.002	<0.01	0.141	0.002	<0.01

	Extubation			Blood Pressure		
**Variable**	**Coefficient**	**Standard error**	**p-value**	**Coefficient**	**Standard error**	**p-value**
LASSO * 2x	0.151	0.002	<0.01	0.148	0.002	<0.01
**Imputation method * variable group**						
LOCF * 3	Ref			Ref		
Mean * 4	-0.298	0.002	<0.01	-0.297	0.002	<0.01
Random forest * 4	-0.010	0.002	<0.01	-0.009	0.002	<0.01
Bayesian/PMM * 4	-0.086	0.002	<0.01	-0.085	0.002	<0.01
LASSO * 4	-0.035	0.002	<0.01	-0.038	0.002	<0.01
**Missingness mechanism * proportion missing**				eliminated from this model		
MCAR * 0.5x	Ref					
MAR * 1x	-0.001	-0.001	-0.001			
MNAR (weak) * 1x	-0.005	-0.005	-0.005			
MNAR (moderate) * 1x	0.000	0.000	0.000			
MNAR (strong) * 1x	-0.002	-0.002	-0.002			
MAR * 2x	-0.004	-0.004	-0.004			
MNAR (weak) * 2x	-0.006	-0.006	-0.006			
MNAR (moderate) * 2x	-0.001	-0.001	-0.001			
MNAR (strong) * 2x	-0.003	-0.003	-0.003			
**Missingness mechanism * variable group**				eliminated from this model		
MCAR * 3	Ref					
MAR * 4	-0.003	-0.003	-0.003			
MNAR (weak) * 4	-0.001	-0.001	-0.001			
MNAR (moderate) * 4	-0.001	-0.001	-0.001			
MNAR (strong) * 1x	-0.004	-0.004	-0.004			
**Proportion missing * variable group**						
0.5x * 3	Ref			Ref		
1x * 4	-0.017	-0.017	-0.017	-0.015	-0.015	-0.015
2x * 4	-0.053	-0.053	-0.053	-0.053	-0.053	-0.053
**Imputation method * proportion missing * variable group**						
LOCF * 0.5x * 3	Ref			Ref		
Mean * 1x * 4	0.019	0.019	0.019	0.017	0.017	0.017

	Extubation			Blood Pressure		
**Variable**	**Coefficient**	**Standard error**	**p-value**	**Coefficient**	**Standard error**	**p-value**
Random forest * 1x * 4	-0.027	-0.027	-0.027	-0.029	-0.029	-0.029
Bayesian/PMM * 1x * 4	-0.070	-0.070	-0.070	-0.074	-0.074	-0.074
LASSO * 1x * 4	-0.092	-0.092	-0.092	-0.091	-0.091	-0.091
Mean * 2x * 4	0.052	0.052	0.052	0.052	0.052	0.052
Random forest * 2x * 4	-0.063	-0.063	-0.063	-0.064	-0.064	-0.064
Bayesian/PMM * 2x * 4	-0.124	-0.124	-0.124	-0.123	-0.123	-0.123
LASSO * 2x * 4	-0.145	-0.145	-0.145	-0.144	-0.144	-0.144

Note: Linear models of classification error comparing imputed values to complete dataset (each model had one observation [n=9,000] per 6 variables per 300 datasets per 5 imputation methods) with random intercepts for each of the 1500 datasets. We included all three-way and two-way interactions and completed a backward stepwise elimination procedure (included $p<0.05$) to determine the final model.

Abbreviations: LOCF = last observation carried forward; Bayesian/PMM = Bayesian imputation under the normal linear model with predictive mean matching; LASSO = Least Absolute Shrinkage and Selection Operator; MCAR = missing completely at random; MAR = missing at random; MNAR = missing not at random

Supplementary Table 6. Predictive model performance: GBM models

	Extubation						Blood Pressure		
	Balanced Accuracy			AUC			Mean Squared Error		
**Variable**	**Coefficient**	**Standard error**	**p-value**	**Coefficient**	**Standard error**	**p-value**	**Coefficient**	**Standard error**	**p-value**
(Intercept)	79.06	0.11	<0.01	86.87	0.07	<0.01	2.76	0.00	<0.01
**Imputation method**									
LOCF	Ref			Ref			Ref		
Mean	-0.30	0.13	0.03	-0.09	0.09	0.32	0.0022	0.0006	<0.01
Random forest AV	-0.15	0.13	0.25	0.15	0.09	0.11	0.0010	0.0006	0.07
Random forest MI	-0.11	0.13	0.43	0.22	0.09	0.02	0.0007	0.0006	0.24
Bayesian/PMM AV	-1.24	0.13	<0.01	-0.36	0.09	<0.01	0.0010	0.0006	0.07
Bayesian/PMM MI	-0.97	0.13	<0.01	-0.16	0.09	0.08	0.0014	0.0006	0.01
LASSO AV	-1.24	0.13	<0.01	-0.38	0.09	<0.01	0.0020	0.0006	<0.01
LASSO MI	-1.13	0.13	<0.01	-0.18	0.09	0.05	0.0016	0.0006	<0.01
None	-0.17	0.13	0.21	-0.07	0.09	0.48	0.0026	0.0006	<0.01
**Missingness mechanism**									
MCAR	Ref			Ref			Ref		
MAR	-0.05	0.10	0.63	0.05	0.09	0.53	-0.0004	0.0006	0.53
MNAR (weak)	0.17	0.10	0.10	0.15	0.09	0.09	-0.0009	0.0006	0.13
MNAR (moderate)	0.16	0.10	0.11	0.13	0.09	0.13	-0.0004	0.0006	0.49
MNAR (strong)	0.12	0.10	0.22	0.10	0.09	0.25	-0.0006	0.0006	0.33
**Proportion missing**									
0.5x	Ref			Ref			Ref		
1x	-0.15	0.16	0.36	-0.28	0.07	<0.01	0.0006	0.0006	0.26
2x	-0.48	0.16	<0.01	-0.59	0.07	<0.01	0.0039	0.0006	<0.01
**Imputation method * missingness mechanism**	eliminated from this model								
LOCF * MCAR				Ref			Ref		
Mean * MAR				0.02	0.11	0.88	-0.0001	0.0008	0.86
Random forest AV * MAR				-0.13	0.11	0.26	0.0004	0.0008	0.63
Random forest MI * MAR				-0.05	0.11	0.67	0.0006	0.0008	0.48

	Extubation						Blood Pressure		
	Balanced Accuracy			AUC			Mean Squared Error		
Variable	Coefficient	Standard error	p-value	Coefficient	Standard error	p-value	Coefficient	Standard error	p-value
Bayesian/PMM AV * MAR				0.11	0.11	0.31	0.0014	0.0008	0.08
Bayesian/PMM MI * MAR				0.05	0.11	0.62	0.0007	0.0008	0.41
LASSO AV * MAR				0.01	0.11	0.95	0.0002	0.0008	0.83
LASSO MI * MAR				0.02	0.11	0.85	0.0007	0.0008	0.42
None * MAR				0.02	0.11	0.85	0.0001	0.0008	0.88
Mean * MNAR (weak)				0.05	0.11	0.65	-0.0008	0.0008	0.30
Random forest AV * MNAR (weak)				-0.04	0.11	0.70	0.0004	0.0008	0.65
Random forest MI * MNAR (weak)				-0.05	0.11	0.63	0.0009	0.0008	0.26
Bayesian/PMM AV * MNAR (weak)				0.05	0.11	0.68	0.0008	0.0008	0.34
Bayesian/PMM MI * MNAR (weak)				-0.01	0.11	0.96	0.0011	0.0008	0.18
LASSO AV * MNAR (weak)				0.05	0.11	0.67	0.0008	0.0008	0.35
LASSO MI * MNAR (weak)				0.01	0.11	0.90	0.0010	0.0008	0.21
None * MNAR (weak)				0.16	0.11	0.14	-0.0016	0.0008	0.05
Mean * MNAR (moderate)				0.16	0.11	0.16	-0.0003	0.0008	0.73
Random forest AV * MNAR (moderate)				-0.01	0.11	0.93	-0.0003	0.0008	0.68
Random forest MI * MNAR (moderate)				-0.05	0.11	0.64	0.0007	0.0008	0.38
Bayesian/PMM AV * MNAR (moderate)				-0.01	0.11	0.91	-0.0004	0.0008	0.60
Bayesian/PMM MI * MNAR (moderate)				-0.04	0.11	0.75	0.0003	0.0008	0.71
LASSO AV * MNAR (moderate)				-0.06	0.11	0.62	-0.0010	0.0008	0.23
LASSO MI * MNAR (moderate)				0.01	0.11	0.93	-0.0001	0.0008	0.94
None * MNAR (moderate)				0.28	0.11	0.01	-0.0011	0.0008	0.16
Mean * MNAR (strong)				0.42	0.11	<0.01	-0.0052	0.0008	<0.01
Random forest AV * MNAR (strong)				0.13	0.11	0.25	-0.0010	0.0008	0.22
Random forest MI * MNAR (strong)				-0.01	0.11	0.94	0.0008	0.0008	0.31

	Extubation						Blood Pressure		
	Balanced Accuracy			AUC			Mean Squared Error		
**Variable**	**Coefficient**	**Standard error**	**p-value**	**Coefficient**	**Standard error**	**p-value**	**Coefficient**	**Standard error**	**p-value**
Bayesian/PMM AV * MNAR (strong)				0.34	0.11	<0.01	-0.0008	0.0008	0.34
Bayesian/PMM MI * MNAR (strong)				0.13	0.11	0.25	0.0007	0.0008	0.41
LASSO AV * MNAR (strong)				0.26	0.11	0.02	-0.0027	0.0008	<0.01
LASSO MI * MNAR (strong)				0.24	0.11	0.03	-0.0022	0.0008	<0.01
None * MNAR (strong)				0.63	0.11	<0.01	-0.0072	0.0008	<0.01
**Imputation method * proportion missing**									
LOCF * 0.5x	Ref			Ref			Ref		
Mean * 1x	0.06	0.19	0.76	-0.11	0.09	0.19	0.0018	0.0008	0.03
Random forest AV * 1x	-0.40	0.19	0.04	-0.18	0.09	0.04	0.0012	0.0008	0.15
Random forest MI * 1x	-0.07	0.19	0.72	0.04	0.09	0.68	0.0021	0.0008	<0.01
Bayesian/PMM AV * 1x	-1.97	0.19	<0.01	-0.53	0.09	<0.01	0.0029	0.0008	<0.01
Bayesian/PMM MI * 1x	-0.97	0.19	<0.01	-0.16	0.09	0.06	0.0020	0.0008	0.01
LASSO AV * 1x	-1.83	0.19	<0.01	-0.53	0.09	<0.01	0.0025	0.0008	<0.01
LASSO MI * 1x	-1.19	0.19	<0.01	-0.18	0.09	0.04	0.0021	0.0008	<0.01
None * 1x	-0.08	0.19	0.66	-0.23	0.09	<0.01	0.0013	0.0008	0.11
Mean * 2x	-0.11	0.19	0.57	-0.65	0.09	<0.01	0.0007	0.0008	0.37
Random forest AV * 2x	-3.08	0.19	<0.01	-1.30	0.09	<0.01	0.0011	0.0008	0.19
Random forest MI * 2x	-2.04	0.19	<0.01	-0.42	0.09	<0.01	0.0016	0.0008	0.05
Bayesian/PMM AV * 2x	-10.21	0.19	<0.01	-2.66	0.09	<0.01	0.0020	0.0008	0.01
Bayesian/PMM MI * 2x	-4.02	0.19	<0.01	-0.66	0.09	<0.01	0.0011	0.0008	0.18
LASSO AV * 2x	-9.94	0.19	<0.01	-2.51	0.09	<0.01	0.0007	0.0008	0.38
LASSO MI * 2x	-4.63	0.19	<0.01	-0.80	0.09	<0.01	0.0010	0.0008	0.23
None * 2x	-0.38	0.19	0.05	-0.85	0.09	<0.01	0.0003	0.0008	0.70
**Missingness mechanism * proportion missing**									
MCAR * 0.5x	Ref			Ref			Ref		

	Extubation						Blood Pressure		
	Balanced Accuracy			AUC			Mean Squared Error		
**Variable**	**Coefficient**	**Standard error**	**p-value**	**Coefficient**	**Standard error**	**p-value**	**Coefficient**	**Standard error**	**p-value**
MAR * 1x	0.16	0.14	0.25	0.05	0.06	0.43	0.0008	0.0008	0.35
MNAR (weak) * 1x	0.13	0.14	0.35	-0.01	0.06	0.83	0.0008	0.0008	0.33
MNAR (moderate) * 1x	-0.18	0.14	0.20	-0.05	0.06	0.46	0.0005	0.0008	0.54
MNAR (strong) * 1x	0.16	0.14	0.26	0.17	0.06	<0.01	0.0006	0.0008	0.45
MAR * 2x	0.47	0.14	<0.01	0.08	0.06	0.22	-0.0006	0.0008	0.48
MNAR (weak) * 2x	-0.12	0.14	0.39	-0.18	0.06	<0.01	0.0003	0.0008	0.72
MNAR (moderate) * 2x	-0.15	0.14	0.28	-0.20	0.06	<0.01	0.0002	0.0008	0.80
MNAR (strong) * 2x	0.18	0.14	0.19	-0.10	0.06	0.12	-0.0005	0.0008	0.50
**Imputation method * missingness mechanism * proportion missing**	eliminated from this model			eliminated from this model					
LOCF * MCAR * 0.5x							Ref		
Mean * MAR * 1x							-0.0004	0.0012	0.72
Random forest AV * MAR * 1x							0.0002	0.0012	0.85
Random forest MI * MAR * 1x							-0.0008	0.0012	0.47
Bayesian/PMM AV * MAR * 1x							-0.0009	0.0012	0.42
Bayesian/PMM MI * MAR * 1x							-0.0012	0.0012	0.31
LASSO AV * MAR * 1x							-0.0014	0.0012	0.24
LASSO MI * MAR * 1x							-0.0012	0.0012	0.32
None * MAR * 1x							-0.0008	0.0012	0.46
Mean * MNAR (weak) * 1x							0.0006	0.0012	0.58
Random forest AV * MNAR (weak) * 1x							-0.0005	0.0012	0.67
Random forest MI * MNAR (weak) * 1x							-0.0008	0.0012	0.47
Bayesian/PMM AV * MNAR (weak) * 1x							-0.0005	0.0012	0.66
Bayesian/PMM MI * MNAR (weak) * 1x							-0.0009	0.0012	0.42
LASSO AV * MNAR (weak) * 1x							-0.0014	0.0012	0.24
LASSO MI * MNAR (weak) * 1x							-0.0011	0.0012	0.35
None * MNAR (weak) * 1x							0.0013	0.0012	0.26
Mean * MNAR (moderate) * 1x							0.0003	0.0012	0.80

	Extubation						Blood Pressure		
	Balanced Accuracy			AUC			Mean Squared Error		
**Variable**	**Coefficient**	**Standard error**	**p-value**	**Coefficient**	**Standard error**	**p-value**	**Coefficient**	**Standard error**	**p-value**
Random forest AV * MNAR (moderate) * 1x							0.0005	0.0012	0.69
Random forest MI * MNAR (moderate) * 1x							-0.0008	0.0012	0.49
Bayesian/PMM AV * MNAR (moderate) * 1x							0.0002	0.0012	0.83
Bayesian/PMM MI * MNAR (moderate) * 1x							-0.0004	0.0012	0.72
LASSO AV * MNAR (moderate) * 1x							0.0004	0.0012	0.70
LASSO MI * MNAR (moderate) * 1x							0.0000	0.0012	0.99
None * MNAR (moderate) * 1x							0.0009	0.0012	0.41
Mean * MNAR (strong) * 1x							0.0052	0.0012	<0.01
Random forest AV * MNAR (strong) * 1x							0.0024	0.0012	0.04
Random forest MI * MNAR (strong) * 1x							-0.0007	0.0012	0.54
Bayesian/PMM AV * MNAR (strong) * 1x							0.0009	0.0012	0.45
Bayesian/PMM MI * MNAR (strong) * 1x							-0.0005	0.0012	0.67
LASSO AV * MNAR (strong) * 1x							0.0028	0.0012	0.02
LASSO MI * MNAR (strong) * 1x							0.0023	0.0012	0.05
None * MNAR (strong) * 1x							0.0070	0.0012	<0.01
Mean * MAR * 2x							0.0015	0.0012	0.20
Random forest AV * MAR * 2x							0.0007	0.0012	0.55
Random forest MI * MAR * 2x							0.0004	0.0012	0.71
Bayesian/PMM AV * MAR * 2x							0.0001	0.0012	0.91
Bayesian/PMM MI * MAR * 2x							0.0005	0.0012	0.69
LASSO AV * MAR * 2x							0.0018	0.0012	0.13
LASSO MI * MAR * 2x							0.0004	0.0012	0.74
None * MAR * 2x							0.0011	0.0012	0.34
Mean * MNAR (weak) * 2x							0.0018	0.0012	0.13
Random forest AV * MNAR (weak) * 2x							0.0004	0.0012	0.75
Random forest MI * MNAR (weak) * 2x							-0.0004	0.0012	0.71
Bayesian/PMM AV * MNAR (weak) * 2x							0.0003	0.0012	0.82
Bayesian/PMM MI * MNAR (weak) * 2x							-0.0004	0.0012	0.76

	Extubation						Blood Pressure		
	Balanced Accuracy			AUC			Mean Squared Error		
**Variable**	**Coefficient**	**Standard error**	**p-value**	**Coefficient**	**Standard error**	**p-value**	**Coefficient**	**Standard error**	**p-value**
LASSO AV * MNAR (weak) * 2x							0.0002	0.0012	0.86
LASSO MI * MNAR (weak) * 2x							-0.0003	0.0012	0.77
None * MNAR (weak) * 2x							0.0022	0.0012	0.05
Mean * MNAR (moderate) * 2x							0.0005	0.0012	0.66
Random forest AV * MNAR (moderate) * 2x							0.0008	0.0012	0.50
Random forest MI * MNAR (moderate) * 2x							-0.0005	0.0012	0.68
Bayesian/PMM AV * MNAR (moderate) * 2x							0.0009	0.0012	0.41
Bayesian/PMM MI * MNAR (moderate) * 2x							0.0001	0.0012	0.91
LASSO AV * MNAR (moderate) * 2x							0.0022	0.0012	0.06
LASSO MI * MNAR (moderate) * 2x							0.0004	0.0012	0.76
None * MNAR (moderate) * 2x							0.0017	0.0012	0.15
Mean * MNAR (strong) * 2x							0.0067	0.0012	<0.01
Random forest AV * MNAR (strong) * 2x							0.0022	0.0012	0.06
Random forest MI * MNAR (strong) * 2x							0.0003	0.0012	0.78
Bayesian/PMM AV * MNAR (strong) * 2x							0.0023	0.0012	0.04
Bayesian/PMM MI * MNAR (strong) * 2x							0.0004	0.0012	0.71
LASSO AV * MNAR (strong) * 2x							0.0046	0.0012	<0.01
LASSO MI * MNAR (strong) * 2x							0.0033	0.0012	<0.01
None * MNAR (strong) * 2x							0.0089	0.0012	<0.01

Note: Linear models of predictive model performance (for each model: one observation [n=2,700] per 300 datasets per 9 imputation methods). We included all three-way and two-way interactions and completed a backward stepwise elimination procedure (included p<0.05) to determine the final model.

Abbreviations: LOCF = last observation carried forward; Bayesian/PMM = Bayesian imputation under the normal linear model with predictive mean matching; LASSO = Least Absolute Shrinkage and Selection Operator; AV = average; MI = multiple imputation; MCAR = missing completely at random; MAR = missing at random; MNAR = missing not at random

Supplementary Table 7. Predictive model performance: LASSO models

	Extubation						Blood Pressure		
	Balanced Accuracy			AUC			Mean Squared Error		
**Variable**	**Coefficient**	**Standard error**	**p-value**	**Coefficient**	**Standard error**	**p-value**	**Coefficient**	**Standard error**	**p-value**
(Intercept)	78.96	0.10	<0.01	87.38	0.04	<0.01	2.79	0.00	<0.01
**Imputation method**									
LOCF	Ref			Ref			Ref		
Mean	-0.68	0.12	<0.01	-0.49	0.05	<0.01	0.0017	0.0002	<0.01
Random forest AV	-0.25	0.12	0.03	-0.05	0.05	0.25	0.0001	0.0002	0.61
Random forest MI	-0.27	0.12	0.02	-0.05	0.05	0.32	0.0008	0.0002	<0.01
Bayesian/PMM AV	-1.07	0.12	<0.01	-0.32	0.05	<0.01	0.0011	0.0002	<0.01
Bayesian/PMM MI	-1.05	0.12	<0.01	-0.33	0.05	<0.01	0.0018	0.0002	<0.01
LASSO AV	-1.06	0.12	<0.01	-0.32	0.05	<0.01	0.0012	0.0002	<0.01
LASSO MI	-0.95	0.12	<0.01	-0.30	0.05	<0.01	0.0016	0.0002	<0.01
**Missingness mechanism**									
MCAR	Ref			Ref			Ref		
MAR	0.07	0.09	0.47	0.08	0.04	0.04	-0.0001	0.0001	0.45
MNAR (weak)	0.07	0.09	0.45	0.05	0.04	0.18	0.0001	0.0001	0.37
MNAR (moderate)	-0.13	0.09	0.17	-0.05	0.04	0.19	-0.0001	0.0001	0.37
MNAR (strong)	-0.05	0.09	0.58	0.02	0.04	0.65	0.0000	0.0001	0.76
**Proportion missing**									
0.5x	Ref			Ref			Ref		
1x	-0.38	0.14	<0.01	-0.27	0.06	<0.01	0.0004	0.0002	0.11
2x	-1.03	0.14	<0.01	-0.83	0.06	<0.01	0.0010	0.0002	<0.01
**Imputation method * proportion missing**									
LOCF * 0.5x	Ref			Ref			Ref		
Mean * 1x	-0.32	0.16	0.05	-0.38	0.07	<0.01	0.0009	0.0003	<0.01
Random forest AV * 1x	-0.44	0.16	<0.01	-0.15	0.07	0.03	0.0000	0.0003	0.95
Random forest MI * 1x	-0.47	0.16	<0.01	-0.17	0.07	<0.01	0.0008	0.0003	0.01
Bayesian/PMM AV * 1x	-1.23	0.16	<0.01	-0.40	0.07	<0.01	0.0011	0.0003	<0.01
Bayesian/PMM MI * 1x	-0.95	0.16	<0.01	-0.42	0.07	<0.01	0.0011	0.0003	<0.01

LASSO AV * 1x	-1.13	0.16	<0.01	-0.40	0.07	<0.01	0.0003	0.0003	0.30
LASSO MI * 1x	-1.01	0.16	<0.01	-0.39	0.07	<0.01	0.0010	0.0003	<0.01
Mean * 2x	-1.21	0.16	<0.01	-1.04	0.07	<0.01	0.0022	0.0003	<0.01
Random forest AV * 2x	-2.84	0.16	<0.01	-0.84	0.07	<0.01	0.0012	0.0003	<0.01
Random forest MI * 2x	-2.13	0.16	<0.01	-0.92	0.07	<0.01	0.0023	0.0003	<0.01
Bayesian/PMM AV * 2x	-6.16	0.16	<0.01	-1.70	0.07	<0.01	0.0020	0.0003	<0.01
Bayesian/PMM MI * 2x	-2.92	0.16	<0.01	-1.34	0.07	<0.01	0.0018	0.0003	<0.01
LASSO AV * 2x	-5.51	0.16	<0.01	-1.55	0.07	<0.01	0.0015	0.0003	<0.01
LASSO MI * 2x	-3.05	0.16	<0.01	-1.23	0.07	<0.01	0.0018	0.0003	<0.01
**Missingness mechanism * proportion missing**							eliminated from this model		
MCAR * 0.5x	Ref			Ref					
MAR * 1x	0.15	0.13	0.23	0.06	0.05	0.27			
MNAR (weak) * 1x	0.30	0.13	0.02	0.14	0.05	<0.01			
MNAR (moderate) * 1x	0.43	0.13	<0.01	0.20	0.05	<0.01			
MNAR (strong) * 1x	0.41	0.13	<0.01	0.34	0.05	<0.01			
MAR * 2x	0.58	0.13	<0.01	0.04	0.05	0.41			
MNAR (weak) * 2x	0.25	0.13	0.05	-0.04	0.05	0.50			
MNAR (moderate) * 2x	0.42	0.13	<0.01	0.13	0.05	0.02			
MNAR (strong) * 2x	0.73	0.13	<0.01	0.20	0.05	<0.01			

Note: Linear models of predictive model performance (for each model: one observation [n=2,400] per 300 datasets per 8 imputation methods). We included all three-way and two-way interactions and completed a backward stepwise elimination procedure (included $p<0.05$) to determine the final model.

Abbreviations: LOCF = last observation carried forward; Bayesian/PMM = Bayesian imputation under the normal linear model with predictive mean matching; LASSO = Least Absolute Shrinkage and Selection Operator; AV = average; MI = multiple imputation; MCAR = missing completely at random; MAR = missing at random; MNAR = missing not at random
