# Supplementary figures and images for: Methods for Addressing Missingness in Electronic Health Record Data for Clinical Prediction Models: Comparative Evaluation
Source: JMIR Med Inform. 2025 Nov 14;13:e79307. doi: 10.2196/79307 (PMC12617989; doi:10.2196/79307)

# Blood Pressure Imputation Test Performance Metrics: Mean Squared Error

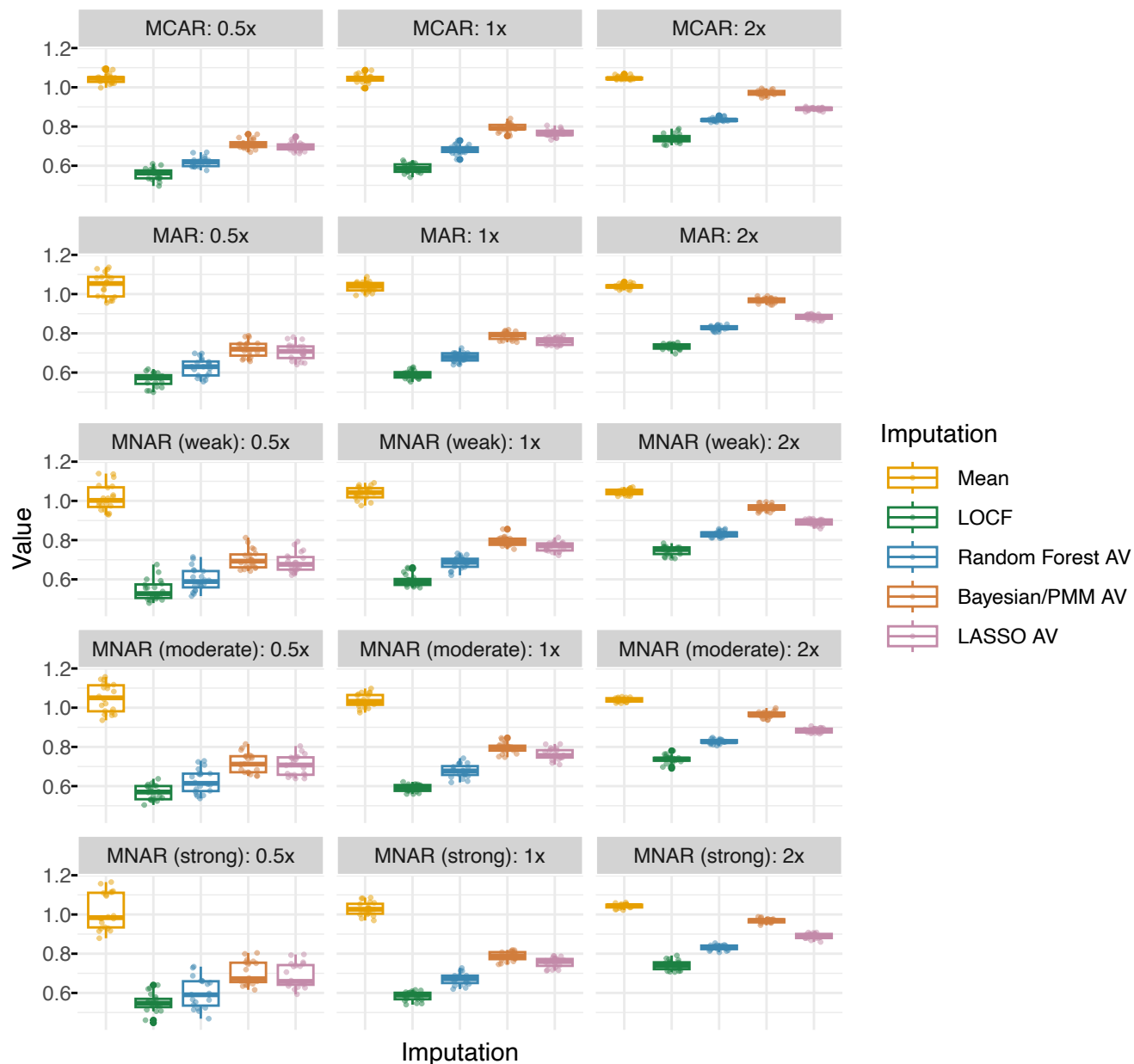

Supplement: Multimedia Appendix 2 [file medinform-v13-e79307-s002.pdf]

# Autocorrelation vs. Imputation Error by Method – Outcome: Extubation

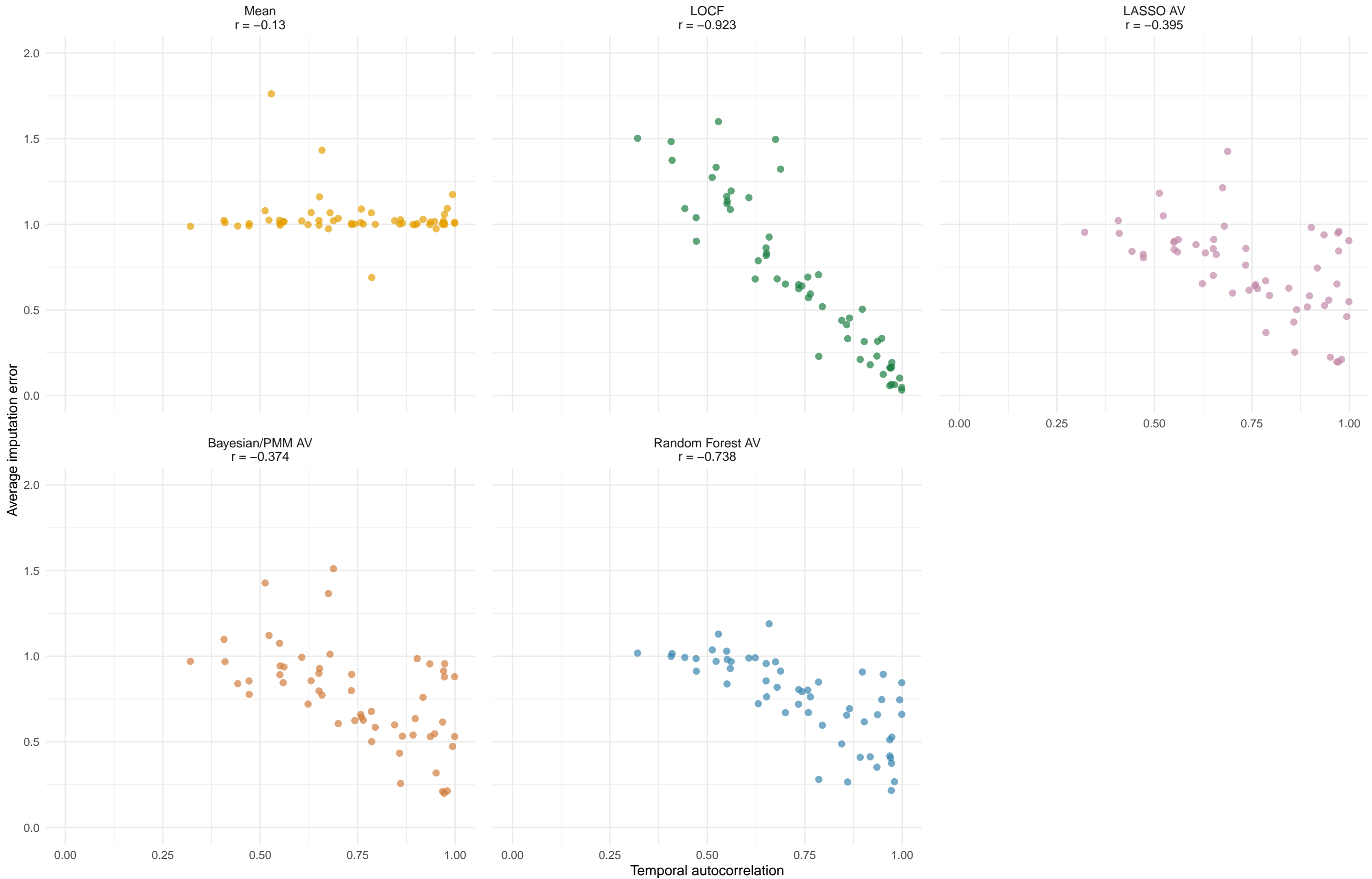

Supplement: Multimedia Appendix 6 [file medinform-v13-e79307-s006.pdf]
