# Supplementary material for: Methods for Addressing Missingness in Electronic Health Record Data for Clinical Prediction Models: Comparative Evaluation
Source: JMIR Med Inform. 2025 Nov 14;13:e79307. doi: 10.2196/79307 (PMC12617989; doi:10.2196/79307)

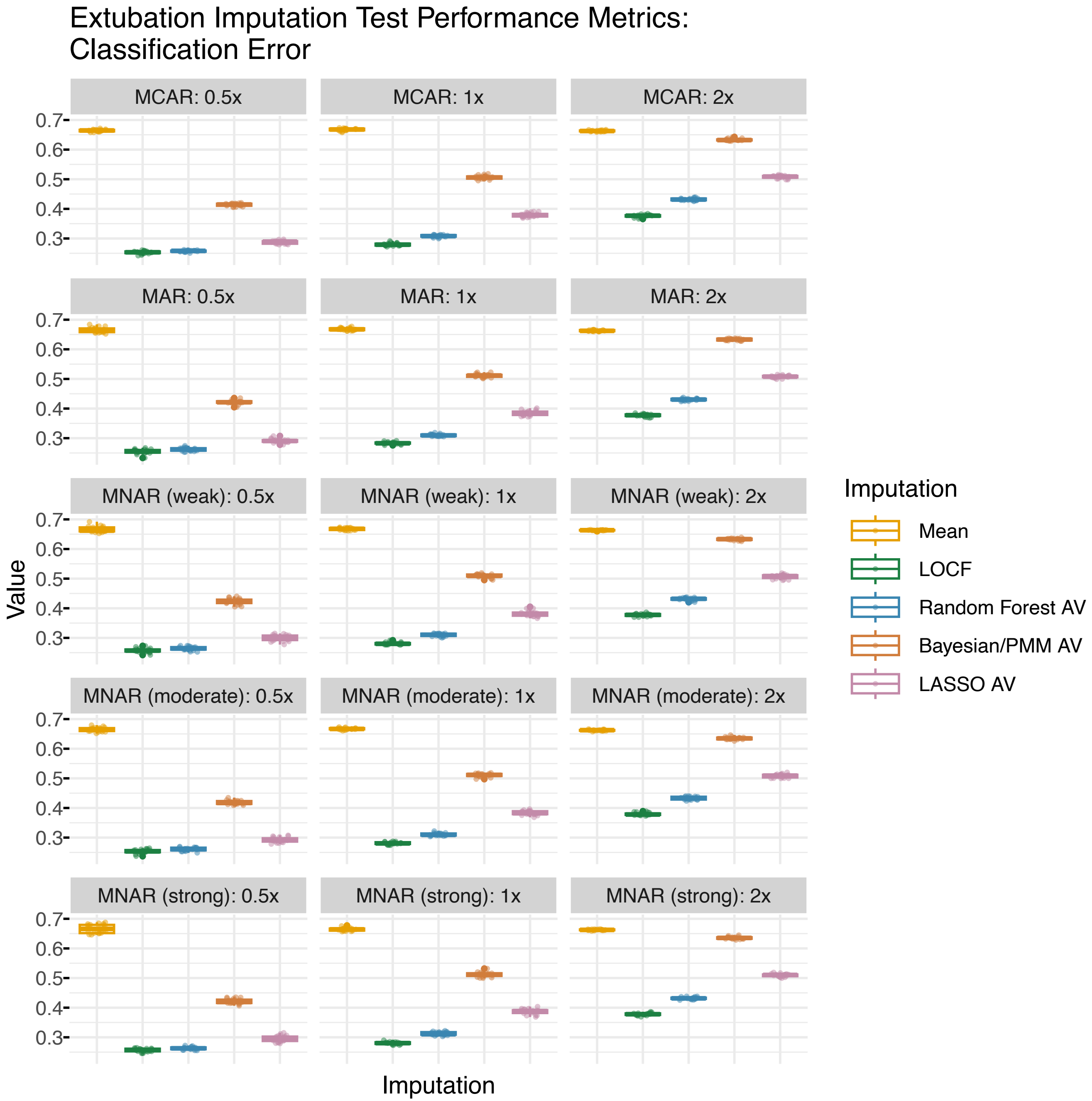

Extubation Imputation Test Performance Metrics:
Classification Error
MCAR: 0.5x
MCAR: 1x
MCAR: 2x
MAR: 0.5x
MAR: 1x
MAR: 2x
MNAR (weak): 0.5x
MNAR (weak): 1x
MNAR (weak): 2x
MNAR (moderate): 0.5x
MNAR (moderate): 1x
MNAR (moderate): 2x
MNAR (strong): 0.5x
MNAR (strong): 1x
MNAR (strong): 2x
0.7
0.6
0.5
0.4
0.3
Value
Imputation
Imputation
Mean
LOCF
Random Forest AV
Bayesian/PMM AV
LASSO AV

Blood Pressure Imputation Test Performance Metrics:
Classification Error

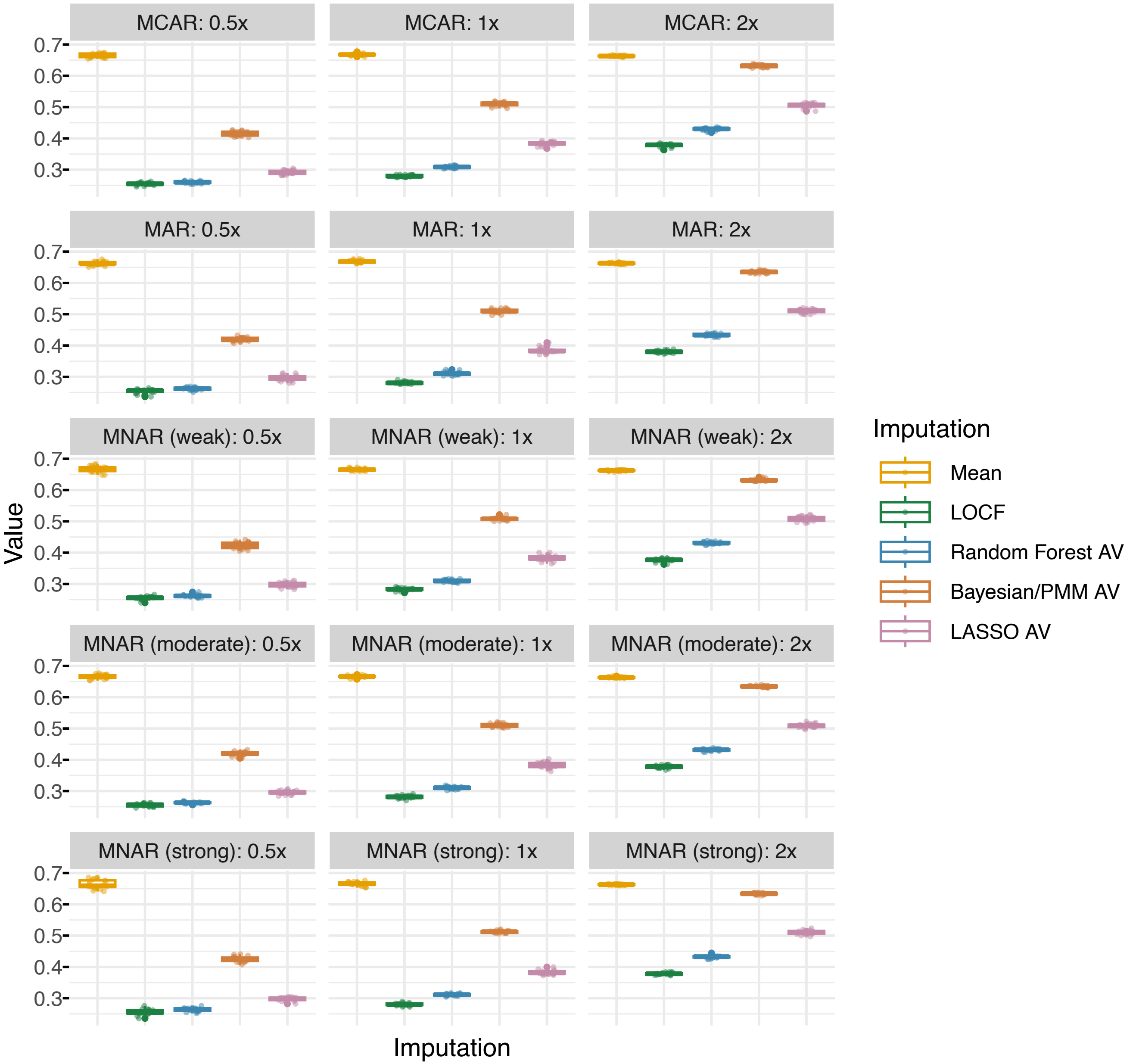

Supplement: Multimedia Appendix 3 [file medinform-v13-e79307-s003.pdf]
