# Supplementary material for: Methods for Addressing Missingness in Electronic Health Record Data for Clinical Prediction Models: Comparative Evaluation
Source: JMIR Med Inform. 2025 Nov 14;13:e79307. doi: 10.2196/79307 (PMC12617989; doi:10.2196/79307)
Supplement: Multimedia Appendix 7 [file medinform-v13-e79307-s007.pdf]

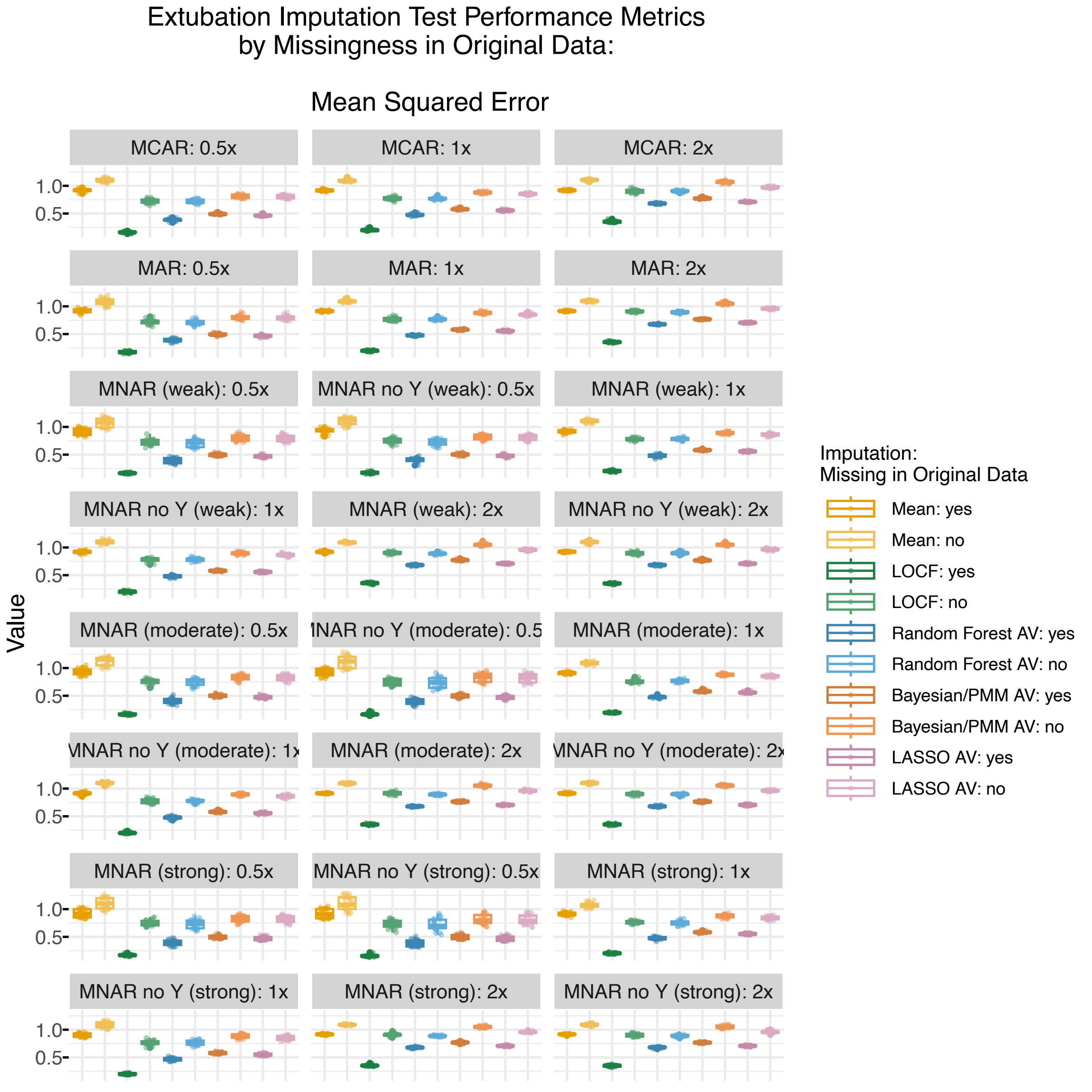
Extubation Imputation Test Performance Metrics
by Missingness in Original Data:
Mean Squared Error
MCAR: 0.5x
MCAR: 1x
MCAR: 2x
MAR: 0.5x
MAR: 1x
MAR: 2x
MNAR (weak): 0.5x
MNAR no Y (weak): 0.5x
MNAR (weak): 1x
MNAR no Y (weak): 1x
MNAR (weak): 2x
MNAR no Y (weak): 2x
MNAR (moderate): 0.5x
MNAR no Y (moderate): 0.5
MNAR (moderate): 1x
MNAR no Y (moderate): 1x
MNAR (moderate): 2x
MNAR no Y (moderate): 2x
MNAR (strong): 0.5x
MNAR no Y (strong): 0.5x
MNAR (strong): 1x
MNAR no Y (strong): 1x
MNAR (strong): 2x
MNAR no Y (strong): 2x
1.0
0.5
Value
Imputation:
Missing in Original Data
Mean: yes
Mean: no
LOCF: yes
LOCF: no
Random Forest AV: yes
Random Forest AV: no
Bayesian/PMM AV: yes
Bayesian/PMM AV: no
LASSO AV: yes
LASSO AV: no

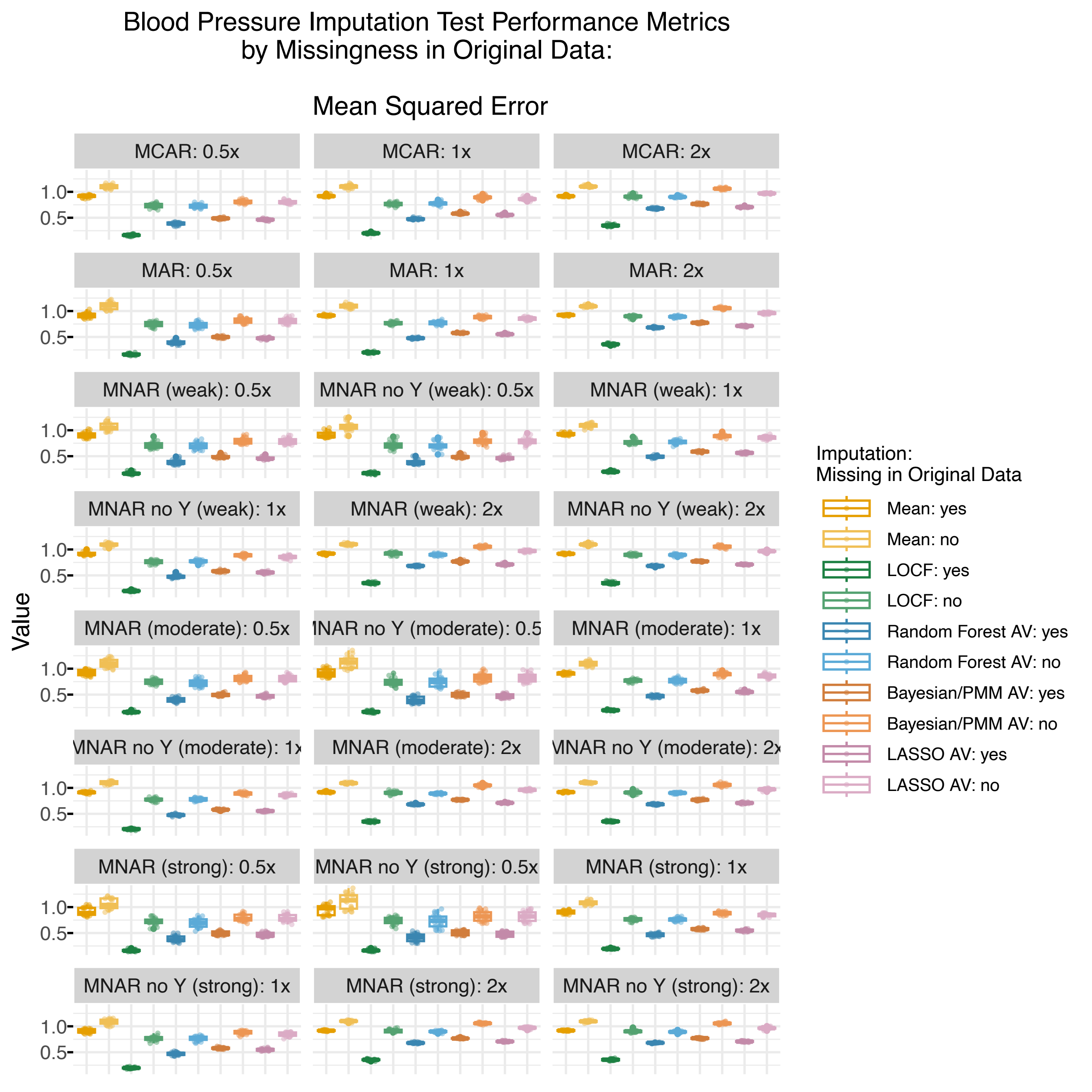

Blood Pressure Imputation Test Performance Metrics
by Missingness in Original Data:
Mean Squared Error
MCAR: 0.5x
MCAR: 1x
MCAR: 2x
MAR: 0.5x
MAR: 1x
MAR: 2x
MNAR (weak): 0.5x
MNAR no Y (weak): 0.5x
MNAR (weak): 1x
MNAR no Y (weak): 1x
MNAR (weak): 2x
MNAR no Y (weak): 2x
MNAR (moderate): 0.5x
MNAR no Y (moderate): 0.5
MNAR (moderate): 1x
MNAR no Y (moderate): 1x
MNAR (moderate): 2x
MNAR no Y (moderate): 2x
MNAR (strong): 0.5x
MNAR no Y (strong): 0.5x
MNAR (strong): 1x
MNAR no Y (strong): 1x
MNAR (strong): 2x
MNAR no Y (strong): 2x
Value
1.0
0.5
Imputation:
Missing in Original Data
Mean: yes
Mean: no
LOCF: yes
LOCF: no
Random Forest AV: yes
Random Forest AV: no
Bayesian/PMM AV: yes
Bayesian/PMM AV: no
LASSO AV: yes
LASSO AV: no
