# Supplementary material for: Methods for Addressing Missingness in Electronic Health Record Data for Clinical Prediction Models: Comparative Evaluation
Source: JMIR Med Inform. 2025 Nov 14;13:e79307. doi: 10.2196/79307 (PMC12617989; doi:10.2196/79307)
Supplement: Multimedia Appendix 8 [file medinform-v13-e79307-s008.pdf]

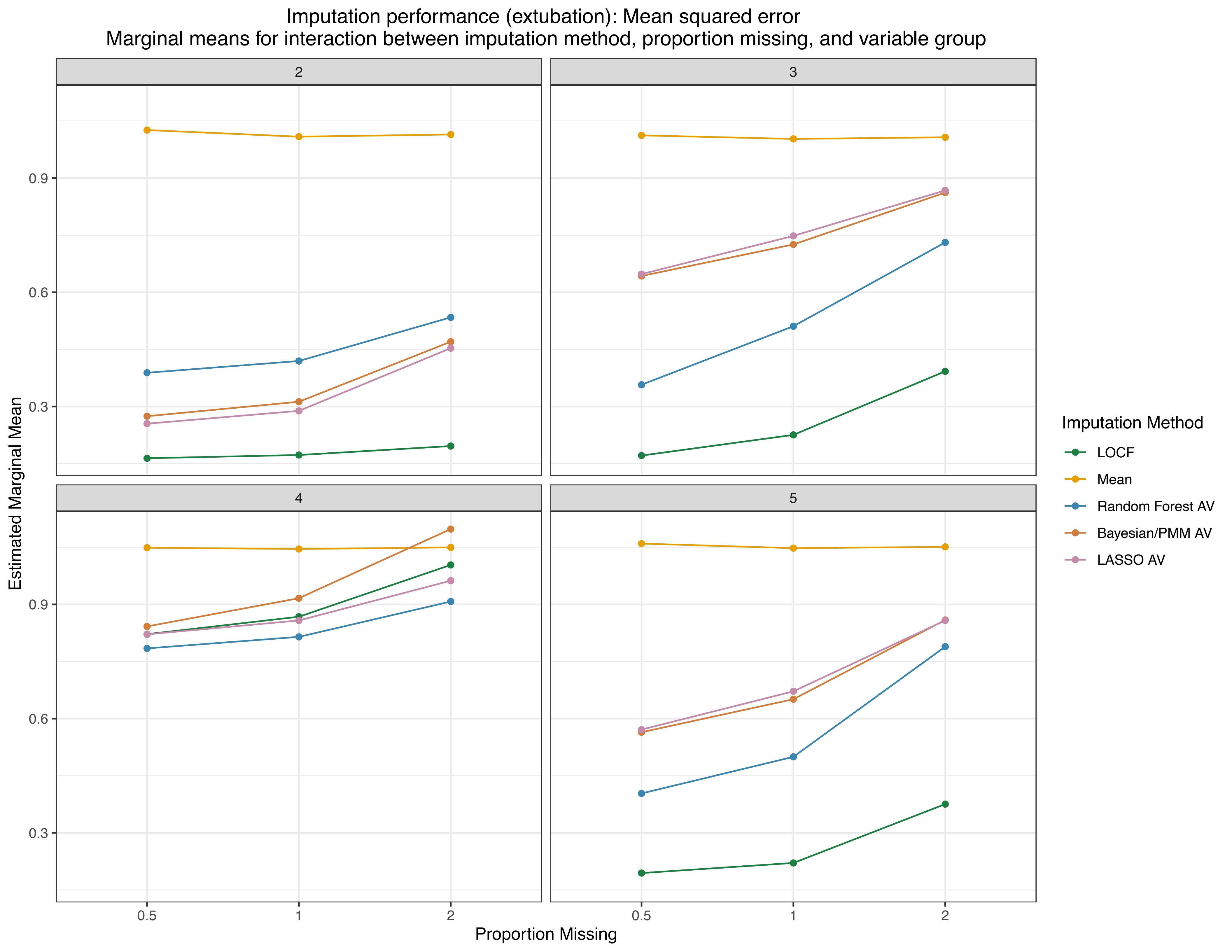

Imputation performance (extubation): Mean squared error
Marginal means for interaction between imputation method, proportion missing, and variable group
2
3
4
5
0.9
0.6
0.3
0.5
1
2
Estimated Marginal Mean
Proportion Missing
Imputation Method
LOCF
Mean
Random Forest AV
Bayesian/PMM AV
LASSO AV

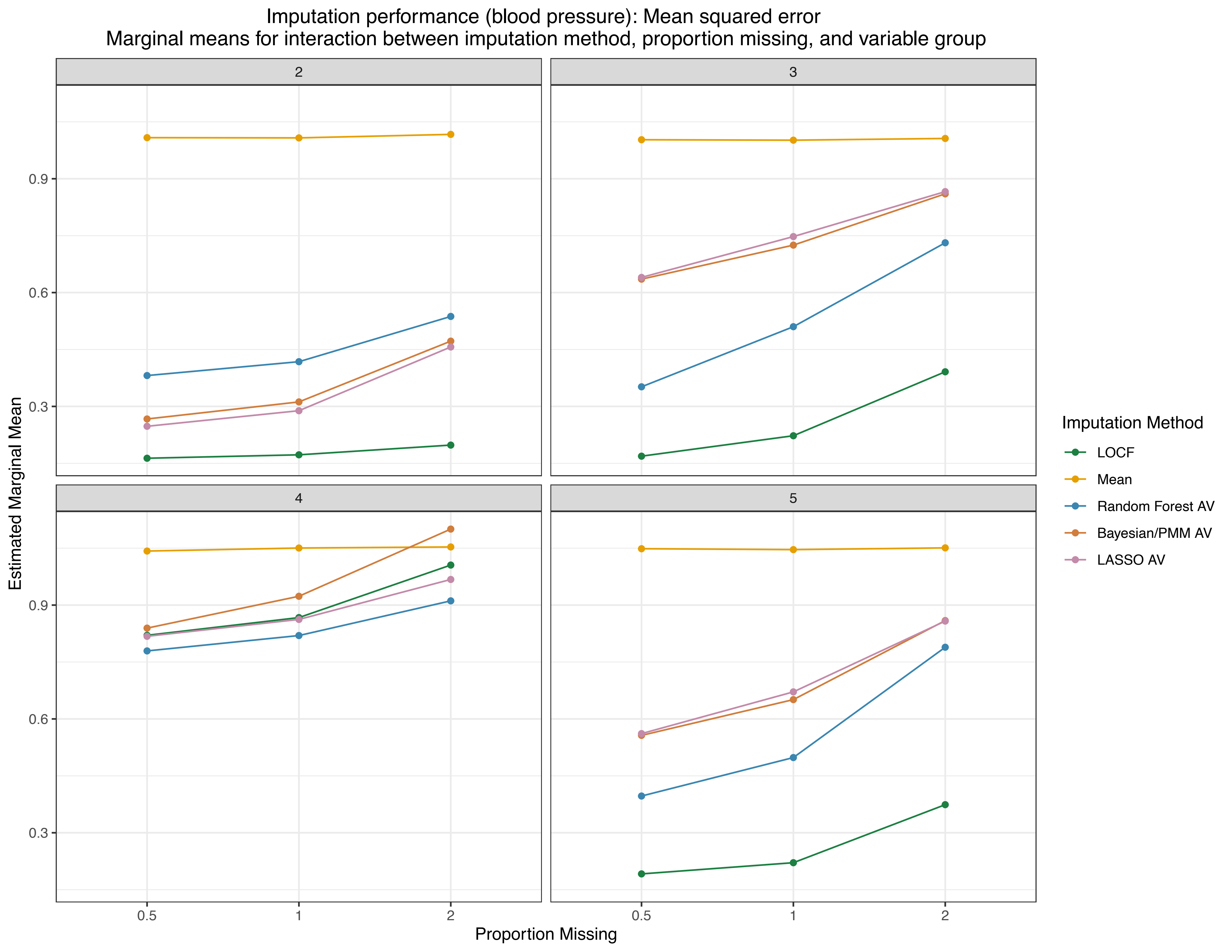

Imputation performance (blood pressure): Mean squared error
Marginal means for interaction between imputation method, proportion missing, and variable group
2
3
4
5
0.9
0.6
0.3
0.5
1
2
Estimated Marginal Mean
Proportion Missing
Imputation Method
LOCF
Mean
Random Forest AV
Bayesian/PMM AV
LASSO AV

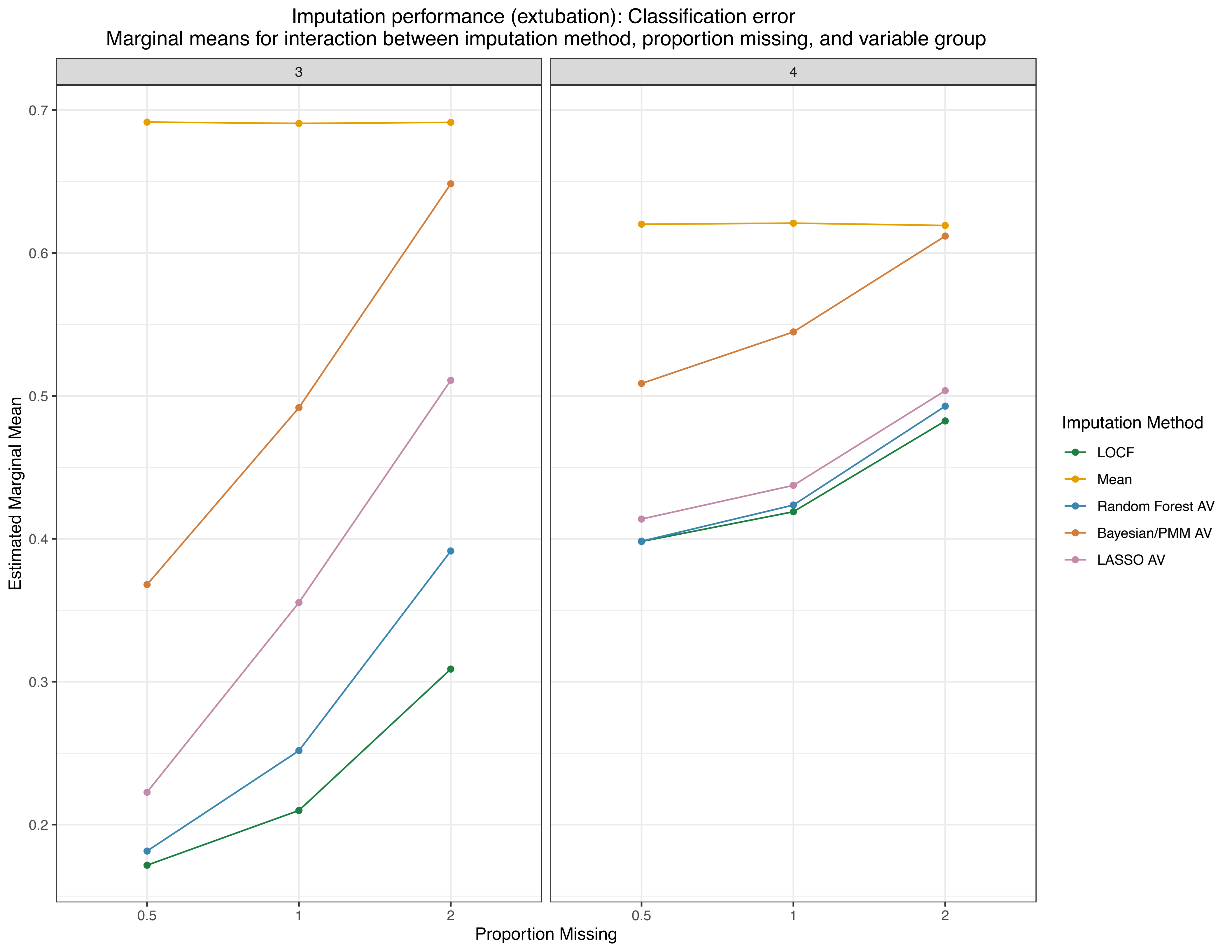
Imputation performance (extubation): Classification error
Marginal means for interaction between imputation method, proportion missing, and variable group
3
4
Estimated Marginal Mean
0.7
0.6
0.5
0.4
0.3
0.2
0.5
1
2
Proportion Missing
Imputation Method
LOCF
Mean
Random Forest AV
Bayesian/PMM AV
LASSO AV

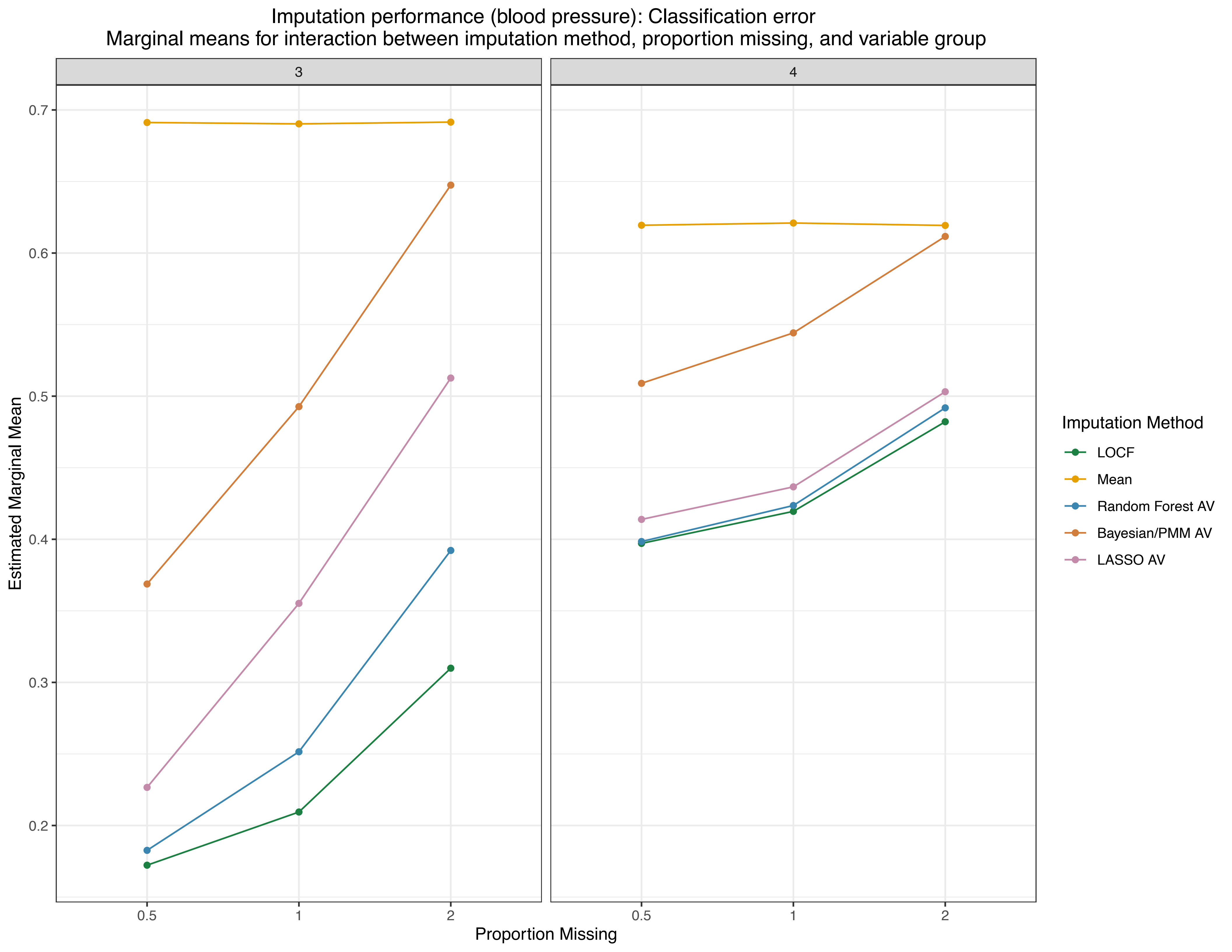

Imputation performance (blood pressure): Classification error
Marginal means for interaction between imputation method, proportion missing, and variable group
3
4
0.7
0.6
0.5
0.4
0.3
0.2
Estimated Marginal Mean
0.5
1
2
Proportion Missing
Imputation Method
LOCF
Mean
Random Forest AV
Bayesian/PMM AV
LASSO AV
