# Supplementary material for: Methods for Addressing Missingness in Electronic Health Record Data for Clinical Prediction Models: Comparative Evaluation
Source: JMIR Med Inform. 2025 Nov 14;13:e79307. doi: 10.2196/79307 (PMC12617989; doi:10.2196/79307)
Supplement: Multimedia Appendix 9 [file medinform-v13-e79307-s009.pdf]

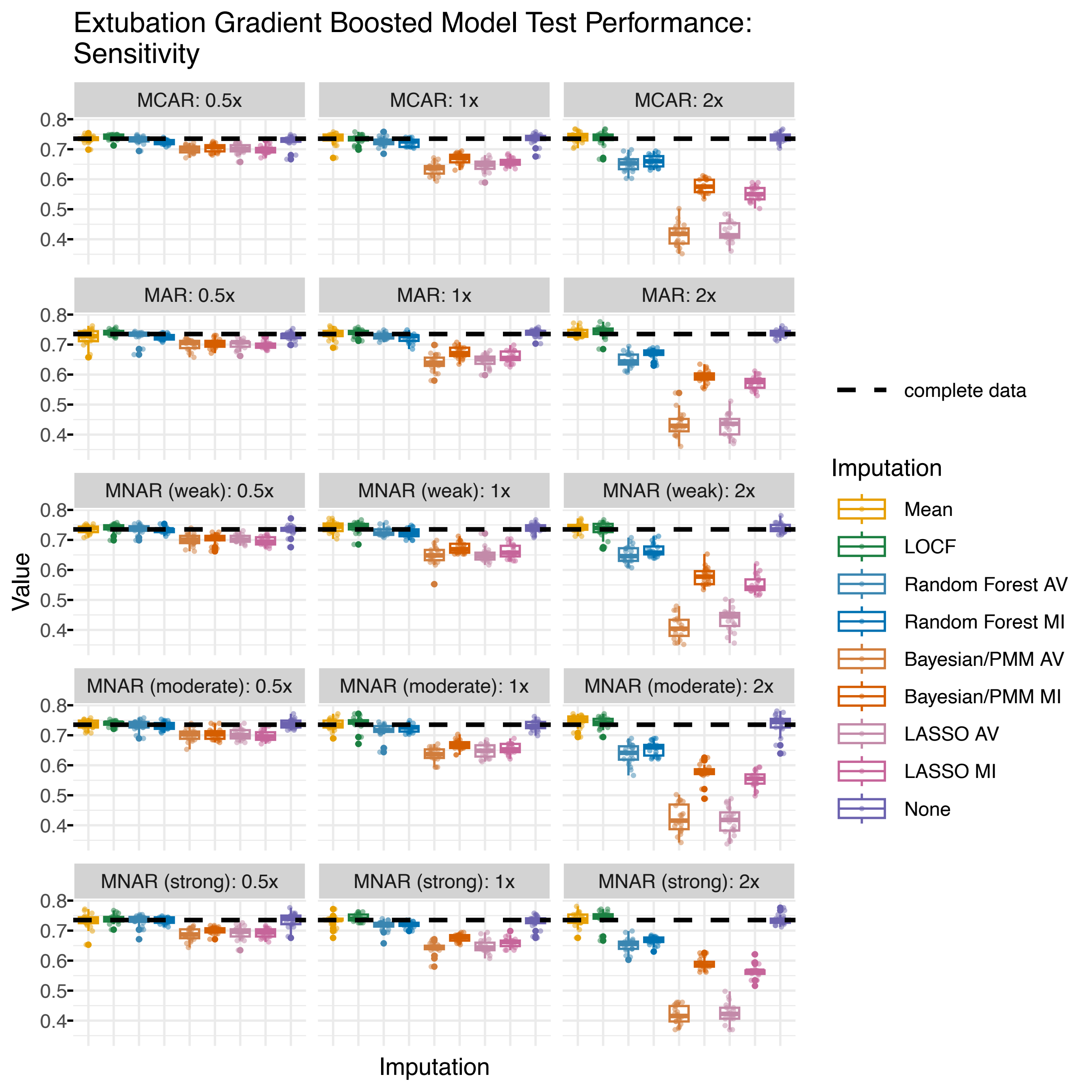
Extubation Gradient Boosted Model Test Performance:
Sensitivity
MCAR: 0.5x
MCAR: 1x
MCAR: 2x
MAR: 0.5x
MAR: 1x
MAR: 2x
MNAR (weak): 0.5x
MNAR (weak): 1x
MNAR (weak): 2x
MNAR (moderate): 0.5x
MNAR (moderate): 1x
MNAR (moderate): 2x
MNAR (strong): 0.5x
MNAR (strong): 1x
MNAR (strong): 2x
0.8
0.7
0.6
0.5
0.4
Value
Imputation
complete data
Imputation
Mean
LOCF
Random Forest AV
Random Forest MI
Bayesian/PMM AV
Bayesian/PMM MI
LASSO AV
LASSO MI
None

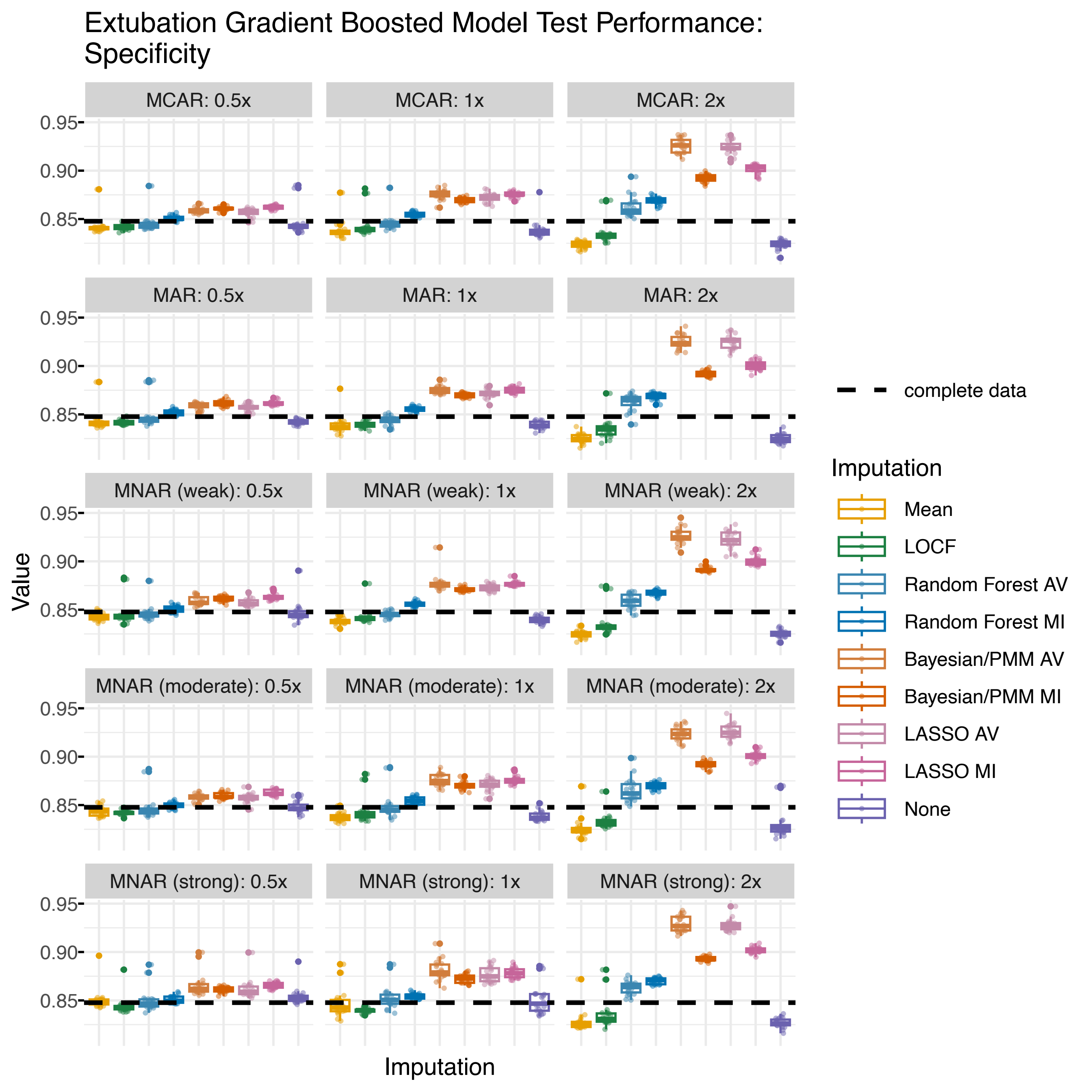

Extubation Gradient Boosted Model Test Performance: Specificity
MCAR: 0.5x
MCAR: 1x
MCAR: 2x
MAR: 0.5x
MAR: 1x
MAR: 2x
MNAR (weak): 0.5x
MNAR (weak): 1x
MNAR (weak): 2x
MNAR (moderate): 0.5x
MNAR (moderate): 1x
MNAR (moderate): 2x
MNAR (strong): 0.5x
MNAR (strong): 1x
MNAR (strong): 2x
0.95
0.90
0.85
Value
Imputation
complete data
Imputation
Mean
LOCF
Random Forest AV
Random Forest MI
Bayesian/PMM AV
Bayesian/PMM MI
LASSO AV
LASSO MI
None

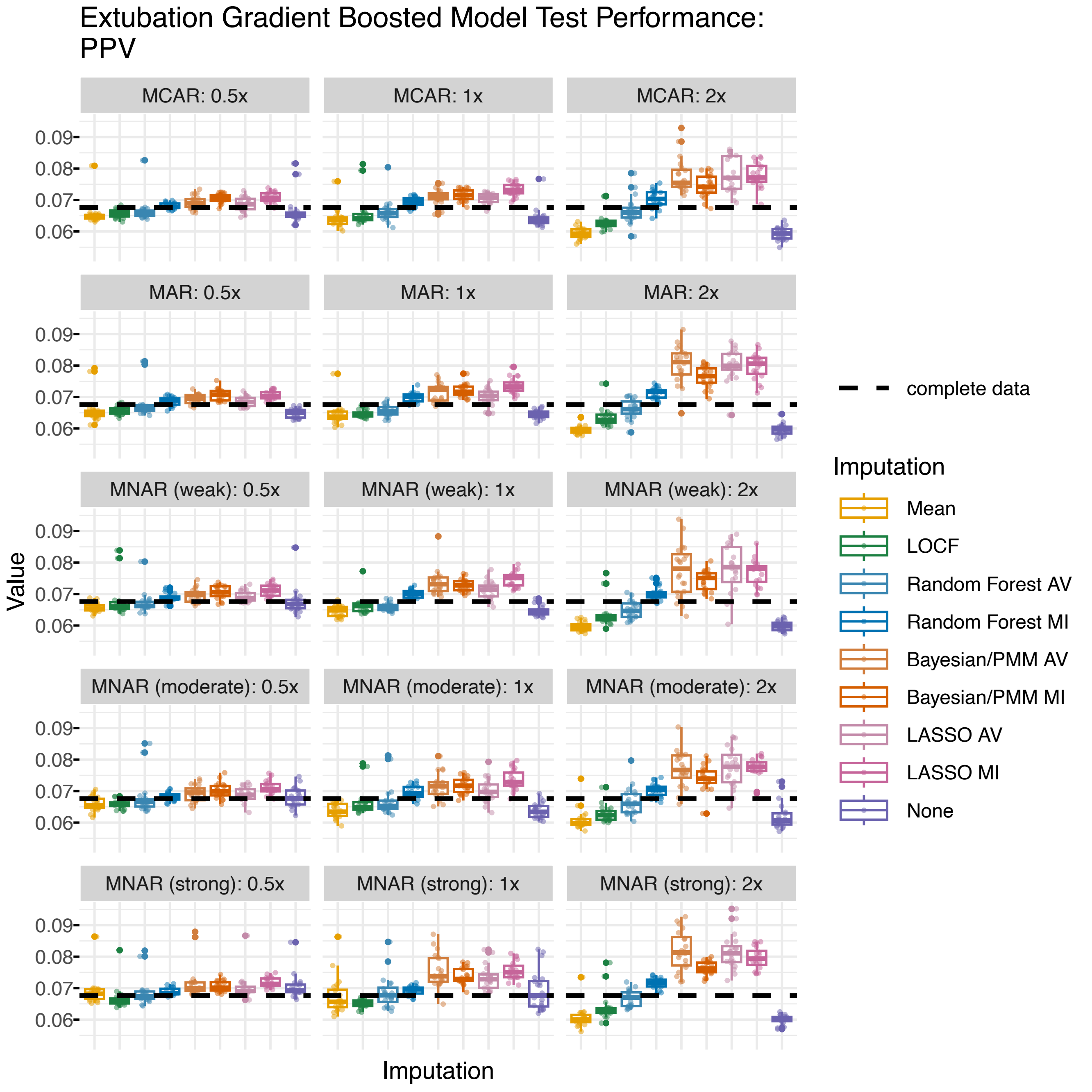
Extubation Gradient Boosted Model Test Performance: PPV
MCAR: 0.5x
MCAR: 1x
MCAR: 2x
MAR: 0.5x
MAR: 1x
MAR: 2x
MNAR (weak): 0.5x
MNAR (weak): 1x
MNAR (weak): 2x
MNAR (moderate): 0.5x
MNAR (moderate): 1x
MNAR (moderate): 2x
MNAR (strong): 0.5x
MNAR (strong): 1x
MNAR (strong): 2x
0.09
0.08
0.07
0.06
Value
Imputation
complete data
Imputation
Mean
LOCF
Random Forest AV
Random Forest MI
Bayesian/PMM AV
Bayesian/PMM MI
LASSO AV
LASSO MI
None

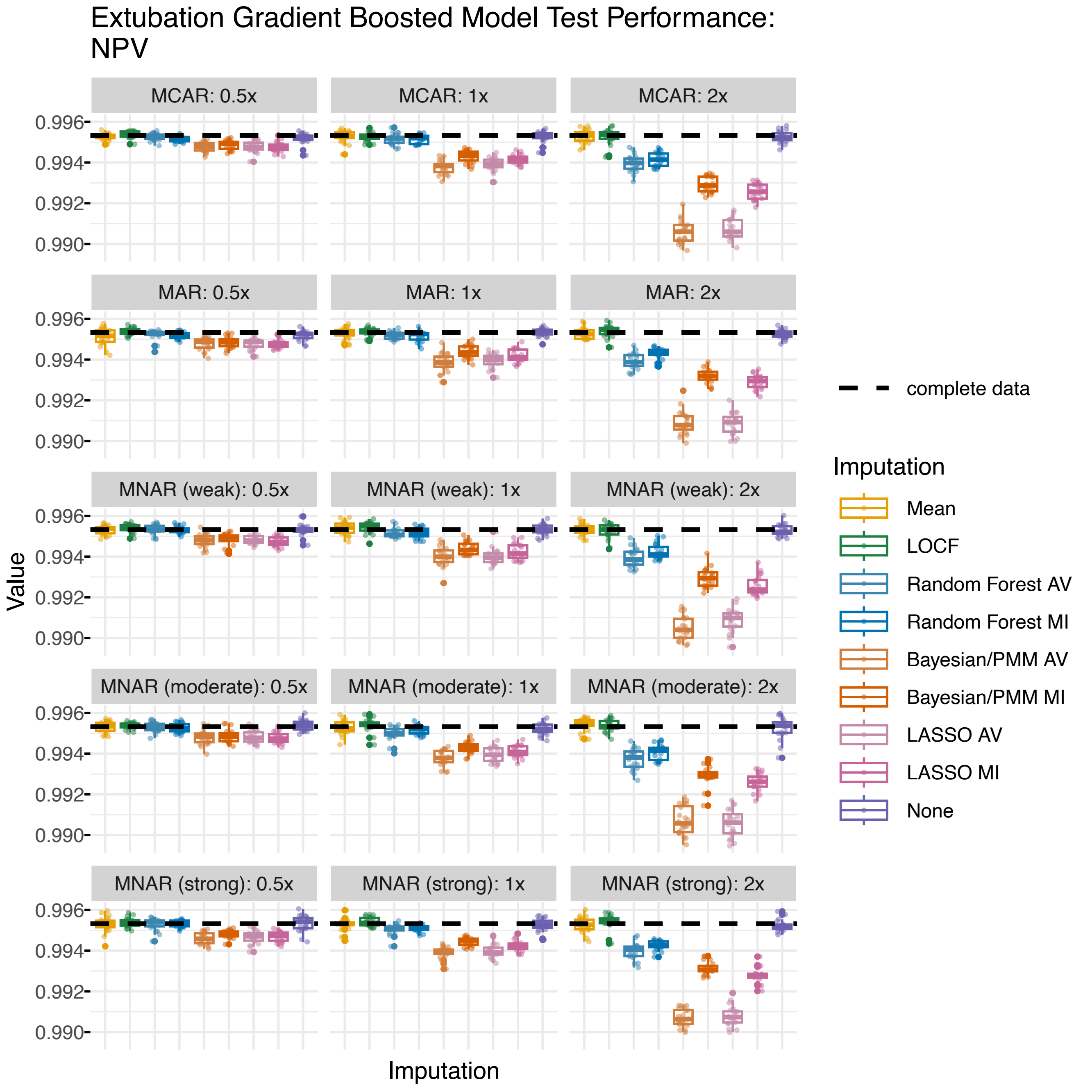

Extubation Gradient Boosted Model Test Performance:
NPV
MCAR: 0.5x
MCAR: 1x
MCAR: 2x
MAR: 0.5x
MAR: 1x
MAR: 2x
MNAR (weak): 0.5x
MNAR (weak): 1x
MNAR (weak): 2x
MNAR (moderate): 0.5x
MNAR (moderate): 1x
MNAR (moderate): 2x
MNAR (strong): 0.5x
MNAR (strong): 1x
MNAR (strong): 2x
0.996
0.994
0.992
0.990
Value
Imputation
complete data
Imputation
Mean
LOCF
Random Forest AV
Random Forest MI
Bayesian/PMM AV
Bayesian/PMM MI
LASSO AV
LASSO MI
None

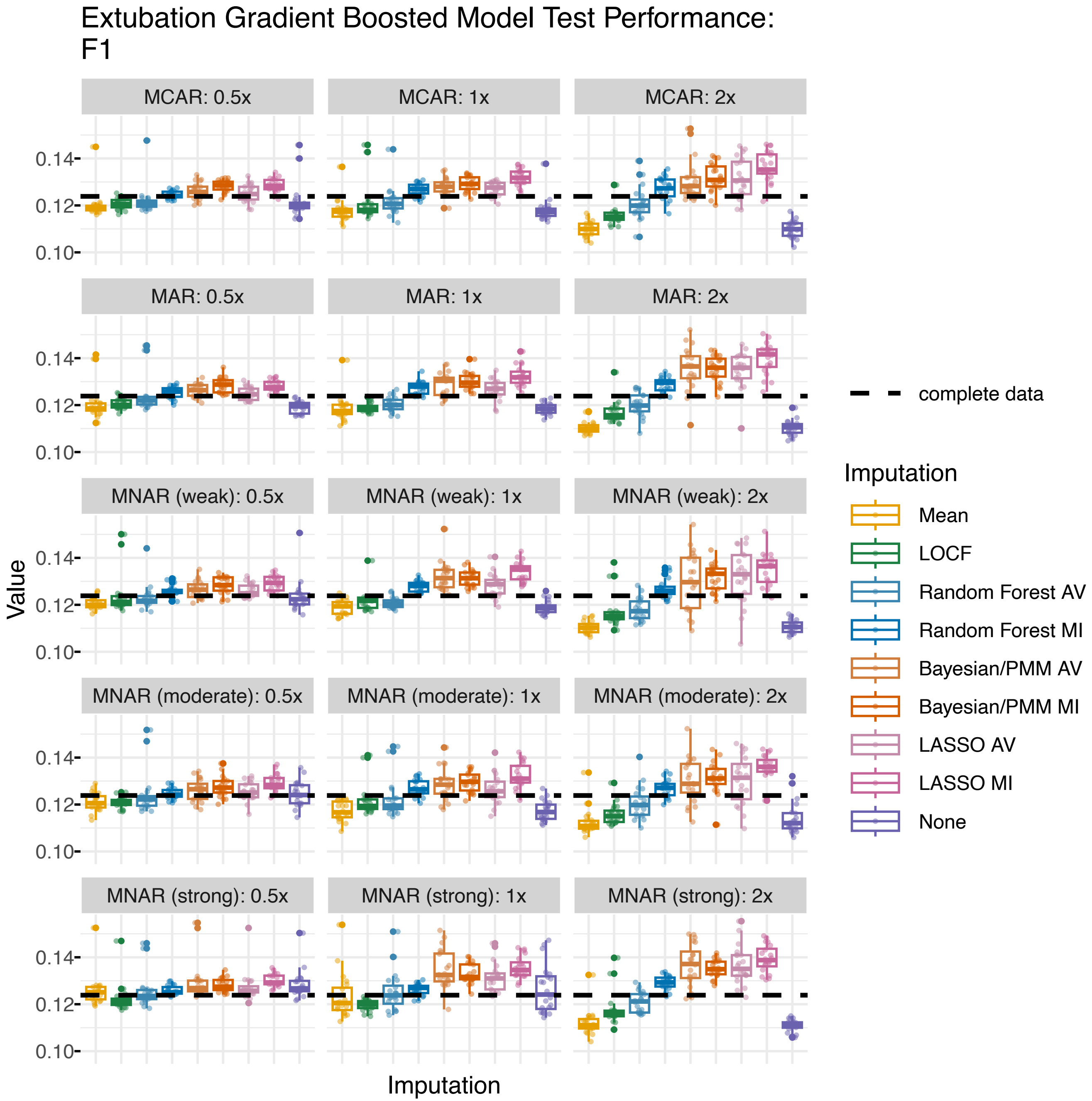

Extubation Gradient Boosted Model Test Performance:
F1
MCAR: 0.5x
MCAR: 1x
MCAR: 2x
MAR: 0.5x
MAR: 1x
MAR: 2x
MNAR (weak): 0.5x
MNAR (weak): 1x
MNAR (weak): 2x
MNAR (moderate): 0.5x
MNAR (moderate): 1x
MNAR (moderate): 2x
MNAR (strong): 0.5x
MNAR (strong): 1x
MNAR (strong): 2x
0.14
0.12
0.10
Value
Imputation
complete data
Imputation
Mean
LOCF
Random Forest AV
Random Forest MI
Bayesian/PMM AV
Bayesian/PMM MI
LASSO AV
LASSO MI
None
