# Supplementary material for: Methods for Addressing Missingness in Electronic Health Record Data for Clinical Prediction Models: Comparative Evaluation
Source: JMIR Med Inform. 2025 Nov 14;13:e79307. doi: 10.2196/79307 (PMC12617989; doi:10.2196/79307)
Supplement: Multimedia Appendix 12 [file medinform-v13-e79307-s012.pdf]

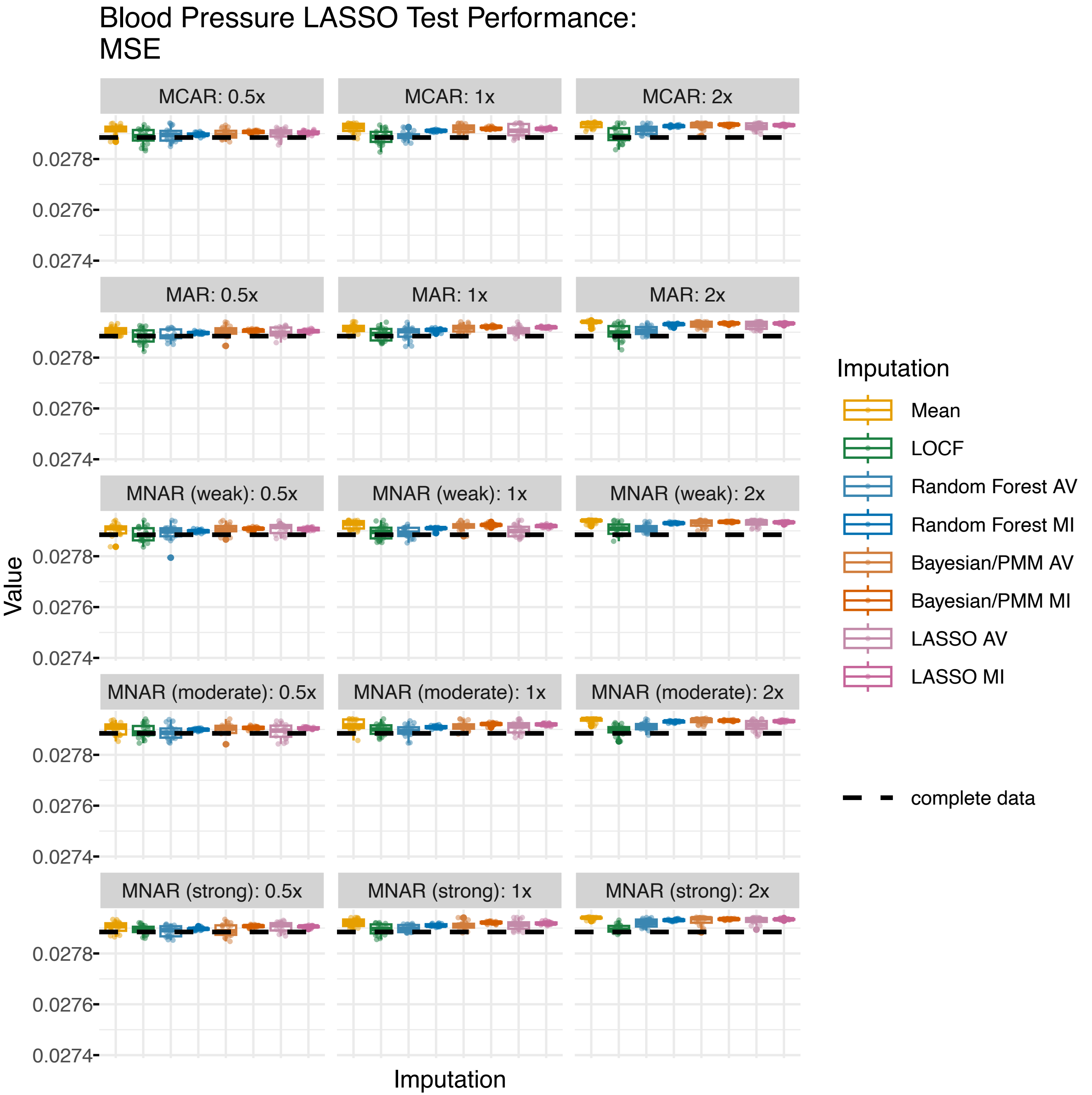

Blood Pressure LASSO Test Performance:
MSE
MCAR: 0.5x
MCAR: 1x
MCAR: 2x
MAR: 0.5x
MAR: 1x
MAR: 2x
MNAR (weak): 0.5x
MNAR (weak): 1x
MNAR (weak): 2x
MNAR (moderate): 0.5x
MNAR (moderate): 1x
MNAR (moderate): 2x
MNAR (strong): 0.5x
MNAR (strong): 1x
MNAR (strong): 2x
0.0278
0.0276
0.0274
Value
Imputation
Imputation
Mean
LOCF
Random Forest AV
Random Forest MI
Bayesian/PMM AV
Bayesian/PMM MI
LASSO AV
LASSO MI
complete data

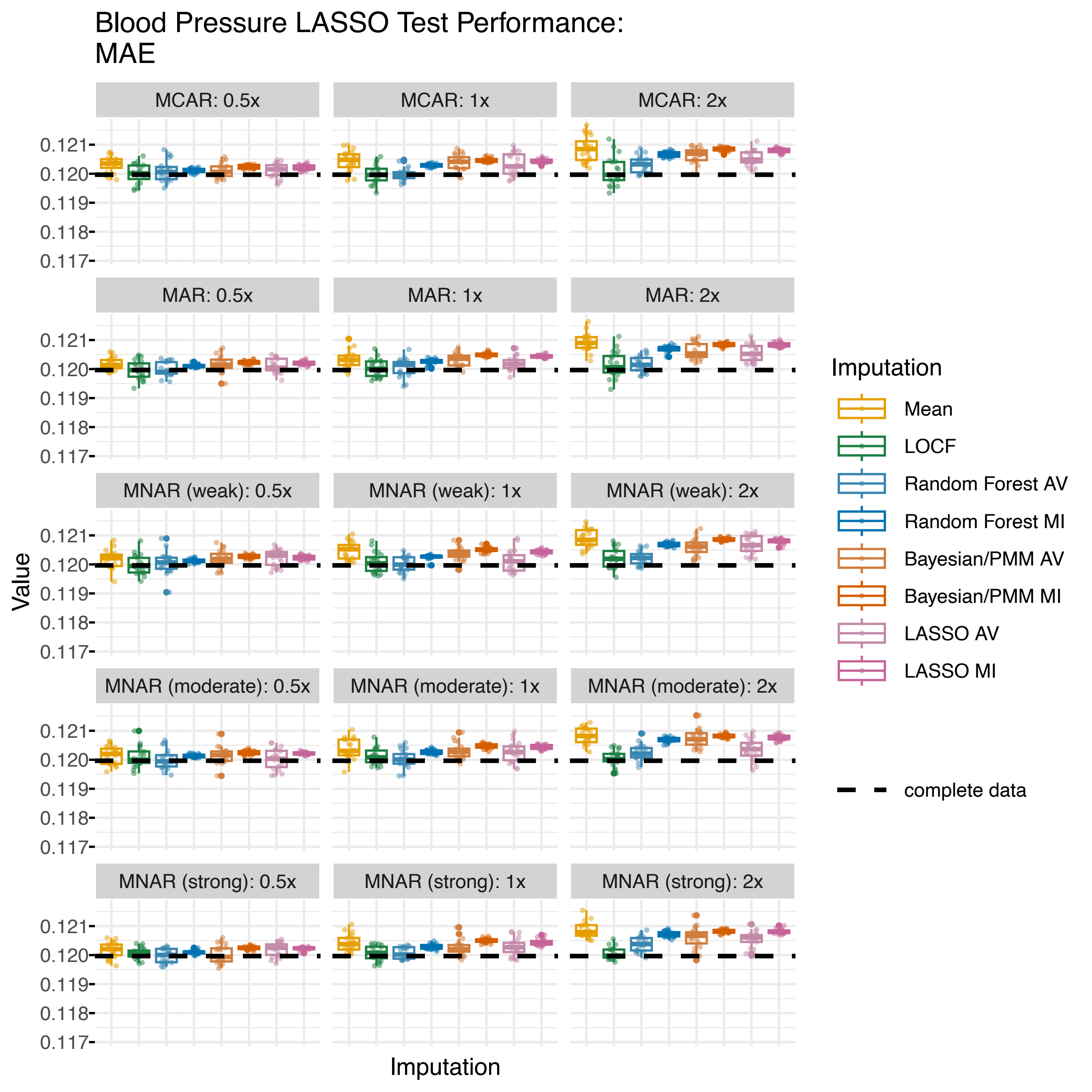

Blood Pressure LASSO Test Performance:
MAE
MCAR: 0.5x
MCAR: 1x
MCAR: 2x
MAR: 0.5x
MAR: 1x
MAR: 2x
MNAR (weak): 0.5x
MNAR (weak): 1x
MNAR (weak): 2x
MNAR (moderate): 0.5x
MNAR (moderate): 1x
MNAR (moderate): 2x
MNAR (strong): 0.5x
MNAR (strong): 1x
MNAR (strong): 2x
0.121
0.120
0.119
0.118
0.117
Value
Imputation
Imputation
Mean
LOCF
Random Forest AV
Random Forest MI
Bayesian/PMM AV
Bayesian/PMM MI
LASSO AV
LASSO MI
complete data

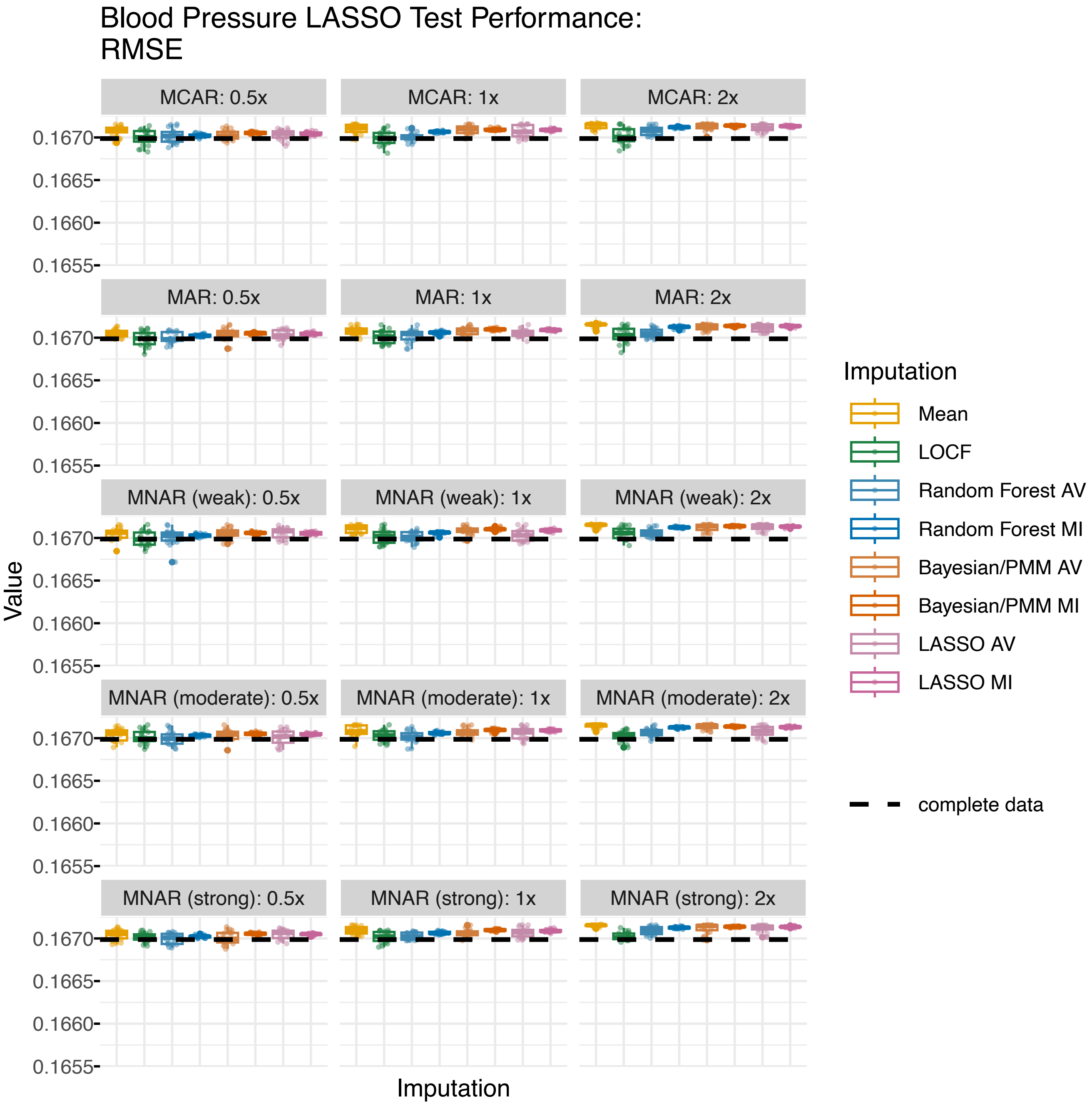

Blood Pressure LASSO Test Performance:
RMSE
MCAR: 0.5x
MCAR: 1x
MCAR: 2x
MAR: 0.5x
MAR: 1x
MAR: 2x
MNAR (weak): 0.5x
MNAR (weak): 1x
MNAR (weak): 2x
MNAR (moderate): 0.5x
MNAR (moderate): 1x
MNAR (moderate): 2x
MNAR (strong): 0.5x
MNAR (strong): 1x
MNAR (strong): 2x
0.1670
0.1665
0.1660
0.1655
Value
Imputation
Imputation
Mean
LOCF
Random Forest AV
Random Forest MI
Bayesian/PMM AV
Bayesian/PMM MI
LASSO AV
LASSO MI
complete data

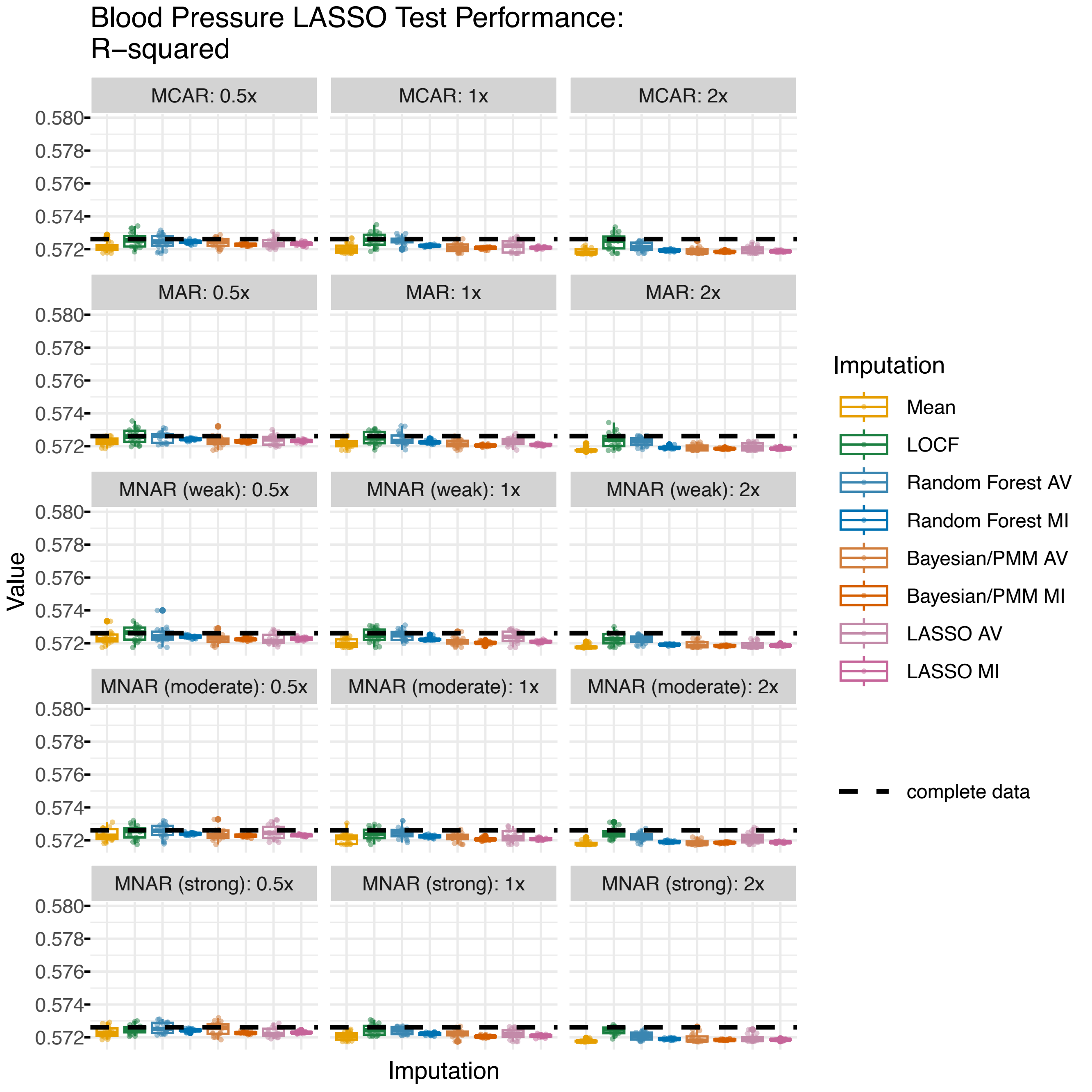
Blood Pressure LASSO Test Performance:
R−squared
MCAR: 0.5x
MCAR: 1x
MCAR: 2x
MAR: 0.5x
MAR: 1x
MAR: 2x
MNAR (weak): 0.5x
MNAR (weak): 1x
MNAR (weak): 2x
MNAR (moderate): 0.5x
MNAR (moderate): 1x
MNAR (moderate): 2x
MNAR (strong): 0.5x
MNAR (strong): 1x
MNAR (strong): 2x
0.580
0.578
0.576
0.574
0.572
Value
Imputation
Imputation
Mean
LOCF
Random Forest AV
Random Forest MI
Bayesian/PMM AV
Bayesian/PMM MI
LASSO AV
LASSO MI
complete data
