# Supplementary material for: Methods for Addressing Missingness in Electronic Health Record Data for Clinical Prediction Models: Comparative Evaluation
Source: JMIR Med Inform. 2025 Nov 14;13:e79307. doi: 10.2196/79307 (PMC12617989; doi:10.2196/79307)
Supplement: Multimedia Appendix 14 [file medinform-v13-e79307-s014.pdf]

# Extubation LASSO Difference Between Train and Test: Balanced Accuracy

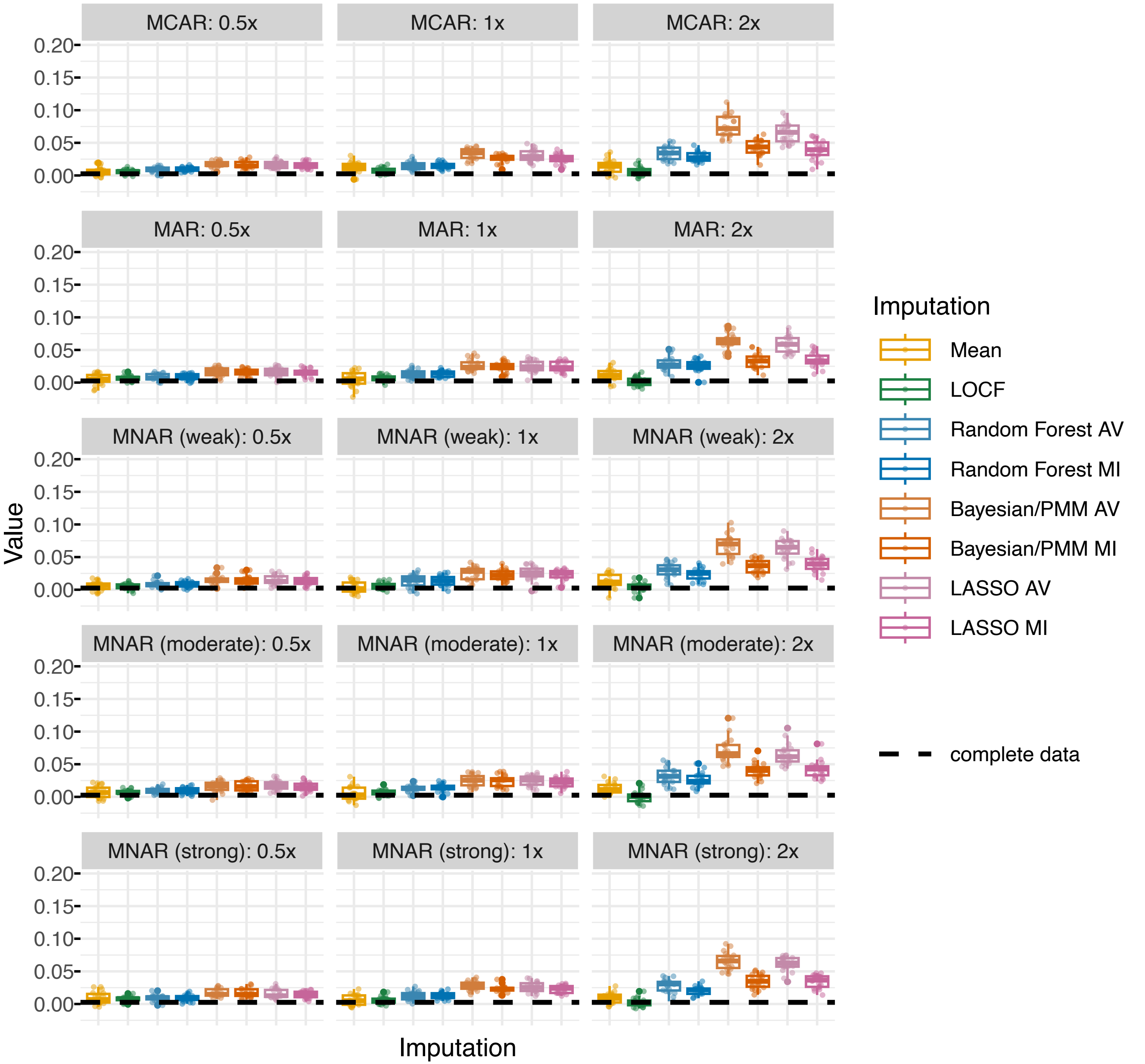

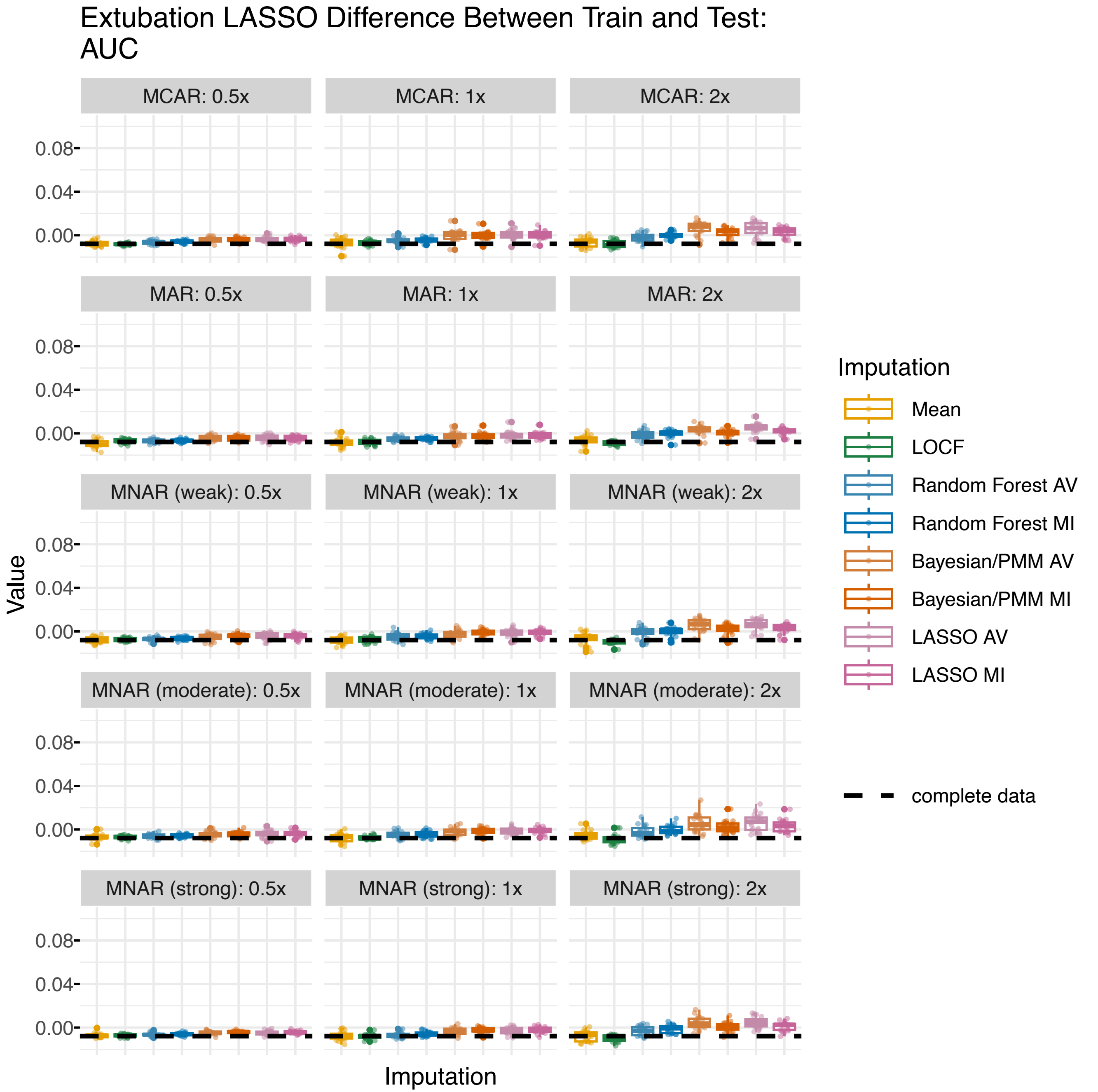

Extubation LASSO Difference Between Train and Test:
AUC
MCAR: 0.5x
MCAR: 1x
MCAR: 2x
MAR: 0.5x
MAR: 1x
MAR: 2x
MNAR (weak): 0.5x
MNAR (weak): 1x
MNAR (weak): 2x
MNAR (moderate): 0.5x
MNAR (moderate): 1x
MNAR (moderate): 2x
MNAR (strong): 0.5x
MNAR (strong): 1x
MNAR (strong): 2x
0.08
0.04
0.00
Value
Imputation
Imputation
Mean
LOCF
Random Forest AV
Random Forest MI
Bayesian/PMM AV
Bayesian/PMM MI
LASSO AV
LASSO MI
complete data

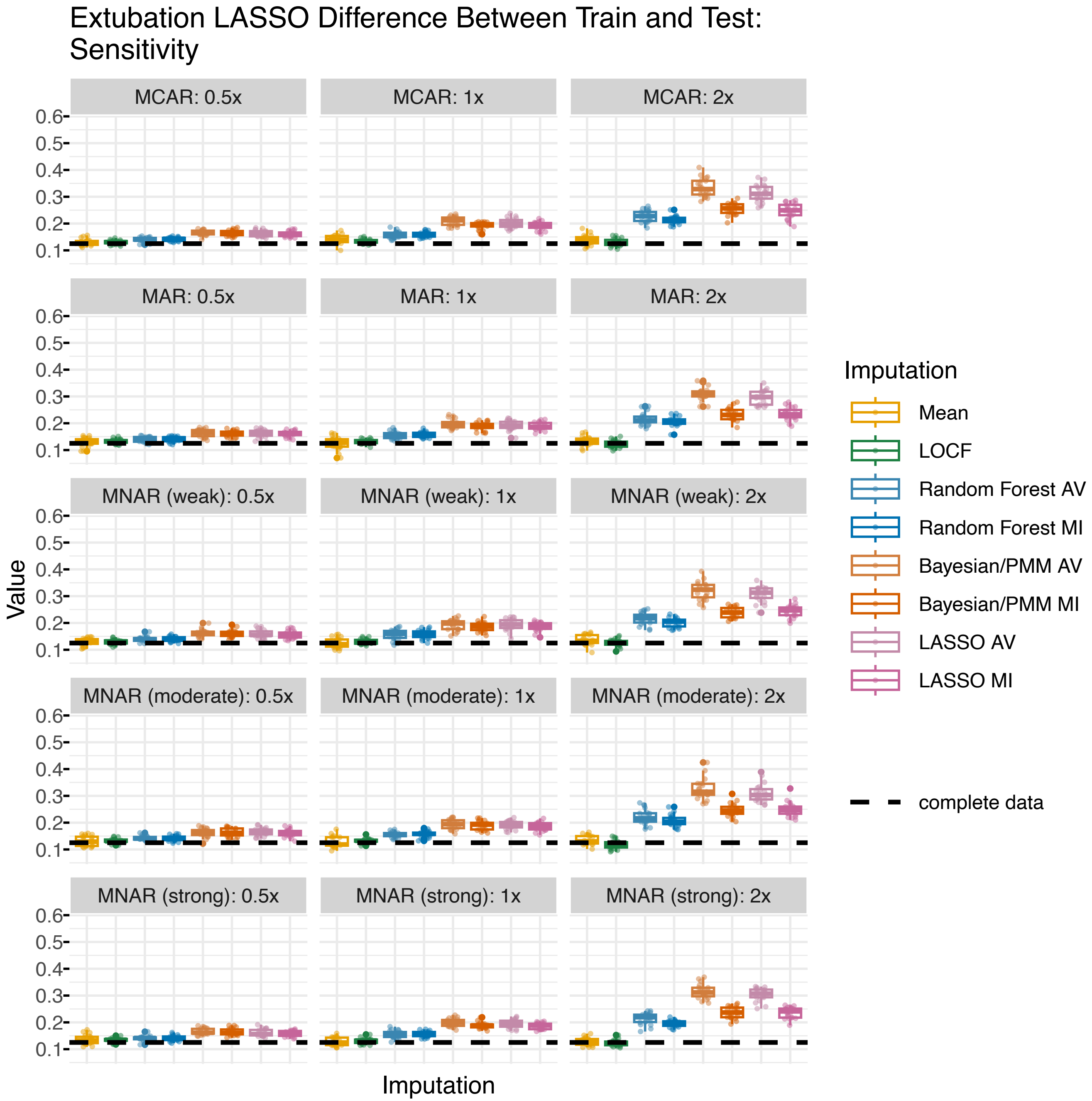

Extubation LASSO Difference Between Train and Test: Sensitivity
MCAR: 0.5x
MCAR: 1x
MCAR: 2x
MAR: 0.5x
MAR: 1x
MAR: 2x
MNAR (weak): 0.5x
MNAR (weak): 1x
MNAR (weak): 2x
MNAR (moderate): 0.5x
MNAR (moderate): 1x
MNAR (moderate): 2x
MNAR (strong): 0.5x
MNAR (strong): 1x
MNAR (strong): 2x
0.6
0.5
0.4
0.3
0.2
0.1
Value
Imputation
Imputation
Mean
LOCF
Random Forest AV
Random Forest MI
Bayesian/PMM AV
Bayesian/PMM MI
LASSO AV
LASSO MI
complete data

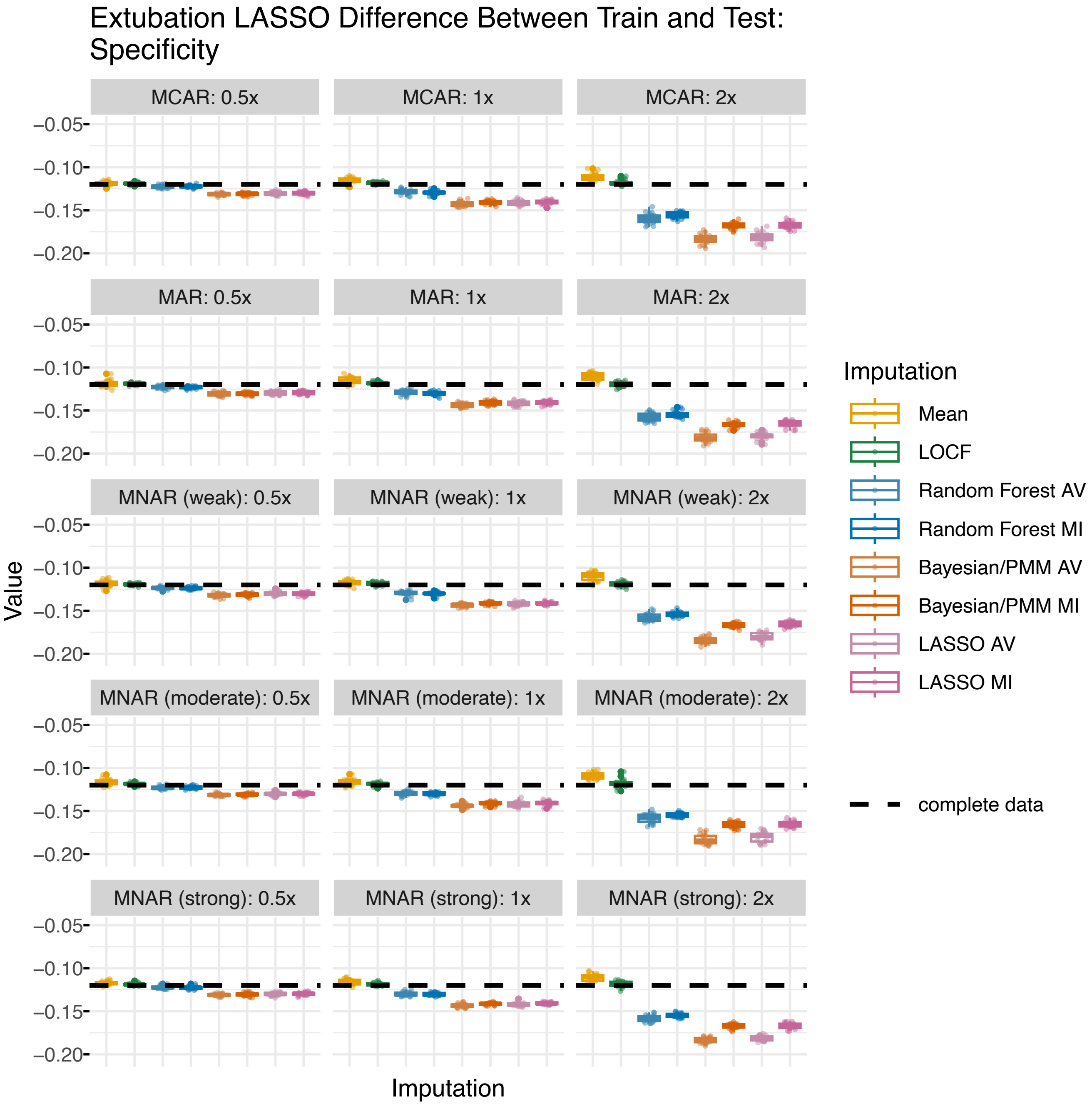
Extubation LASSO Difference Between Train and Test: Specificity
MCAR: 0.5x
MCAR: 1x
MCAR: 2x
MAR: 0.5x
MAR: 1x
MAR: 2x
MNAR (weak): 0.5x
MNAR (weak): 1x
MNAR (weak): 2x
MNAR (moderate): 0.5x
MNAR (moderate): 1x
MNAR (moderate): 2x
MNAR (strong): 0.5x
MNAR (strong): 1x
MNAR (strong): 2x
−0.05
−0.10
−0.15
−0.20
Value
Imputation
Imputation
Mean
LOCF
Random Forest AV
Random Forest MI
Bayesian/PMM AV
Bayesian/PMM MI
LASSO AV
LASSO MI
complete data

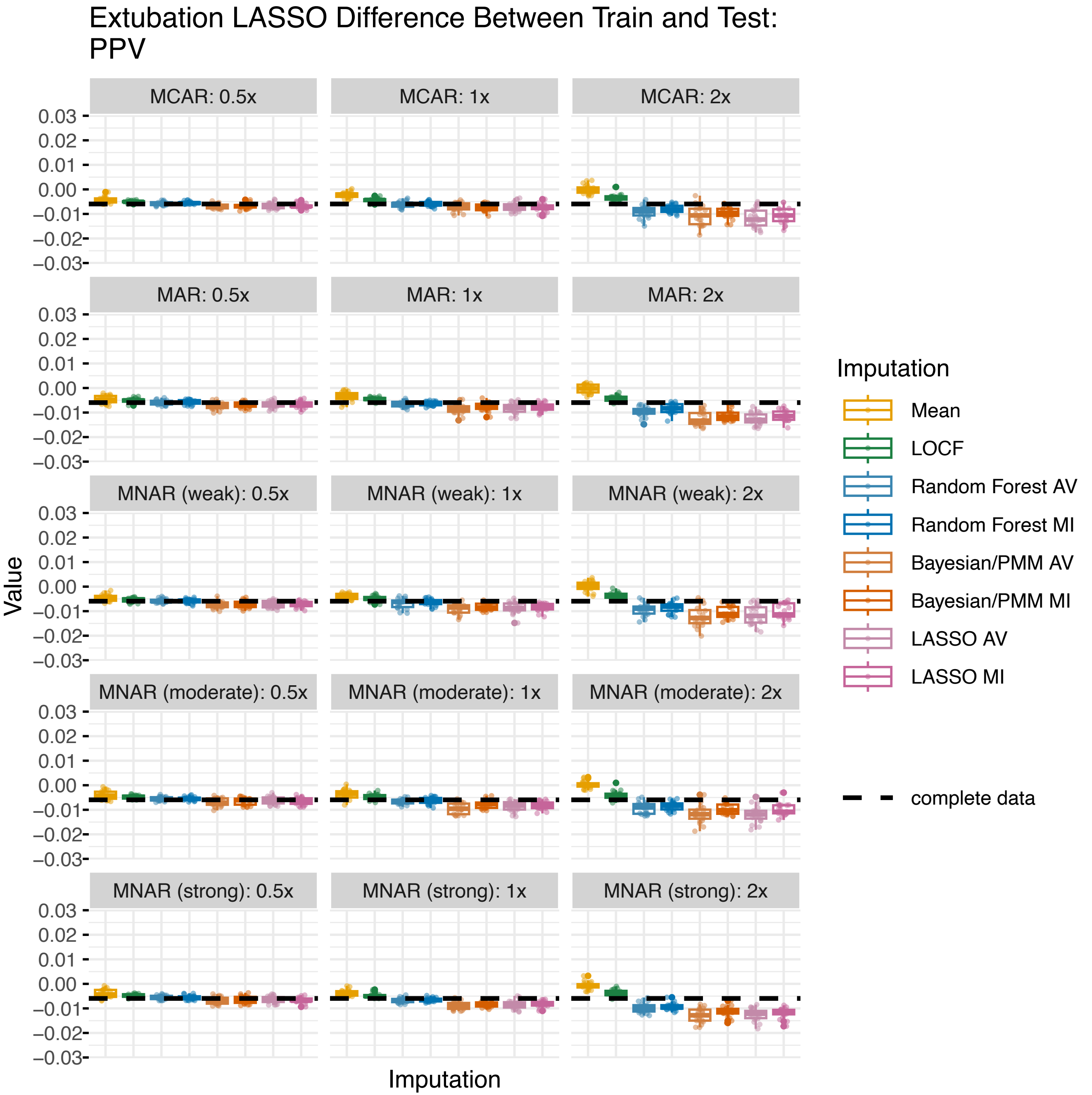
Extubation LASSO Difference Between Train and Test:
PPV
MCAR: 0.5x
MCAR: 1x
MCAR: 2x
MAR: 0.5x
MAR: 1x
MAR: 2x
MNAR (weak): 0.5x
MNAR (weak): 1x
MNAR (weak): 2x
MNAR (moderate): 0.5x
MNAR (moderate): 1x
MNAR (moderate): 2x
MNAR (strong): 0.5x
MNAR (strong): 1x
MNAR (strong): 2x
0.03
0.02
0.01
0.00
−0.01
−0.02
−0.03
Value
Imputation
Imputation
Mean
LOCF
Random Forest AV
Random Forest MI
Bayesian/PMM AV
Bayesian/PMM MI
LASSO AV
LASSO MI
complete data

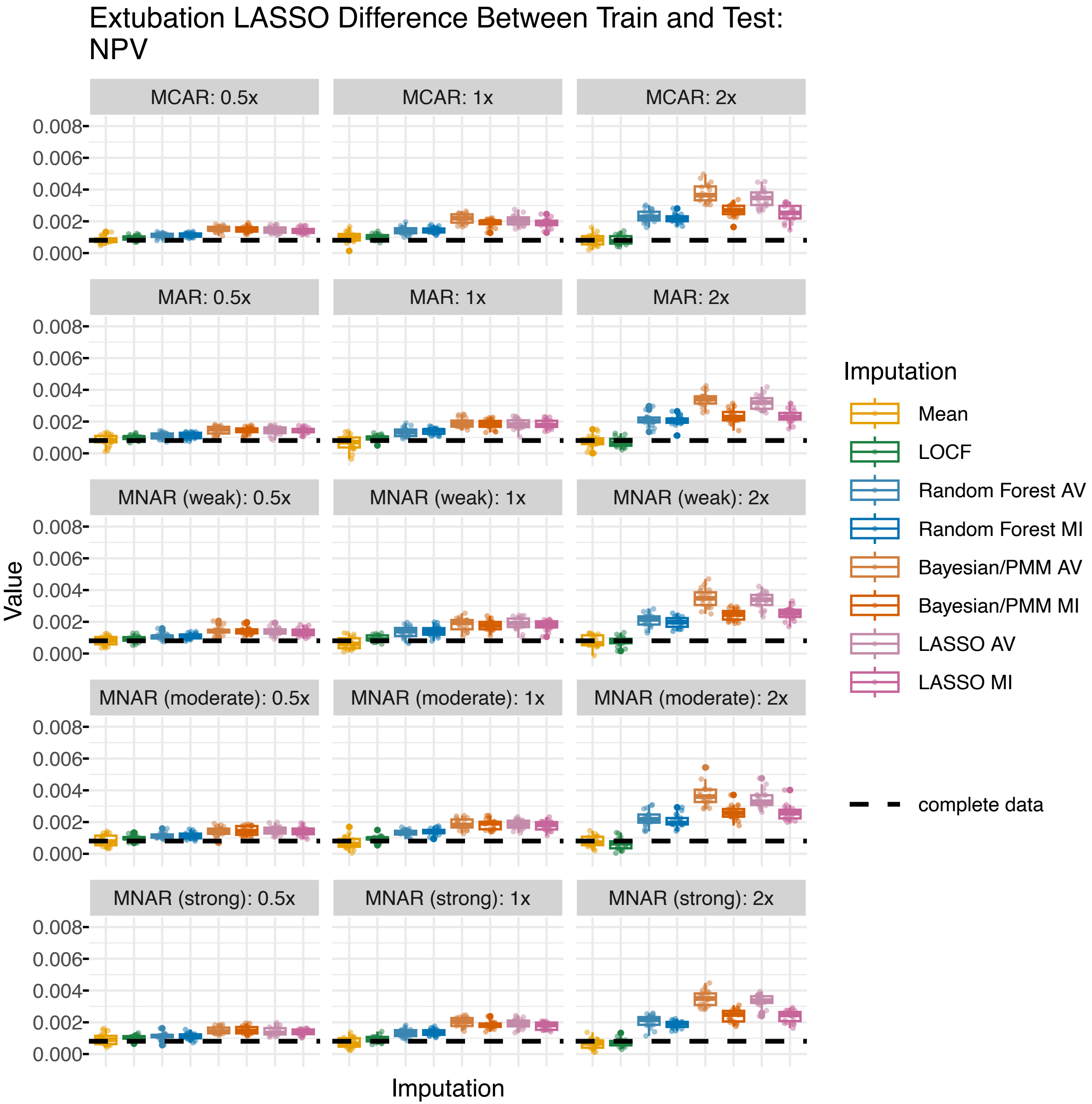
Extubation LASSO Difference Between Train and Test:
NPV
MCAR: 0.5x
MCAR: 1x
MCAR: 2x
MAR: 0.5x
MAR: 1x
MAR: 2x
MNAR (weak): 0.5x
MNAR (weak): 1x
MNAR (weak): 2x
MNAR (moderate): 0.5x
MNAR (moderate): 1x
MNAR (moderate): 2x
MNAR (strong): 0.5x
MNAR (strong): 1x
MNAR (strong): 2x
0.008
0.006
0.004
0.002
0.000
Value
Imputation
Imputation
Mean
LOCF
Random Forest AV
Random Forest MI
Bayesian/PMM AV
Bayesian/PMM MI
LASSO AV
LASSO MI
complete data

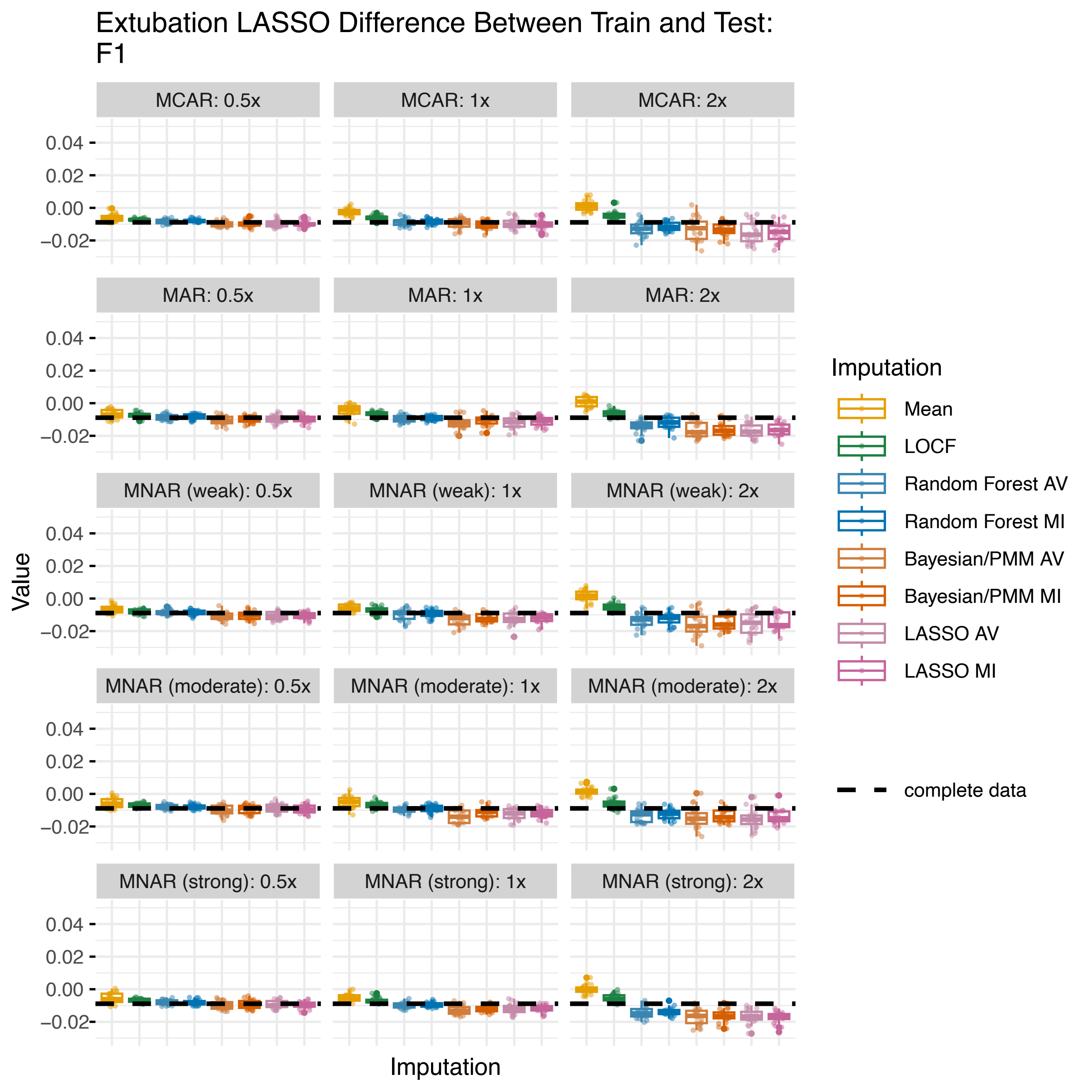
Extubation LASSO Difference Between Train and Test:
F1
MCAR: 0.5x
MCAR: 1x
MCAR: 2x
MAR: 0.5x
MAR: 1x
MAR: 2x
MNAR (weak): 0.5x
MNAR (weak): 1x
MNAR (weak): 2x
MNAR (moderate): 0.5x
MNAR (moderate): 1x
MNAR (moderate): 2x
MNAR (strong): 0.5x
MNAR (strong): 1x
MNAR (strong): 2x
0.04
0.02
0.00
−0.02
Value
Imputation
Imputation
Mean
LOCF
Random Forest AV
Random Forest MI
Bayesian/PMM AV
Bayesian/PMM MI
LASSO AV
LASSO MI
complete data
