# Supplementary material for: Methods for Addressing Missingness in Electronic Health Record Data for Clinical Prediction Models: Comparative Evaluation
Source: JMIR Med Inform. 2025 Nov 14;13:e79307. doi: 10.2196/79307 (PMC12617989; doi:10.2196/79307)
Supplement: Multimedia Appendix 15 [file medinform-v13-e79307-s015.pdf]

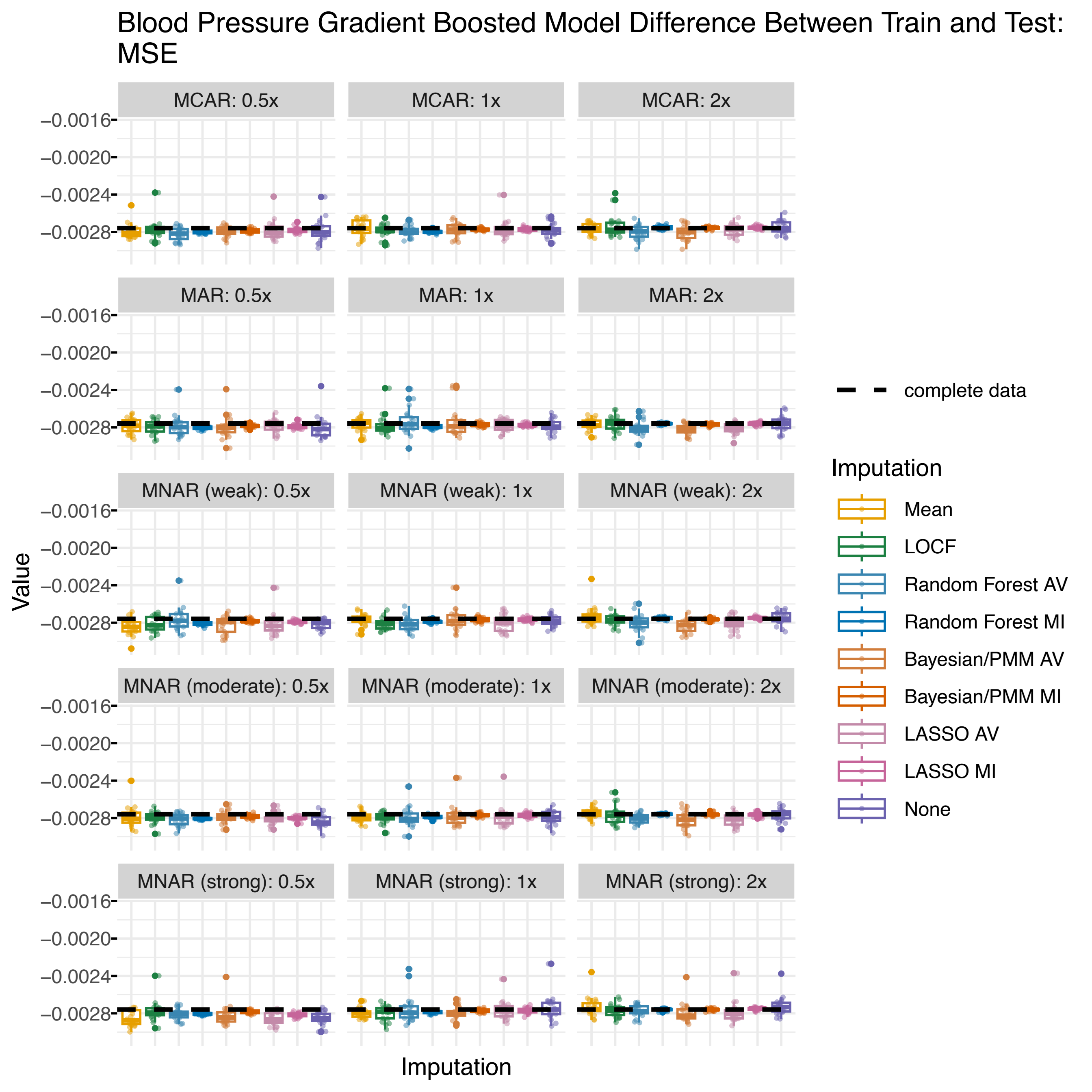

Blood Pressure Gradient Boosted Model Difference Between Train and Test: MSE
MCAR: 0.5x
MCAR: 1x
MCAR: 2x
MAR: 0.5x
MAR: 1x
MAR: 2x
MNAR (weak): 0.5x
MNAR (weak): 1x
MNAR (weak): 2x
MNAR (moderate): 0.5x
MNAR (moderate): 1x
MNAR (moderate): 2x
MNAR (strong): 0.5x
MNAR (strong): 1x
MNAR (strong): 2x
−0.0016
−0.0020
−0.0024
−0.0028
Value
Imputation
complete data
Imputation
Mean
LOCF
Random Forest AV
Random Forest MI
Bayesian/PMM AV
Bayesian/PMM MI
LASSO AV
LASSO MI
None

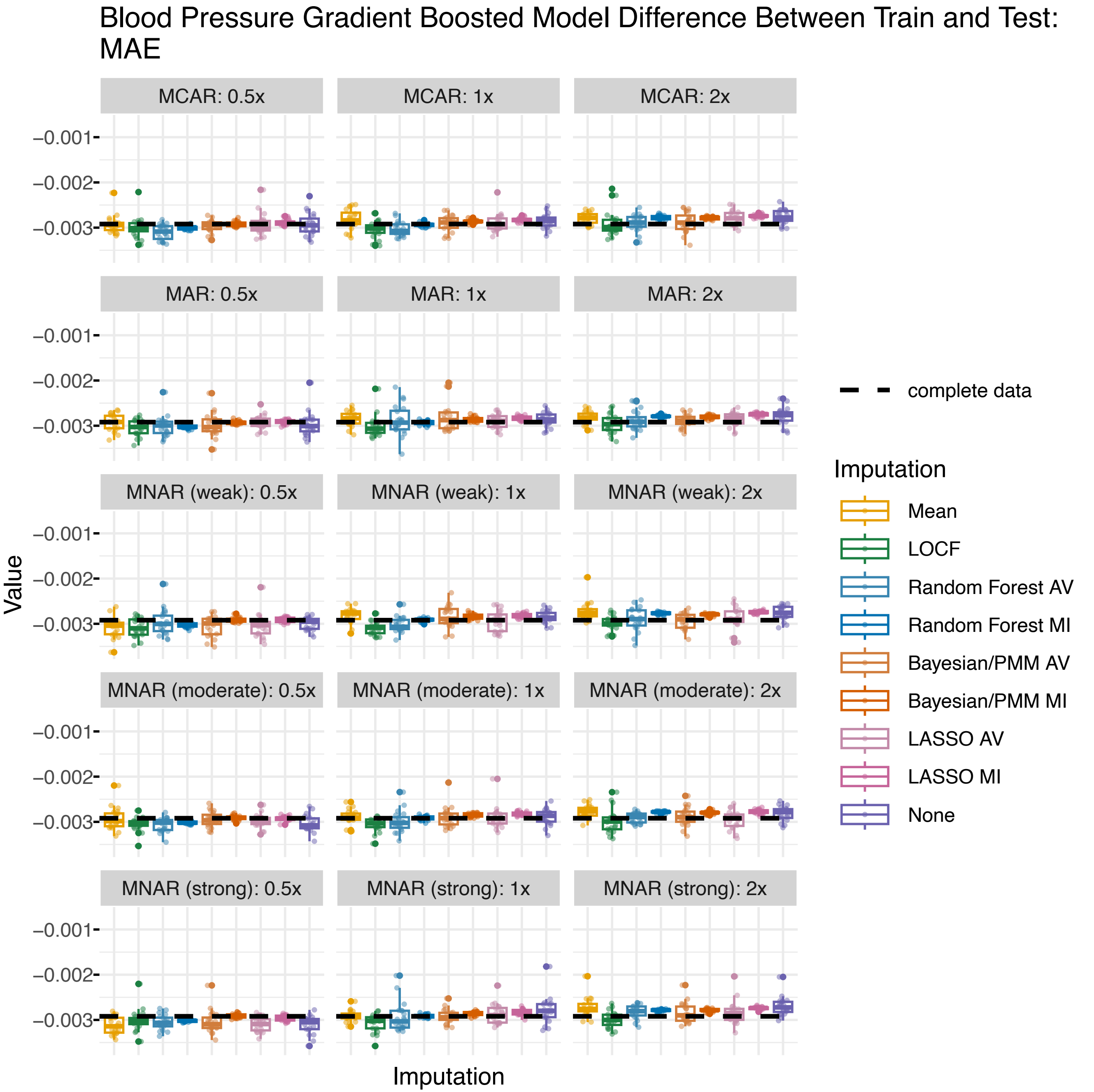
Blood Pressure Gradient Boosted Model Difference Between Train and Test: MAE
MCAR: 0.5x
MCAR: 1x
MCAR: 2x
MAR: 0.5x
MAR: 1x
MAR: 2x
MNAR (weak): 0.5x
MNAR (weak): 1x
MNAR (weak): 2x
MNAR (moderate): 0.5x
MNAR (moderate): 1x
MNAR (moderate): 2x
MNAR (strong): 0.5x
MNAR (strong): 1x
MNAR (strong): 2x
−0.001
−0.002
−0.003
Value
Imputation
complete data
Imputation
Mean
LOCF
Random Forest AV
Random Forest MI
Bayesian/PMM AV
Bayesian/PMM MI
LASSO AV
LASSO MI
None

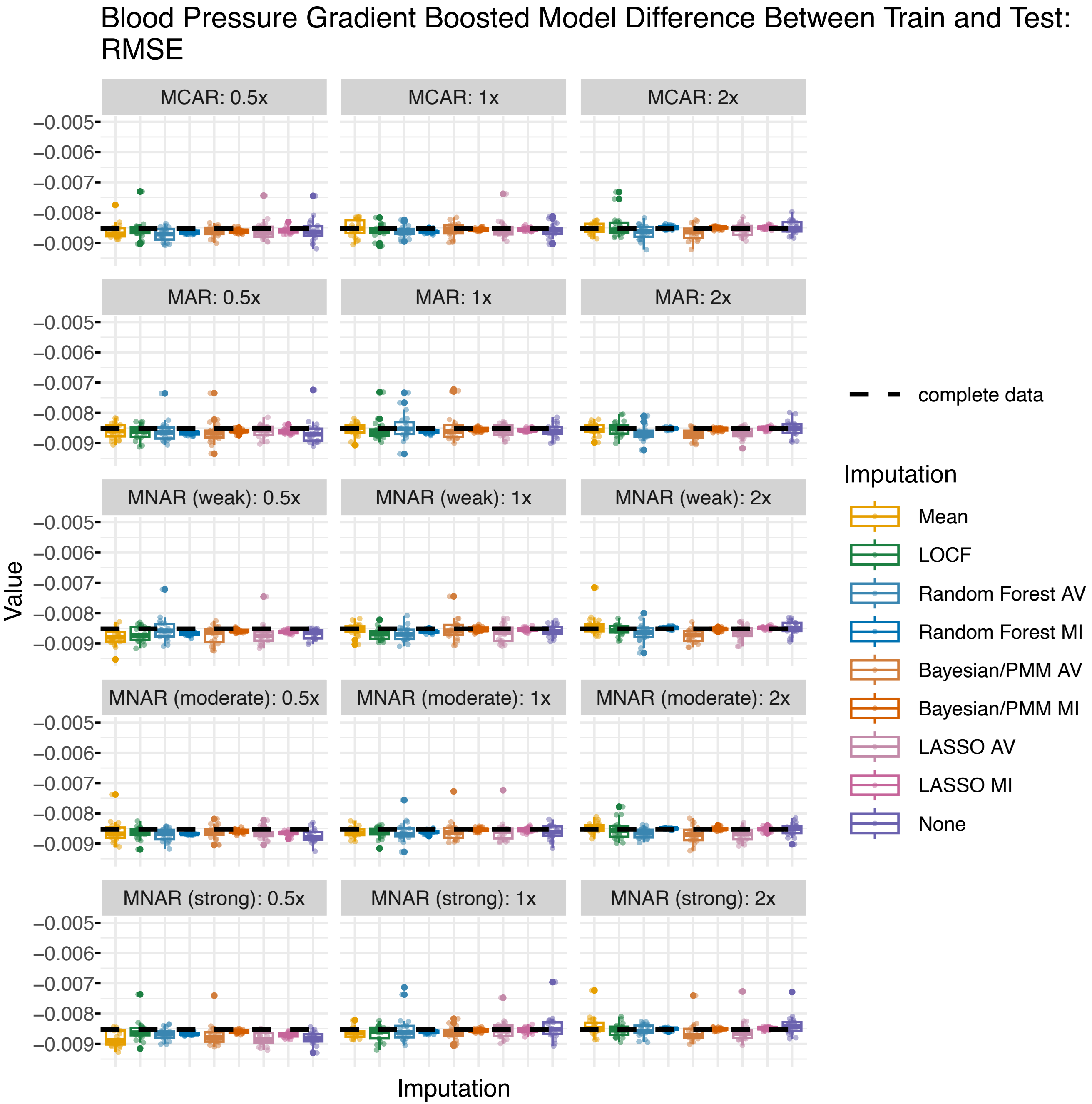
Blood Pressure Gradient Boosted Model Difference Between Train and Test: RMSE
MCAR: 0.5x
MCAR: 1x
MCAR: 2x
MAR: 0.5x
MAR: 1x
MAR: 2x
MNAR (weak): 0.5x
MNAR (weak): 1x
MNAR (weak): 2x
MNAR (moderate): 0.5x
MNAR (moderate): 1x
MNAR (moderate): 2x
MNAR (strong): 0.5x
MNAR (strong): 1x
MNAR (strong): 2x
−0.005
−0.006
−0.007
−0.008
−0.009
Value
Imputation
complete data
Imputation
Mean
LOCF
Random Forest AV
Random Forest MI
Bayesian/PMM AV
Bayesian/PMM MI
LASSO AV
LASSO MI
None

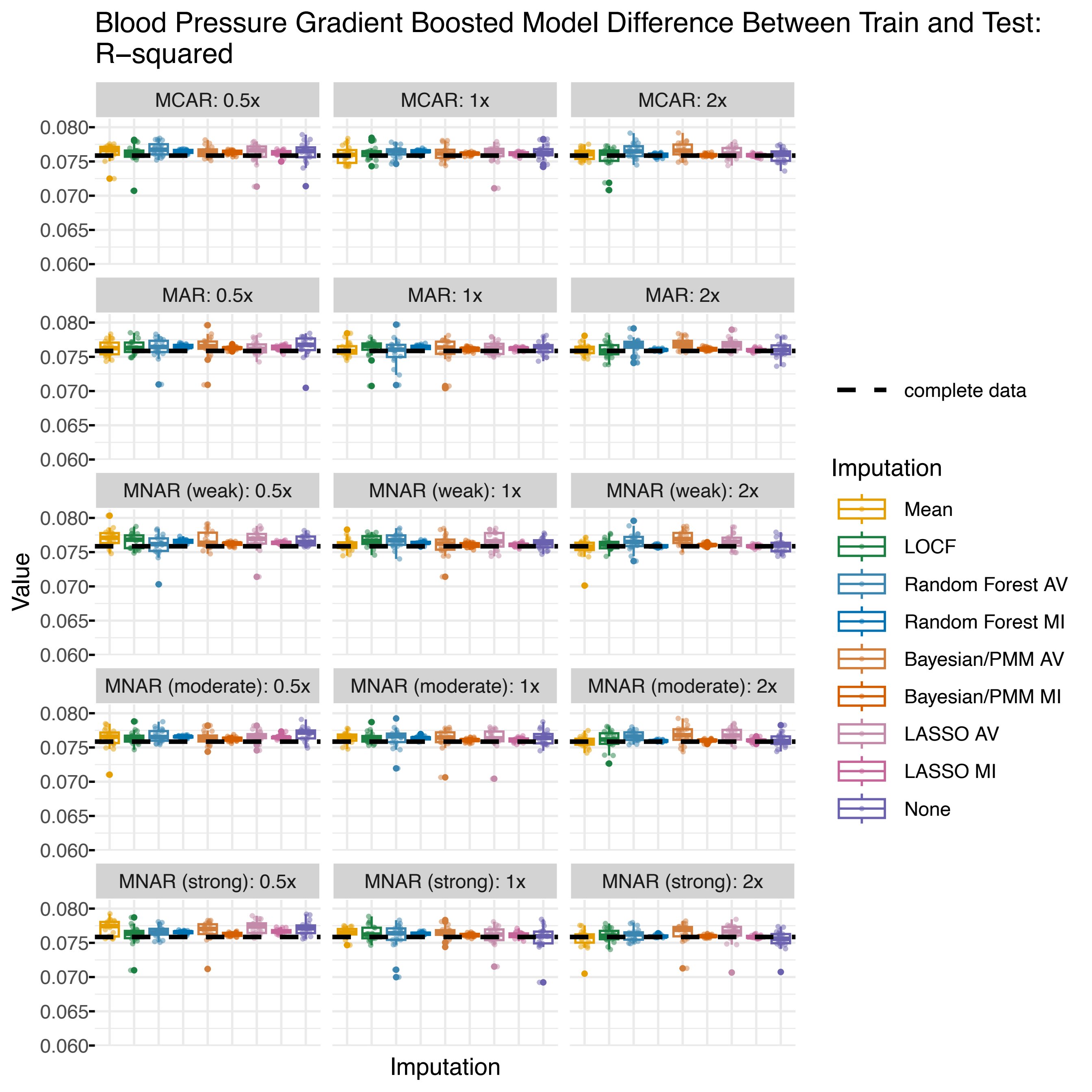
Blood Pressure Gradient Boosted Model Difference Between Train and Test: R−squared
MCAR: 0.5x
MCAR: 1x
MCAR: 2x
MAR: 0.5x
MAR: 1x
MAR: 2x
MNAR (weak): 0.5x
MNAR (weak): 1x
MNAR (weak): 2x
MNAR (moderate): 0.5x
MNAR (moderate): 1x
MNAR (moderate): 2x
MNAR (strong): 0.5x
MNAR (strong): 1x
MNAR (strong): 2x
0.080
0.075
0.070
0.065
0.060
Value
Imputation
complete data
Imputation
Mean
LOCF
Random Forest AV
Random Forest MI
Bayesian/PMM AV
Bayesian/PMM MI
LASSO AV
LASSO MI
None
